# Coumarin‐Augmented Thiazole Hybrids as Dual Anticancer and Antibacterial Agents

**DOI:** 10.1111/cbdd.70261

**Published:** 2026-02-20

**Authors:** Islam K. Matar, Magdi E. A. Zaki, Zeinab A. Muhammad, Dahlia A. Awwad, Sami A. Al‐Hussain, Chérif F. Matta, Refaie M. Kassab

**Affiliations:** ^1^ Department of Chemistry Saint Mary's University Halifax Nova Scotia Canada; ^2^ Department of Chemistry and Physics Mount Saint Vincent University Halifax Nova Scotia Canada; ^3^ Department of Chemistry, Faculty of Science Imam Mohammad Ibn Saud Islamic University (IMSIU) Riyadh South Africa; ^4^ Department of Pharmaceutical Chemistry Egyptian Drug Authority (EDA) Giza Egypt; ^5^ University of Science and Technology (UST), Zewail City of Science and Technology Giza Egypt; ^6^ Department of Chemistry, Faculty of Science Cairo University Giza Egypt

**Keywords:** antibacterial activity, anticancer agents, coumarin derivatives, DNA gyrase B inhibitors, topoisomerase II inhibitors

## Abstract

Coumarins are a privileged scaffold in medicinal chemistry, renowned for diverse therapeutic activities including antiviral, anticancer, and neuroprotective effects. Building on our previous work with 3‐substituted coumarins as inhibitors of tumor‐associated carbonic anhydrases, we report a novel series of thiazol‐hydrazono‐coumarins targeting the ATP‐binding domain of topoisomerase enzymes. Seventeen compounds were synthesized and evaluated for selective cytotoxicity against HeLa cells versus WI‐38 fibroblasts and for antimicrobial activity against four ESKAPE pathogens, *Escherichia coli* , and *Salmonella typhimurium* . Several derivatives showed potent antibacterial activity, with MICs as low as 0.12 μg/mL against resistant *Staphylococcus aureus* strains and inhibition zones up to 33 mm against Gram‐negative bacteria. Compound 13 exhibited strong selectivity, with an IC_50_ of 26.8 μg/mL in HeLa cells and 220.7 μg/mL in WI‐38 cells. The five most active compounds were studied via molecular docking and MM/GBSA to elucidate their binding to bacterial DNA gyrase, topoisomerase IV, and human topoisomerase IIα. A molecular dynamics simulation of the *S. aureus* DNA gyrase B‐compound 13 complex revealed a novel hydrogen bond between the coumarin ring and serine‐129. These findings highlight thiazol‐hydrazono‐coumarins as promising antibacterial leads with ancillary anticancer activity, supporting their potential in treating infections in immunocompromised cancer patients.

## Graphical abstract

Compound 13, a coumarin‐thiazole hybrid, is shown engaging the ATP‐binding site of *S. aureus* GyrB through a novel hydrogen bond with residue SER129. The molecule exhibits dual antibacterial and anticancer activity, supporting its potential as a dual‐target topoisomerase inhibitor.

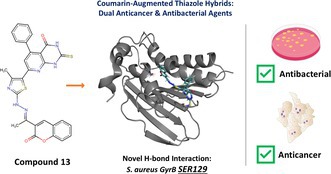

## 1 Introduction

**FIGURE 1:**
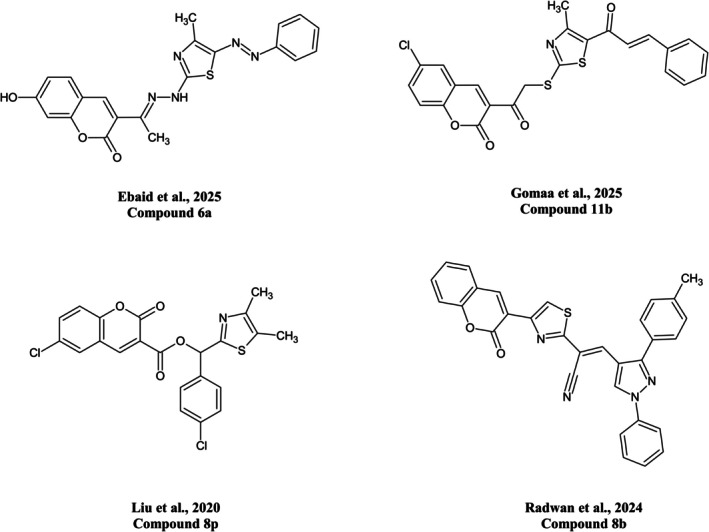
Representative literature‐reported coumarin‐thiazole hybrid scaffolds bearing hydrazone or enone linkers and ester/thioester connections, illustrating common structural diversification strategies used to tune the antimicrobial bioactivity of coumarins. The examples shown correspond to Ebaid et al. 2025 (compound 6a), Gomaa et al. 2025 (compound 11b), Liu et al. 2020 (compound 8p), and Radwan et al. 2024 (compound 8b).

Bacterial infections continue as a persistent cause of mortality in cancer patients. ESKAPE pathogens, namely *Enterococcus faecium* , *Staphylococcus aureus* , *Klebsiella pneumoniae* , *Acinetobacter baumannii* , *Pseudomonas aeruginosa* , and *Enterobacter* spp., are conspicuously infamous for nosocomial infections in oncology wards (Ma et al. 2020; Miller and Arias 2024; Mulani et al. 2019; Nanayakkara et al. 2021; Zhen et al. 2019). Lead compounds with dual activity as anticancer and antimicrobial agents are a possible strategy to address this lethal co‐morbidity. Coumarins are well known for their polypharmacological propensities, with a chemotherapeutic activity spectrum spanning multiple pharmacological spaces, including both anticancer and antimicrobial spaces (Abdulrehman et al. 2024; Basappa et al. 2020; Curini et al. 2003; Emami et al. 2008; Guillemin et al. 1986; Manvar et al. 2011; Ostrov et al. 2007; Patel et al. 2021; Qu et al. 2020; Reen et al. 2018; Sairam et al. 2016; Wahab et al. 2024; Yildirim et al. 2023; Zhang et al. 2011). For instance, derivatives of 3,3′‐(3,4‐dichlorobenzylidene)‐bis‐(4‐hydroxycoumarin), known as DCH, are separately patented for their activity against multidrug‐resistant *Staphylococcus aureus* and *Lyssavirus rabies*, the rabies virus (China Patent No. CN108635346A, 2018; China Patent No. CN103333148A 李明凯 2013). Historically, the antimicrobial activity of coumarins was first reported by Andres Goth in 1945 on the natural anticoagulant dicumarol (Goth 1945; Rehman et al. 2010). A decade later, aminocoumarin antibiotics were introduced, with streptonivicin (currently known as novobiocin) as the first of their class, in 1955, and coumermycin A_1_ as the second, in 1969 (Hoeksema et al. 1955; Michaeli et al. 1969). Today, there are 13 experimental protein data bank (PDB) structures with novobiocin as the subject of investigation, and 2 featuring coumermycin A_1_ (Coumermycin A1, n.d.; Novobiocin, n.d.). Additionally, recent medicinal chemistry efforts have increasingly explored hybrid coumarin architectures, where the coumarin nucleus is covalently linked to heteroatom‐rich motifs and conjugated linkers to tune target engagement. Representative examples from recent studies are shown in Figure 1, illustrating how diversification of the coumarin scaffold (e.g., thiazole‐ or azole‐containing appendages, hydrazone or enone linkers, and ester/thioester connections) expands the accessible chemical space while retaining the privileged coumarin pharmacophore. Collectively, these precedents support continued exploration of functionalized coumarins as antibacterial agents (Ebaid et al. 2025; Gomaa et al. 2025; Liu et al. 2020; Radwan et al. 2024).

Mechanistically, the antimicrobial action of coumarins is mediated through the inhibition of the bacterial DNA gyrase and topoisomerase IV enzymes, both of which are essential for negative supercoiling during DNA replication and transcription (Gellert et al. 1976; Maxwell 1993; Sugino et al. 1978). The bacterial DNA gyrase is a tetrameric protein comprising a dimer of two subunits, GyrA and GyrB. The prokaryotic topoisomerase IV is structurally homologous to the gyrase enzyme, with subunits ParC and ParE equivalent to GyrA and GyrB, respectively. Coumarins competitively bind the ATPase domain of GyrB and ParE, which deprives the DNA gyrase and topoisomerase IV, respectively, of the energy needed to introduce negative supercoils to the bacterial DNA. Correspondingly, coumarins act as anticancer agents by interfering with the eukaryotic topoisomerase IIα activity, thereby suppressing the rapid proliferation of cancer cells.

The DNA double‐helical structure necessitates constant topological regulation during replication, transcription, and recombination (Watson and Crick 1953). Topoisomerases resolve this by introducing transient DNA breaks to regulate supercoiling and untangle chromosomes (Bush et al. 2015). Hence, topoisomerases have been extensively exploited as (antimicrobial) chemotherapeutic drug targets. Both DNA gyrase and topoisomerase IV are bacterial type II topoisomerases. DNA gyrase is unique among topoisomerases in its ability to introduce negative supercoils into the bacterial chromosome, a feature critical for relieving positive superhelical tension ahead of replisomes and transcription complexes (Rajakumari et al. 2024; Reece and Maxwell 1991). In contrast, topoisomerase IV specializes in decatenation—resolving interlinked daughter chromosomes at replication termination—and also contributes to the relaxation of positive supercoils (Adams et al. 1992; Kato et al. 1990). Both enzymes harness ATP hydrolysis via their B‐subunits (GyrB or ParE) to catalyze strand passage through transient double‐strand breaks, yet each enzyme is specialized for a distinct physiological role: gyrase predominantly regulates supercoiling, while topoisomerase IV ensures chromosome segregation fidelity (Azam et al. 2015; Contreras and Maxwell 1992; Flatman et al. 2006; Reece and Maxwell 1991). Coumarin‐class inhibitors, including novobiocin, clorobiocin, and coumermycins, target the ATP‐binding pocket of GyrB (and to a lesser extent ParE), acting as competitive or conformation‐stabilizing inhibitors that disrupt the N‐terminal ATPase domain (Azam et al. 2015; Collin et al. 2011; Contreras and Maxwell 1992; Vanden Broeck et al. 2019). High‐resolution structures confirm their overlap with the ATP‐binding site: the noviose sugar of coumarins occupies the position of the adenine moiety of ATP, preventing nucleotide binding and hydrolysis (Collin et al. 2011; Lafitte et al. 2002). Functionally, this inhibition halts negative supercoiling by DNA gyrase, which induces replication fork stalling, impaired transcription, and ultimately bacterial cell death (Collin et al. 2011; Hardy and Cozzarelli 2003). When topoisomerase IV is similarly inhibited, chromosome decatenation is compromised, leading to segregation defects and anucleate cell formation (Adams et al. 1992; Hayama and Marians 2010; Hooper and Jacoby 2016). The dual targeting of DNA gyrase and topoisomerase IV thus provides a synergistic blocking of both replication progression and post‐replicative chromosome resolution, thereby bypassing potential drug resistance driven by mutations in either target (Hardy and Cozzarelli 2003).

By comparison, human topoisomerase IIα is a homodimeric type IIA enzyme that shares mechanistic similarity to bacterial DNA gyrase and topoisomerase IV in enabling strand passage via ATP‐hydrolysis and transient double‐strand cleavage (McKie et al. 2021; Pommier et al. 2010; Skok et al. 2020). All three enzymes share a conserved architecture: an N‐terminal GHKL ATPase, a central DNA‐gate, and a C‐terminal domain. Despite this shared three‐domain fold and ATP‐driven strand‐passage mechanism, topoisomerase IIα lacks the distinct subunit specialization of DNA gyrase and topoisomerase IV, and its homodimeric form integrates these functions into a single polypeptide capable of mitotic chromosome segregation and supercoil resolution in eukaryotes (Lynch et al. 1997; Nielsen et al. 2020; Pommier et al. 2022; Vanden Broeck et al. 2021; Wendorff et al. 2012). Clinically successful topoisomerase IIα inhibitors, such as etoposide or doxorubicin, act as poisons through a mechanism that stabilizes the enzyme cleavage complexes, generating cytotoxic DNA breaks that accumulate and eventually lead to cell death (Pommier et al. 2010; Skok et al. 2020).

On the synthetic front, chalcones are well‐established precursors for the construction of numerous heterocycles via enone cyclization, enabling access to rigid heterocyclic frameworks with broad biological activities (Nepali et al. 2014). Cyclization of chalcones affords nitrogen‐bearing heterocycles such as pyrazolines, (Insuasty et al. 2013) pyrimidines, (Nehra et al. 2020), and fused pyrimidines (Abdelhamid et al. 2010). Many pyrazoline derivatives have demonstrated potent pharmacological activities across diverse therapeutic areas (Shekhar Yadav et al. 2024), while pyrimidines and their congeners have gained prominence due to their considerable pharmaceutical value (Jain et al. 2016; Wang et al. 2016). Building on our recent findings that coumarin‐augmented thiazolyl chalcones exhibit marked anticancer activity against HeLa cervical cancer cells (Matar et al. 2024) together with the antibacterial findings shown in Figure 1, we envisioned these hybrid systems as versatile cycloaddition scaffolds for the efficient synthesis of novel five‐ and six‐membered heterocyclic derivatives, such as pyrazolines, pyrimidines, and fused pyrimidines, with potentially enhanced antimicrobial and anticancer properties.

Guided by these considerations, we synthesized 17 novel thiazol‐hydrazono‐coumarin derivatives and evaluated their anticancer activity against HeLa cells alongside antimicrobial activity against six bacterial pathogens. The five most active compounds (11, 13, 17c, 17e, and 18b) were further investigated using molecular docking and MM/GBSA binding affinity calculations. Additionally, molecular dynamics simulations of the lead compound, 13, in complex with the GyrB subunit of *Staphylococcus aureus* DNA gyrase (DGb) were performed over 100 ns, revealing an unprecedented (to our knowledge) hydrogen bond between the lactone moiety of the coumarin ring and Ser129 of DGb. To further assess the drug‐likeness of compound 13, we predicted its pharmacokinetic (ADMET) properties using SwissADME and ProTox 3.0. Taken together, these findings nominate compound 13 as a promising dual‐acting antimicrobial and anticancer lead candidate.

## 2 Results and Discussion

### 2.1 Chemistry

**SCHEME 1:**
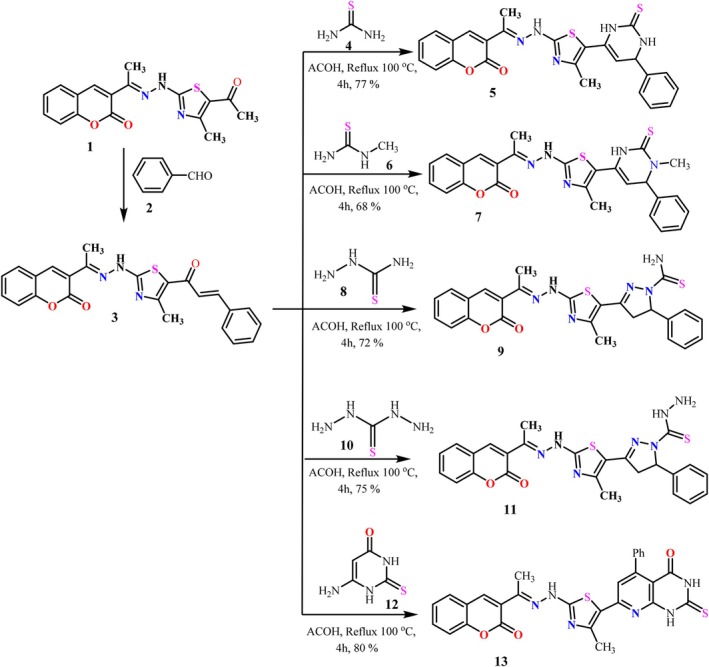
Synthesis of arylazothiazole derivatives **5, 7, 9, 11, 13**.

To kickstart our synthetic protocol, coumarin‐augmented thiazolyl chalcone **3** was chosen as a pivotal scaffold to easily access a host of five‐ and six‐membered heterocyclic systems with potential biological activity. Pyrimidine‐2‐thione derivatives **5** and **7** were obtained upon its reaction with thiourea **4** and *N‐*methyl thiourea **5**, respectively, via [3 + 3] cycloaddition as sketched in Scheme 1. IR spectrum of the obtained pyrimidine‐2‐thione derivative **5** showed absorption bands of the thiocarbonyl group (C=S) at 1134 cm^−1^ and of the stretching vibrations of the three NH bonds in the carbamate and amide groups at 3425, 3325, and 3280 cm^−1^, respectively. In the ^1^Н NMR spectrum of compound **5**, Н‐5 and Н‐6 protons of the pyrimidinethione moiety appear as a pair of doublets at 4.72 and 6.43 ppm, respectively, and the absorption of the NH groups is observed at 8.42 and 11.50 ppm, indicating the presence of the C=S group as the dominant tautomer (Scheme 1).

Additionally, condensation of chalcone **3** with thiosemicarbazide **8** and thiocarbohydrazide **10** in refluxing glacial acetic acid led to the formation of the corresponding pyrazoline derivatives **9** and **11**, respectively, via [3 + 2] cycloaddition as shown in Scheme 1. IR spectrum of the obtained pyrazoline derivative **9** showed absorption bands of the thiocarbonyl (C=S) group at 1056 cm^−1^ and two stretching vibrations of the NH_2_ group at 3325 and 3255 cm^−1^. In its ^1^Н NMR spectrum, pyrazoline derivative **9** showed a pair of doublets at *δ* = 3.09–3.27 and 3.42–3.76 ppm for the pyrazoline CH_2_, a doublet at 4.65–4.77 ppm for the pyrazoline CH‐5, and a broad singlet at 6.88 ppm for the NH_2_ group.

Furthermore, cycloaddition of chalcone **3** with 6‐aminothiouracil **12** in boiling acetic acid led to the formation of the corresponding pyridopyrimidinethione derivative **13**. The reaction is presumably initiated by Michael‐type addition followed by cyclization with elimination of a water molecule and aromatization as depicted in Scheme 1. IR spectrum of the obtained pyridopyrimidinethione derivative **13** showed absorption bands at ν 3421–3288 (3NH), 1650, 1688 (C=O), and 1150–1580 cm^−1^ (C=S). In its ^1^Н NMR spectrum, compound **13** showed three singlets at *δ* 7.40, 9.87, and 11.98 for the pyridine‐H and the two NH groups, respectively.

It is worth mentioning that the conventional base‐catalyzed Claisen‐Schmidt condensation of 3‐acetylthiazole derivative **1** with benzaldehyde in a sodium hydroxide ethanolic blend (NaOH/EtOH) afforded chalcone derivative **3** in excellent yield, as we previously reported (Matar et al. 2024).

**SCHEME 2:**
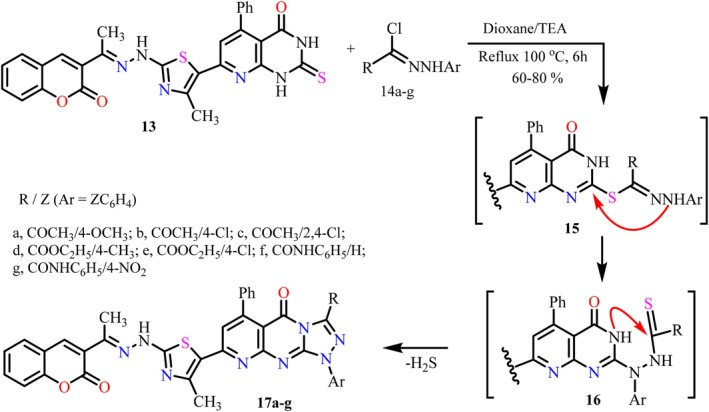
Synthesis of triazolopyrimidines **17a‐g**.

To exploit the potential versatility of the freshly mounted thione handle to achieve heteroannulation, the pyridopyrimidinethione derivative **13** was treated with a group of hydrazonoyl chlorides **14a‐g** under basic (Et_3_N) conditions to afford a new series of pyridotriazolopyrimidines **17a‐g** very smoothly, as demonstrated in Scheme 2. The ^1^Н NMR spectrum of triazolopyrimidine derivative **17a** showed all distinctive signals of all nonequivalent protons, including a pair of singlets corresponding to the two new methyl groups of COCH_3_ and ArOCH_3_ on the triazole moiety, appearing at 2.46 and 3.64 ppm.

Mechanistically, the reaction is presumably initiated by a base‐catalyzed *S*‐alkylation of the thione handle, followed by Smiles rearrangement of intermediate **15** and ultimately cyclization of intermediate **16** with immediate elimination of an H_2_S molecule to give the proposed triazolopyrimidine derivative **17** as portrayed in Scheme 2.

**SCHEME 3:**
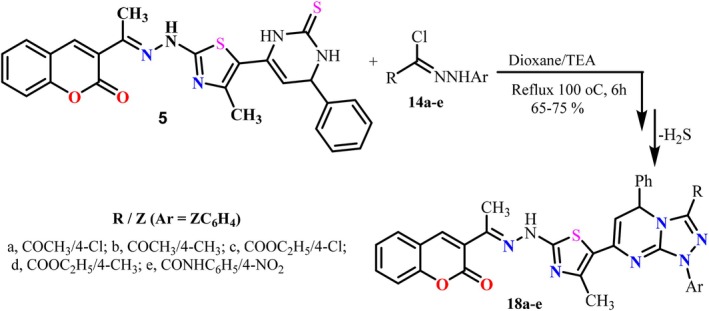
Synthesis of triazolopyridopyrimidines **18a‐e**.

Similarly, the versatility of the freshly mounted thione handle was exploited to achieve heteroannulation of the pyrimidinethione derivative **5** upon treatment with a group of hydrazonoyl chlorides **14a‐g** under basic (Et_3_N) conditions to afford a new group of triazolopyrimidine derivatives **18a‐g** (Scheme 3). Extensive structural elucidation was based on both spectral and elemental analyses, as detailed in the experimental section.

### 2.2 Biological Evaluation

#### 2.2.1 In Vitro Cytotoxic Activity Against HeLa Cells

**FIGURE 2:**
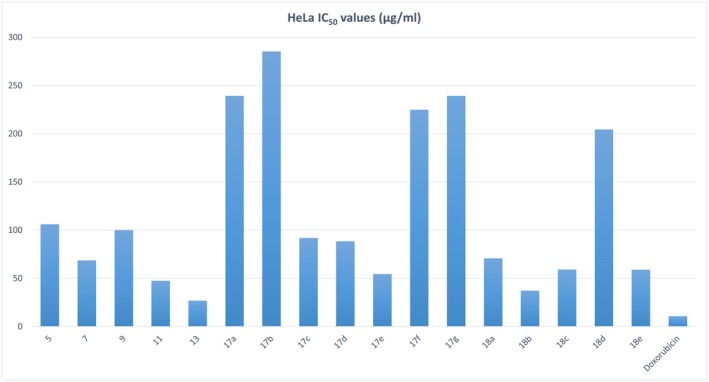
IC_50_ values (μg/mL) of all synthesized coumarin derivatives against HeLa cervical cancer cells. Compound 13 exhibited one of the lowest IC_50_ values among the tested compounds, indicating notable cytotoxic activity. Doxorubicin was included as a reference control.

The cytotoxic potential of the newly synthesized thiazol‐hydrazono‐coumarin derivatives was assessed against HeLa cervical cancer cells using the MTT colorimetric assay (Report S2). All tested compounds exhibited concentration‐dependent inhibition of cell viability, with IC_50_ values ranging from 26.8 to 285.2 μg/mL (Figure 2, Table S1). Among them, compound 13 displayed the most potent activity, with an IC_50_ of 26.8 ± 0.97 μg/mL, followed by compounds 18b (37.0 ± 2.13 μg/mL), 11 (47.5 ± 2.13 μg/mL), and 17e (54.4 ± 3.06 μg/mL). These results positioned compound 13 as the lead candidate for further evaluation.

By comparison, compounds 5 and 9 showed relatively weak cytotoxicity with IC_50_ values exceeding 90 μg/mL. The reference drug doxorubicin exhibited an IC_50_ of 10.59 ± 1.03 μg/mL under the same conditions. While none of the tested coumarins surpassed doxorubicin in potency, the favorable selectivity indices of compounds like 13 and 18b (discussed in Section 2.2.4) suggest therapeutic promise as lead anticancer scaffolds.

#### 2.2.2 Antibacterial Activity Against *Staphylococcus aureus* Strains

**FIGURE 3:**
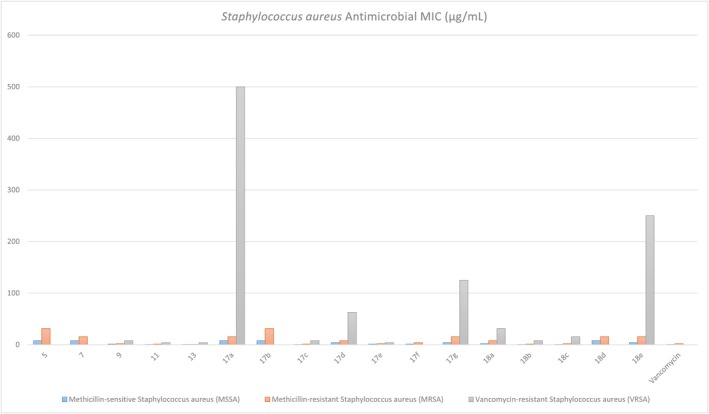
Minimum inhibitory concentrations (MICs, μg/mL) of the synthesized coumarin derivatives against *Staphylococcus aureus* strains, including methicillin‐sensitive (MSSA), methicillin‐resistant (MRSA), and vancomycin‐resistant (VRSA) isolates. Compounds 11, 13, 17c, 17e, and 18b displayed potent activity across all three strains, while some derivatives showed markedly reduced efficacy against VRSA. Vancomycin was included as a positive control.

The antibacterial activity of the synthesized coumarin derivatives was evaluated against three clinically relevant *Staphylococcus aureus* strains: methicillin‐sensitive (MSSA, ATCC 29213), methicillin‐resistant (MRSA, ATCC 700788), and vancomycin‐resistant (VRSA, RCMB 28354). Minimum inhibitory concentrations (MICs) were determined using the broth microdilution method with XTT as a viability indicator (Table S2, Figure 3).

Compounds 11, 13, 17c, 17e, and 18b demonstrated strong and broad‐spectrum anti‐Staphylococcal activity across all three strains. Notably, compound 13 exhibited the most potent activity overall, with MIC values of 0.12 μg/mL (MSSA), 0.48 μg/mL (MRSA), and 3.9 μg/mL (VRSA), outperforming vancomycin in both MSSA and MRSA assays. Compound 11 showed nearly comparable efficacy, with MICs of 0.24, 0.98, and 3.9 μg/mL against MSSA, MRSA, and VRSA, respectively.

Among the top‐performing analogues, compound 17c exhibited MICs of 0.24–7.81 μg/mL across the strains, while 17e and 18b maintained VRSA activity below 8 μg/mL. Other compounds, including 5, 7, and 17b, displayed moderate activity with MIC values ranging from 7.81 to 31.25 μg/mL against MRSA and MSSA, but were generally ineffective against VRSA.

These findings underscore compound 13's superior antibacterial potency and broad spectrum across drug‐sensitive and multidrug‐resistant *S. aureus* strains, highlighting its potential as a dual‐action lead candidate.

#### 2.2.3 Antibacterial Activity Against Gram‐Negative Pathogens

**FIGURE 4:**
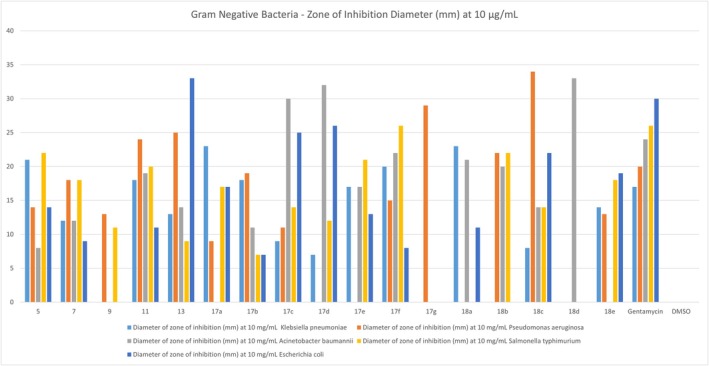
Antibacterial activity of the novel coumarin derivatives against Gram‐negative pathogens, expressed as zone of inhibition diameters (mm) at 10 μg/mL. Tested organisms include *Klebsiella pneumoniae* , *Pseudomonas aeruginosa* , *Acinetobacter baumannii* , *Escherichia coli* , and *Salmonella typhimurium* . Several compounds, most notably 13, 17c, and 18c, exhibited broad‐spectrum activity with inhibition zones exceeding 25 mm against some strains. Gentamycin was used as a positive control; DMSO served as a negative control.

The antibacterial potential of the synthesized coumarin derivatives was further evaluated against five clinically relevant Gram‐negative bacterial strains: *Klebsiella pneumoniae* , *Pseudomonas aeruginosa* , *Acinetobacter baumannii* , *Salmonella typhimurium* , and *Escherichia coli* . The agar well diffusion method was used at a fixed concentration of 10 μg/mL, and antimicrobial activity was quantified by measuring the diameter of the inhibition zone (Figure 4, Table S3).

Several compounds demonstrated broad‐spectrum Gram‐negative activity, most notably compound 13, which exhibited inhibition zones of 25 mm against *P. aeruginosa* , *A. baumannii* (14 mm), *K. pneumoniae* (13 mm), and *E. coli* (33 mm). Compound 11 also showed strong inhibition against four of the five strains, including *K. pneumoniae* (18 mm) and *P. aeruginosa* (24 mm). Compound 18c displayed similarly broad activity, with zones ranging from 8 to 34 mm. On the other hand, compounds such as 7 and 9 exhibited weaker antibacterial effects, with inhibition zones below 15 mm against most tested strains.

The high potency and broad‐spectrum profile of compound 13 against both Gram‐positive and Gram‐negative bacteria reinforce its status as a lead candidate with potential utility in treating mixed or opportunistic infections in immunocompromised cancer patients.

#### 2.2.4 Cytotoxicity Against Normal Human Lung Fibroblasts (WI‐38)

**FIGURE 5:**
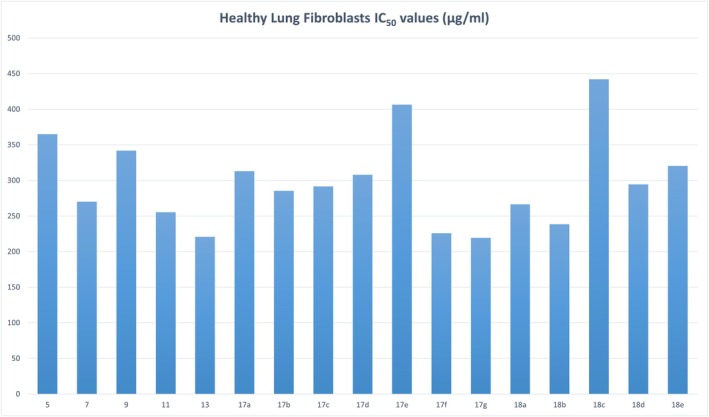
IC_50_ values (μg/mL) of the new coumarin derivatives against healthy human lung fibroblast (WI‐38) cells. The data highlight the comparatively low cytotoxicity of the compounds toward non‐cancerous cells, with most IC_50_ values exceeding 250 μg/mL.

To evaluate the selectivity and potential safety of the synthesized compounds, their cytotoxicity was assessed against WI‐38 normal human lung fibroblast cells using the MTT assay. The majority of compounds exhibited minimal cytotoxic effects toward non‐cancerous cells, with IC_50_ values exceeding 250 μg/mL (Figure 5, Table S4). Compound 18c was the least cytotoxic overall, with an IC_50_ of 442.0 ± 4.0 μg/mL, followed by compounds 5, 9, and 17e, all with IC_50_ values above 330 μg/mL.

Notably, compound 13, which demonstrated the strongest activity against HeLa cells (IC_50_ = 26.8 ± 0.97 μg/mL), also showed comparatively low toxicity in WI‐38 cells (IC_50_ = 220.7 ± 4.0 μg/mL), yielding a selectivity index (SI) of 8.24. This favorable SI highlights compound 13 as a promising lead anticancer candidate with a potentially safe therapeutic window. A similar selectivity profile was observed for compounds 11, 17e, and 18b, reinforcing the relevance of these molecules for further biological investigation.

### 2.3 Molecular Modeling

#### 2.3.1 Chemical Similarity Analysis

**FIGURE 6:**
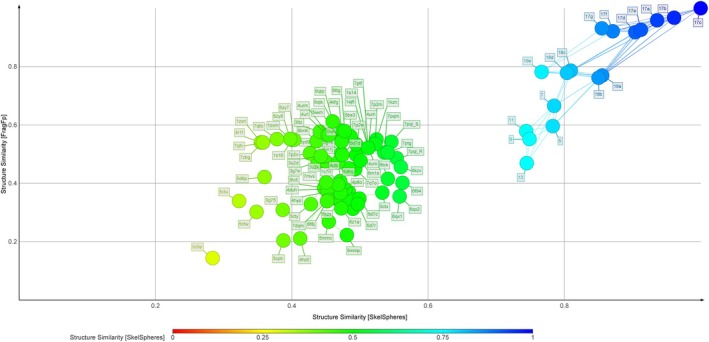
Two‐dimensional similarity map of the 95‐compound dataset based on two molecular fingerprints (FragFp and SkelSpheres), computed in DataWarrior, with compound 17c used as the reference structure. The *x*‐axis represents pairwise structural similarity according to the SkelSpheres descriptor, while the *y*‐axis corresponds to similarity based on FragFp. Circle color intensity reflects SkelSpheres similarity values, from red (low) to blue (high). The novel coumarin derivatives (including compounds 11, 13, 17c, 17e, and 18b) form a distinct cluster on the right in various shades of blue, clearly separated from the co‐crystallized reference ligands on the left. Interconnecting lines indicate high‐similarity edges based on SkelSpheres mapping.

**FIGURE 7:**
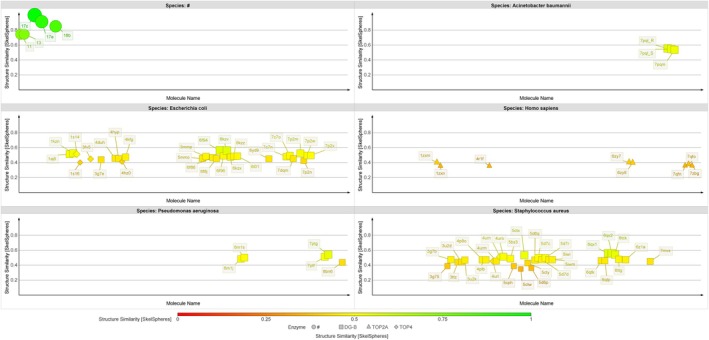
Structure similarity distribution of the 95‐compound dataset across five species, based on the SkelSpheres descriptor in DataWarrior. Each subplot depicts individual compounds color‐coded by SkelSpheres similarity (from red = low to green = high) and grouped by species: *Acinetobacter baumannii* , *Escherichia coli* , *Homo sapiens* , *Pseudomonas aeruginosa* , and *Staphylococcus aureus* . The top‐left subplot (labeled “#”) displays the prioritized novel coumarin compounds 11, 13, 17c, 17e, and 18b. Marker shapes represent enzyme class: squares for DNA gyrase B (DG‐B), triangles for topoisomerase II‐alpha (TOP2A), and diamonds for topoisomerase IV (TOP4). Coumarins form a distinct, highly similar cluster (green, top‐left), whereas co‐crystallized ligands from other species exhibit broader structural diversity (yellow to red).

The 17 novel coumarin derivatives were analyzed alongside 58 co‐crystallized ligands from the final curated topoisomerase crystal structures, as well as 20 additional ligands from excluded complexes, to evaluate the Tanimoto similarity landscape of the dataset (Tanimoto 1958; Willett et al. 1998). Chemical space projections were constructed using two complementary approaches: a two‐dimensional (2D) map derived from DataWarrior's FragFp and SkelSpheres fingerprint descriptors (Figure 6), and a three‐dimensional (3D) map incorporating the Flexophore descriptor to capture pharmacophoric alignment (Figure S1). To facilitate cross‐species comparisons and enable targeted structure selection, the 2D map was further segmented into six categorical panels based on the species of origin for each ligand (Figure 7).

The 2D similarity map revealed that the novel coumarins formed a distinct and structurally homogeneous cluster, clearly separated from the more chemically diverse co‐crystallized reference ligands. This observation supported the hypothesis that the coumarin series would exhibit a relatively consistent binding behavior within each topoisomerase enzyme. Accordingly, this internal similarity guided the curation of their docking poses, with the expectation of recurring pose patterns when each coumarin was docked into the same target. Additionally, the separation of the query coumarins from known ligands suggested a potential divergence in binding modes, which influenced the decision to perform docking without enforcing template‐based constraints. This ensured that structurally novel binding poses were not penalized or excluded a priori, a rationale later substantiated by results such as compound 13 adopting a previously unreported deep‐pocket interaction in the *S. aureus* GyrB binding site.

Chemical similarity analysis also played a direct role in guiding the selection of docking targets. The SkelSpheres‐based map enabled the identification of co‐crystallized ligands most similar to the prioritized coumarins within each species. This informed the selection of crystal structures likely to present relevant binding site conformations. Once candidate structures were identified based on similarity, further refinement was performed by assessing crystal quality, resolution, and co‐crystallized ligand properties. While the 3D Flexophore map (Figure S1) did not reveal additional separation beyond what was seen in 2D, it served as a useful confirmatory tool to visualize pharmacophoric consistency.

#### 2.3.2 Target Alignment and Pharmacophoric Residue Conservation

**FIGURE 8:**
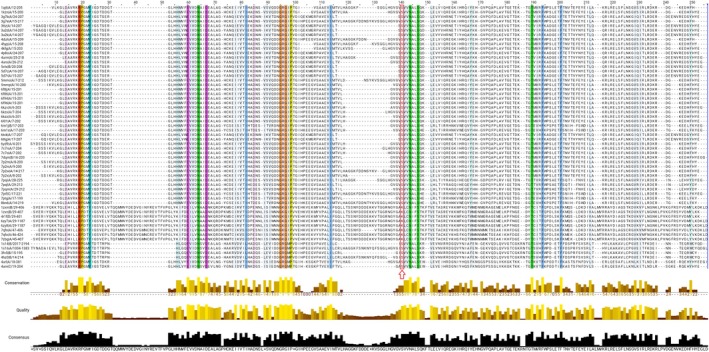
Multiple sequence alignment of selected topoisomerase enzymes, highlighting conserved residues across four bacterial species and the human homolog. The alignment includes the GyrB and ParE domains from bacterial DNA gyrase and topoisomerase IV, and the full‐length (visually truncated) human topoisomerase IIα. Residues are colored by conservation using Clustal's standard scheme, with ≥ 80% conserved residues highlighted. The histogram plots below the alignment indicate residue‐wise conservation, alignment quality, and consensus. Notably, the conserved SVV motif (indicated by a red arrow) includes a serine residue critical to binding site architecture in bacterial homologs but less conserved in the human enzyme. This differential conservation supports the rationale for designing ligands with weighted targeting.

Jalview was used to synchronously trace sequence and structural conservation within the curated topoisomerase dataset and to identify potentially overlapping pharmacophoric regions. The multiple sequence alignment included the GyrB and ParE domains from the selected bacterial DNA gyrase and topoisomerase IV structures, as well as the full‐length human topoisomerase IIα sequence. Conservation scores were computed using Jalview's default parameters and Clustal's color scheme, with residues showing ≥ 80% conservation highlighted for downstream interpretation (Figure 8).

**FIGURE 9:**
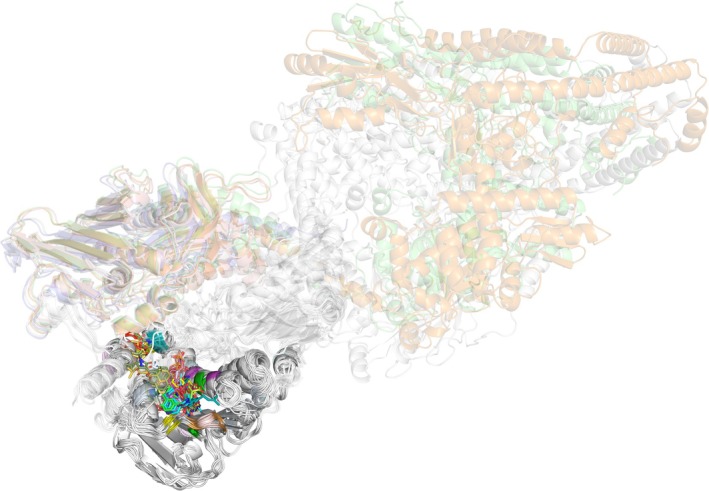
Structural alignment of the curated topoisomerase enzymes from four bacterial species and the human homolog, visualized in PyMOL. Structures were aligned using Jalview's integrated structure viewer, focusing on the ATPase domains (GyrB and ParE) for bacterial DNA gyrase and topoisomerase IV, and the full‐length human topoisomerase IIα. The cartoon representation highlights the conserved core fold shared across the three enzyme classes. Ligands from co‐crystallized complexes are shown as sticks, illustrating the convergence of binding pockets despite species‐specific structural variation. The human protein's non‐aligned regions are shown as semi‐transparent to enhance clarity.

Candidate binding site residues (rendered as sticks) were initially defined based on a previously published ligand‐bound complex analysis (Figure S2) (Elseginy and Anwar 2022). Despite the highly conserved overall fold of the ATPase domain across the three enzyme classes (Figure 9), several ligand‐contacting residues displayed moderate to high conservation among bacterial homologs and lower conservation in the human counterpart. Notably, the serine residue within the SVV motif, located deep within the catalytic cavity and highlighted in Figure 8, was one such example of bacterial‐specific conservation. This divergence among the binding site residues reinforces the potential to rationally design ligands with tailored selectivity toward bacterial versus human targets, thereby enabling a (poly)pharmacological strategy with weighted therapeutic advantages.

#### 2.3.3 Evaluating Disorder in Target Complexes

**FIGURE 10:**
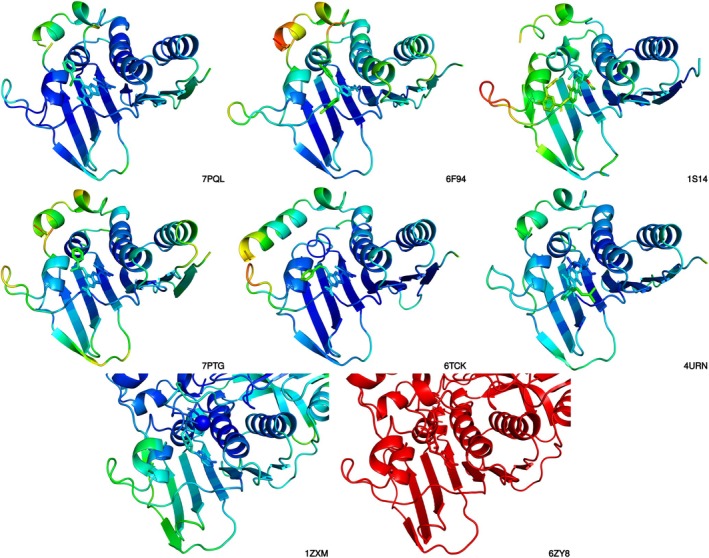
Visualization of per‐residue B‐factors in the ATP‐binding domains of the eight selected topoisomerase crystal structures. Structures are colored from blue (low B‐factor, well‐ordered) to red (high B‐factor, disordered). The human topoisomerase IIα structure 6ZY8 (bottom right) exhibited pronounced disorder in its ATPase domain and was excluded from molecular docking. Elevated B‐factor regions in other targets (e.g., 1S14) were noted for their potential influence on docking pose reproducibility.

Many of the co‐crystallized ligands in the curated topoisomerase dataset were partially solvent‐exposed, prompting a quality control step to evaluate structural uncertainty within the binding sites of the selected target complexes (PDB IDs: 7PQL, 6F94, 1S14, 1ZXM, 6ZY8, 7PTG, 6TCK, and 4URN). To assess the local uncertainty in atomic coordinates, B‐factors were visualized using PyMOL (Figure 10). This analysis revealed significant disorder in the ATPase domain of the human topoisomerase IIα crystal structure 6ZY8, leading to its exclusion from subsequent docking studies. Furthermore, elevated B‐factors observed in other targets (e.g., 1S14) were noted as potential contributors to increased RMSD values during redocking validation (Docking and Scoring 2021; Halip et al. 2021).

#### 2.3.4 Molecular Docking and Binding Affinity Estimation

Molecular docking was performed for the five prioritized thiazol‐hydrazono‐coumarin derivatives (compounds 11, 13, 17c, 17e, and 18b) across seven curated crystal structures representing the ATP‐binding pockets of DNA gyrase subunit B (GyrB) and topoisomerase IV subunit B (ParE) from *A. baumannii* (7PQL), *E. coli* (6F94, 1S14), *P. aeruginosa* (7PTG), and *S. aureus* (6TCK, 4URN), along with human topoisomerase IIα (1ZXM). The docking protocol was validated by redocking the native co‐crystallized ligands into their respective proteins, yielding RMSD values mostly below 3 Å (Table 1). The largest deviation was observed for the *E. coli* topoisomerase IV structure (1S14), where visual inspection indicated that the deviation was mainly localized to the solvent‐exposed tail region of the ligand (Figure S3); an area known for high flexibility and elevated B‐factor values (Docking and Scoring 2021). These observations highlighted the limitations of RMSD as a standalone metric for pose validation in these partially disordered binding sites. Accordingly, final docking poses were curated based on a combination of consensus across top‐ranked poses and docking scores, thereby improving confidence in the resulting binding hypotheses (Bouvier et al. 2010; Makeneni et al. 2018; Wei et al. 2013).

**TABLE 1:** Redocking RMSD values.

Enzyme PDB ID	RMSD
7PQL	2.159
6F94	1.280
1S14	3.890
7PTG	0.928
6TCK	0.953
4URN	2.992
1ZXM	2.751

**FIGURE 11:**
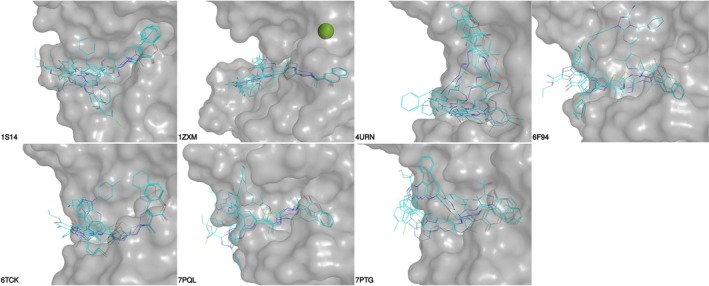
Predicted binding poses of the five prioritized coumarin derivatives (11, 13, 17c, 17e, and 18b) docked into the ATP‐binding clefts of seven topoisomerase targets. Surface representations of the protein pockets are shown for each target, with overlaid ligand poses rendered as stick models. Compounds consistently adopt a conserved binding mode across bacterial enzymes, anchoring the coumarin scaffold deep in the cleft and projecting the thiazol‐hydrazono substituents toward the solvent‐accessible pocket entrance. Notable pose deviations occur in *S. aureus* ParE (4URN), where greater variability is observed, and in the human Topo IIα model (1ZXM—Catalytic magnesium depicted as green sphere), where ligands shift to accommodate subtle differences in pocket shape.

**FIGURE 12:**
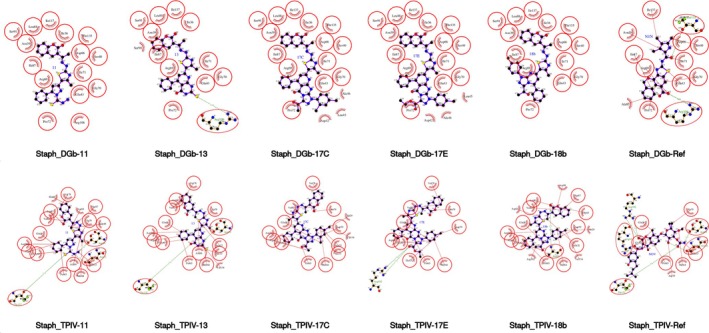
Two‐dimensional protein‐ligand interaction diagrams for the five lead coumarin derivatives and the reference ligand docked into *Staphylococcus aureus* DNA gyrase B (top row) and topoisomerase IV ParE (bottom row). Key interactions, including hydrogen bonds, hydrophobic contacts, and salt bridges, are annotated for each complex. The coumarin core consistently engages in π‐stacking and hydrogen bonding within the ATP‐binding pocket, while the thiazol‐hydrazono substituents extend toward the pocket entrance, forming additional contacts with polar residues. Notably, compound 13 shows a unique interaction with Arg106 in GyrB, absent in other derivatives, while compound 17e in ParE engages in extended polar interactions near the solvent‐accessible region.

Docking poses for the coumarin derivatives revealed consistent binding geometries within the ATP‐binding clefts across the bacterial enzymes (Figure 11). The coumarin core was frequently positioned deep within the pocket, except in the *S. aureus* ParE model, stabilized by hydrophobic contacts with conserved residues and hydrogen bonds involving the coumarin ring carbonyl and the imine nitrogen of the hydrazono linker. Substituents at the thiazol‐hydrazono end extended toward the solvent‐accessible entrance of the cleft, forming additional polar contacts with the flexible surface loop that includes the conserved GxGxP motif, as illustrated in the 2D interaction diagrams (Figure 12, Figure S4). While some variation in ligand orientation was observed across targets, the overall binding pose was broadly conserved and is consistent with binding modes reported for ATP‐competitive GyrB inhibitors.

**FIGURE 13:**
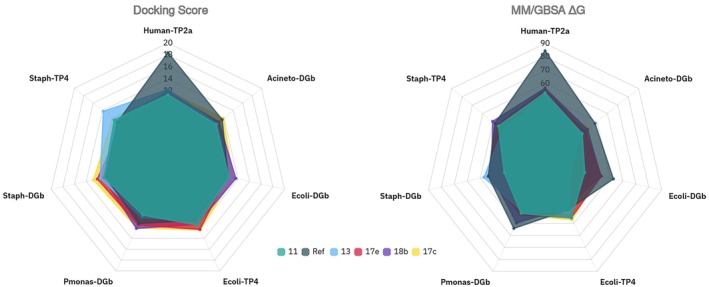
Comparative radar plots illustrating the predicted binding affinities of the five lead coumarin derivatives (11, 13, 17c, 17e, and 18b) across the seven topoisomerase ATP‐binding site models based on (A) Flare docking scores and (B) MM/GBSA ΔG values. Each axis represents a different enzyme target: six bacterial enzymes and one human topoisomerase IIα (1ZXM). Binding affinity signs have been inverted for visualization purposes. Both scoring methods consistently highlight compound 13 and 17c as high‐affinity ligands across multiple bacterial enzymes, particularly *S. aureus* GyrB (6TCK) and ParE (4URN), while compound 13 also shows strong predicted affinity for the human enzyme, suggesting a potential for tunable polypharmacology.

Among the tested compounds, 13 and 17c consistently exhibited the most favorable docking scores and MM/GBSA binding free energies across the bacterial targets (Figure 13, Table S5A,B, Figure S8A–G), correlating with their experimentally measured antimicrobial activities. For example, 17c ranked top in *A. baumannii* GyrB (−11.9) and *E. coli* ParE (−12.4), and yielded MM/GBSA energies as low as −47.1 kcal/mol. Compound 13 exhibited particularly favorable binding to the *S. aureus* targets, aligning with its potent in vitro anti‐staphylococcal activity. Compound 17e displayed moderate‐to‐good docking scores (−10 to −12.3) and variable MM/GBSA values, indicating potential for species‐specific optimization. Meanwhile, compound 18b, although lower scoring in some models, achieved top binding energies in *P. aeruginosa* GyrB and *S. aureus* ParE, indicating a possible niche binding profile for this compound.

Predicted binding affinities to human topoisomerase IIα (1ZXM) were also relatively high for all compounds, particularly compound 13 (docking score −12.3; MM/GBSA ΔG ≈ −51 kcal/mol), which aligns with its observed in vitro anticancer activity. Importantly, the substantially stronger binding affinity of the reference ligand, adenyl imidodiphosphate (AMP‐PNP), suggests a reasonable therapeutic window for the five lead coumarins, as further supported by their limited cytotoxicity against healthy human lung fibroblasts.

Overall, the integrated docking and MM/GBSA analysis supports a conserved binding mechanism among the coumarin derivatives and highlights lead compounds, especially 13 and 17c, as promising candidates for further development. These findings offer a rational foundation for scaffold optimization to achieve tunable selectivity between bacterial and human topoisomerases, supporting therapeutic strategies that leverage context‐specific polypharmacology. Moreover, although the ATP‐binding pockets of bacterial and human type IIA topoisomerases are structurally conserved, subtle differences in pocket geometry and residue composition (e.g., loop conformation and motif substitutions such as SVVNAL in bacteria versus KLCNIF in humans) may still be exploited to improve bacterial selectivity.

#### 2.3.5 Molecular Dynamics Simulation

**FIGURE 14:**
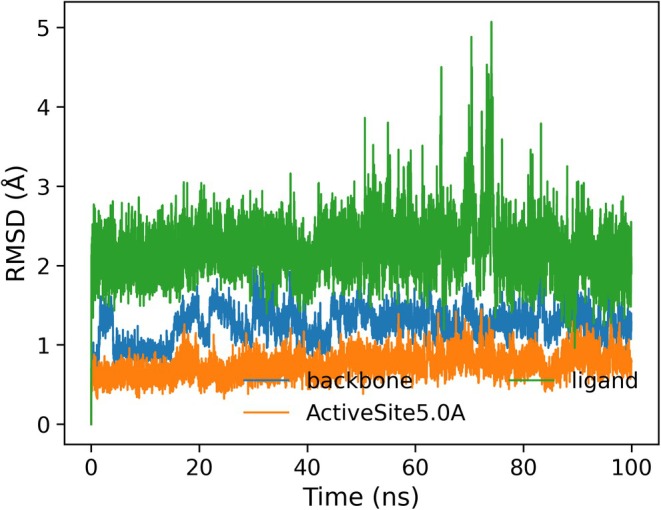
Root mean square deviation (RMSD) of the compound 13‐ *S. aureus* GyrB (6TCK) complex over a 100 ns molecular dynamics simulation. The protein backbone (blue) and active site residues within 5.0 Å of the ligand (orange) remained structurally stable throughout the trajectory, while the ligand (green) exhibited modest fluctuations consistent with retained binding and local adaptability.

To further investigate the stability and dynamic behavior of the complex of compound 13 with the *S. aureus* GyrB (PDB ID: 6TCK), a 100‐ns molecular dynamics (MD) simulation was performed. The system reached equilibrium rapidly, with the protein backbone RMSD stabilizing between 1.0 and 1.7 Å after the first 5 ns (Figure 14). The ligand RMSD remained within 2.0–2.8 Å throughout the simulation, indicating consistent retention within the ATP‐binding pocket. Root mean square fluctuation (RMSF) analysis revealed low mobility in the binding site region (typically < 1.5 Å), supporting a conformationally stable and rigid binding environment throughout the trajectory (Figure S5).

Significantly, the MD simulation revealed a new hydrogen bond between the coumarin lactone moiety of compound 13 and residue SER129, which was not present in the initial docking pose. This interaction represents a novel stabilization mechanism unique to compound 13, which has not been reported in the binding profiles of previous coumarin analogues. The hydrogen bond with SER129 was consistently observed throughout the trajectory, suggesting a dynamic rearrangement that enhances the enthalpic contribution to binding stability (Video S1). Further, additional persistent interactions (Figure S6) were observed with ARG144 and the backbone of GLY85, which flank the entrance of the ATP‐binding cleft. These residues engaged in frequent hydrogen bonding and polar contacts with the thiazol‐hydrazono substituent of compound 13, further anchoring the ligand. Finally, no significant loop rearrangements or water‐bridged interactions were observed during the simulation. The binding pocket maintained its compact and solvent‐shielded geometry, providing a consistent environment for ligand engagement. This was further supported by the radius of gyration analysis (Figure S7), which showed minimal fluctuation throughout the simulation, consistent with the overall protein's compactness being preserved upon ligand binding. Together, these MD‐derived insights reveal a refined and more stabilized binding mode, positioning compound 13 as a valuable scaffold with unique GyrB inhibition potential.

#### 2.3.6 Predicted ADMET Properties

The pharmacokinetic and toxicity profiles of compound 13 were evaluated in silico using SwissADME (Figure S9) and ProTox 3.0 (Figure S10A,B, Report S1). SwissADME predicted low gastrointestinal (GI) absorption and absence of blood–brain barrier (BBB) permeability, which may help minimize central nervous system‐related off‐target effects. The compound showed no predicted inhibitory interactions with major cytochrome P450 isoforms (CYP1A2, CYP2C9, CYP2D6, CYP3A4), suggesting a low risk of metabolic drug–drug interactions.

Compound 13 satisfied key drug‐likeness criteria, with only one violation of Lipinski's Rule of Five due to a molecular weight exceeding 500 Da. While no PAINS (pan‐assay interference compounds) alerts were detected, three Brenk structural alerts were identified, specifically for the coumarin scaffold, an imine moiety, and a thiocarbonyl group, warranting attention during lead optimization to reduce potential toxicity or instability.

ProTox 3.0 classified compound 13 as a toxicity class IV substance, with a predicted oral LD_50_ of 1000 mg/kg in rodents, indicating low acute toxicity. Among 15 evaluated toxicological endpoints, six were predicted to be active with moderate confidence: hepatotoxicity (probability 0.56), nephrotoxicity (0.55), respiratory toxicity (0.54), carcinogenicity (0.57), BBB‐barrier interference (0.62), and aryl hydrocarbon receptor activation (0.50). All remaining endpoints, including mutagenicity and cytotoxicity, were predicted to be inactive.

Overall, these ADMET predictions suggest that compound 13 possesses a reasonably favorable pharmacokinetic and safety profile, particularly in terms of metabolic compatibility and acute toxicity, while highlighting specific structural motifs that could be refined to mitigate long‐term toxicity risks during future lead optimization.

## 3 Materials and Methods

### 3.1 Chemistry

#### 3.1.1 Chemicals and Instruments

Instrumental information for all chemical and physical tools utilized to collect chemical, physical, and/or spectral data was collected in Table 2.

**TABLE 2:** Instrumental information for all chemical and physical tools utilized for collecting and recording chemical, physical, and spectral data.

Analysis	Instrument	Manufacturer
mp	Electrothermal IA 9000 series digital	UK
IR	FT‐IR‐4100 spectrophotometer (400–4000 cm^−1^)	USA
NMR	Bruker 400 MHz instrument	Japan
MS	Shimadzu GCMS‐QP 1000 EX mass spectrometer	Japan
Elemental	Elementar vario‐LIII C‐H‐N‐S analyzer	USA

#### 3.1.2 General Procedure for the Synthesis of Arylthiazole Derivatives 5, 7, 9, 11, 13

To a glacial acetic acid solution (15 mL) of chalcone derivative **3** (1 mmol), thiourea **4**, *N*‐methylthiourea **6**, thiosemicarbazide **8**, thiocarbohydrazide **10**, or amino thiouracil **12** (1 mmol) was added. The solution was refluxed for 4 h. Upon completion of the reaction, the resulting product was filtered out, washed with methanol, dried, and recrystallized from the proper solvent to give the corresponding arylthiazole derivatives **5, 7, 9, 11, or 13**, respectively.

##### 3.1.2.1 3‐(1‐(2‐(4‐Methyl‐5‐(6‐phenyl‐2‐thioxo‐1,2,3,6‐tetrahydropyrimidin‐4‐yl)thiazol‐2‐yl)hydrazono)ethyl)‐2H‐chromen‐2‐one (5)

Red solid; m.p. 124°C–125°C (EtOH); IR (KBr): *v* 3425, 3325, 3280 (3NH), 3070, 2916 (C‐H), 1651 (C=O) cm^−1^; ^1^H NMR (DMSO‐*d*_6_): *δ* 3.31 (s, 3H, CH_3_), 3.38 (s, 3H, CH_3_), 3.79 (s, 1H, CH‐CH=C), 6.66 (s, 1H, CH‐CH=C), 7.43–7.64 (m, 10H, Ar‐H, coumarin‐H), 8.54 (s, 1H, NH), 12.50 (br s, 2H, 2NH) ppm; ^13^C NMR (DMSO‐*d*_6_): *δ* 9.10, 13.84 (2CH_3_), 45.7, 61.4, 87.8, 113.5, 113.7, 115.2, 121.4, 126.6, 128.7, 129.0, 129.3, 129.5, 132.0, 135.1, 135.9, 138.8, 141.0, 141.2 (Ar‐C), 157.1 (C=O), 171.8 (C=S) ppm; MS m/z (%) 487 (M^+^, 23), 473 (20), 458 (23), 444 (38), 418 (58), 378 (59), 375 (55), 307 (45), 231 (73), 210 (40), 190 (40), 173 (66), 167 (34), 142 (100), 89 (56). Anal. Calcd. for C_25_H_21_N_5_O_2_S_2_ (487.60): C, 61.58; H, 4.34; N, 14.36. Found C, 61.67; H, 4.31; N, 14.32%.

##### 3.1.2.2 3‐(1‐(2‐(4‐Methyl‐5‐(1‐methyl‐6‐phenyl‐2‐thioxo‐1,2,3,6‐tetrahydropyrimidin‐4‐yl)thiazol‐2‐yl)hydrazono)ethyl)‐2H‐chromen‐2‐one (7)

Red solid; m.p. 167°C–168°C (EtOH); IR (KBr): *v* 3444, 3356 (2NH), 3066, 2920 (C‐H), 1651 (C=O) cm^−1^; ^1^H NMR (DMSO‐*d*_6_): *δ* 2.4 (s, 3H, CH_3_), 3.31 (s, 3H, CH_3_), 3.54 (s, 3H, CH_3_), 3.79 (s, 1H, CH‐CH=C), 6.67 (s, 1H, CH‐CH=C), 7.49–7.83 (m, 10H, Ar‐H, coumarin‐H), 8.53 (s, 1H, NH), 12.20 (br s, 1H, NH) ppm; ^13^C NMR (DMSO‐*d*_6_): *δ* 19.5, 22.1, 31.5 (3CH_3_), 66.8, 115.2, 121.3, 121.5, 123.3, 123.5, 124.6, 128.4, 128.7, 129.2, 129.8, 129.9, 130.0, 131.9, 135.0, 135.2, 135.5, 138.8, 142.7 (Ar‐C), 172.2 (C=O), 179.6 (C=S) ppm; MS m/z (%) 501 (M^+^, 15), 418 (20), 374 (12), 317 (14), 284 (70), 244 (18), 206 (63), 180 (20), 87 (39), 86 (81), 70 (100), 57 (77), 45 (50). Anal. Calcd. for C_26_H_23_N_5_O_2_S_2_ (501.62): C, 62.26; H, 4.62; N, 13.96. Found C, 62.34; H, 4.56; N, 13.85%.

##### 3.1.2.3 3‐(4‐Methyl‐2‐(2‐(1‐(2‐oxo‐2H‐chromen‐3‐yl)ethylidene)hydrazinyl)thiazol‐5‐yl)‐5‐phenyl‐4,5‐dihydro‐1H‐pyrazole‐1‐carbothioamide (9)

Red solid; m.p. 180°C–181°C (EtOH); IR (KBr): *v* 3425 (NH), 3325, 3255 (NH_2_), 3077, 2915 (C‐H), 1654 (C=O) cm^−1^; ^1^H NMR (DMSO‐*d*_6_): *δ* 2.19 (s, 3H, CH_3_), 2.25 (s, 3H, CH_3_), 2.94 (s, 1H, CH‐CH=C), 3.47 (m, 2H, CH_2_‐pyrazole), 6.88 (d,t, *J* = 6.9, 0.6 Hz, 1H, NH_2_), 7.32–7.39 (m, 10H, Ar‐H), 7.50 (d,t, *J* = 6.9, 0.6 Hz, 1H, NH_2_), 8.86 (s, 1H, coumarin‐H), 11.10 (br s, 1H, NH) ppm; ^13^C NMR (DMSO‐*d*_6_): *δ* 23.9, 35.4 (2CH_3_), 79.5, 112.6, 120.5, 127.6, 128.4, 128.7, 129.0, 129.4, 129.8, 130.7, 131.2, 132.4, 132.5, 132.7, 132.9, 135.1, 136.7, 148.4, 153.8 (Ar‐C), 156.2 (C=O), 157.3 (C=S) ppm; MS m/z (%) 500 (M^+^, 20), 468 (40), 450 (61), 425 (52), 399 (74), 385 (62), 373 (60), 348 (83), 342 (75), 329 (72), 266 (51), 214 (44), 204 (82), 121 (100), 75 (90). Anal. Calcd. for C_25_H_22_N_6_O_2_S_2_ (502.12): C, 59.74; H, 4.41; N, 16.72. Found C, 59.82; H, 4.37; N, 16.69%.

##### 3.1.2.4 3‐(4‐Methyl‐2‐(2‐(1‐(2‐oxo‐2H‐chromen‐3‐yl)ethylidene)hydrazinyl)thiazol‐5‐yl)‐5‐phenyl‐4,5‐dihydro‐1H‐pyrazole‐1‐carbothiohydrazide (11)

Red solid; m.p. 172°C–173°C (EtOH); IR (KBr): *v* 3394, 3325 (2NH), 3267, 3213 (NH_2_), 3089, 3032 (C‐H), 1627 (C=O) cm^−1^; ^1^H NMR (DMSO‐*d*_6_): *δ* 2.06 (s, 3H, CH_3_), 2.32 (s, 3H, CH_3_), 3.31 (m, 2H, CH_2_‐pyrazole), 4.18 (s, 1H, CH‐CH=C), 6.90 (d,t, *J* = 6.9, 0.6 Hz, 1H, NH_2_), 7.03–7.60 (m, 10H, Ar‐H, coumarin‐H), 7.49 (d,t, *J* = 6.9, 0.6 Hz, 1H, NH_2_), 7.35 (s, 1H, NH), 10.90 (br s, 1H, NH) ppm; MS m/z (%) 515 (M^+^, 31), 478 (62), 456 (29), 426 (16), 341 (16), 223 (69), 193 (46), 120 (100), 102 (66), 65 (85). Anal. Calcd. for C_25_H_23_N_7_O_2_S_2_ (517.63): C, 58.14; H, 4.48; N, 18.94. Found C, 58.21; H, 4.39; N, 18.88%.

##### 3.1.2.5 7‐(4‐Methyl‐2‐(2‐(1‐(2‐oxo‐2H‐chromen‐3‐yl)ethylidene)hydrazinyl)thiazol‐5‐yl)‐5‐phenyl‐2‐thioxo‐2,3‐dihydropyrido[2,3‐d]pyrimidin‐4(1H)‐one (13)

Red solid; m.p. 222°C–223°C (DMF); IR (KBr): *v* 3421, 3334, 3288 (3NH), 3044, 2982 (C‐H), 1650, 1688 (2C=O) cm^−1^; ^1^H NMR (DMSO‐*d*_6_): *δ* 2.04 (s, 3H, CH_3_), 2.62 (s, 3H, CH_3_), 6.83–7.58 (m, 11H, Ar‐H, coumarin‐H), 7.08 (s, 1H, NH), 11.00 (br s, 2H, 2NH) ppm; ^13^C NMR (DMSO‐*d*_6_): *δ* 30.6, 33.2 (2CH3), 116.7, 119.2, 119.6, 120.0, 120.8, 121.8, 128.9, 130.3, 130.5, 130.7, 131.4, 131.8, 132.0, 134.3, 137.4, 141.3, 150.4, 153.1, 154.4, 155.6, 156.7 (Ar‐C), 160.0, 166.9 (2C=O), 168.3 (C=S) ppm; MS m/z (%) 552 (M^+^, 19), 486 (24), 436 (19), 355 (29), 348 (40), 316 (45), 262 (39), 230 (42), 192 (88), 157 (43), 119 (35), 90 (40), 76 (100). Anal. Calcd. For C_28_H_20_N_6_O_3_S_2_ (552.63): C, 60.86; H, 3.65; N, 15.21. Found C, 60.93; H, 3.58; N, 15.17%.

#### 3.1.3 General Procedure for the Synthesis of Triazolopyrimidine Derivatives 17a‐g and 18a‐e

To a hot 30 mL dioxane solution of pyrimidinethione derivatives **13** or **5** (10 mmol), containing 2 mL of triethylamine, a series of hydrazonyl halide derivatives **14a‐g** or **14a‐e** (10 mmol) were added. The resulting mixture was refluxed vigorously for 6 h. Upon completion of the reaction, the mixture was allowed to cool to room temperature, and the formed precipitate was collected and recrystallized from acetic acid to give the pure triazolopyrimidine derivatives **17a‐g** or **18a‐e**.

##### 3.1.3.1 3‐Acetyl‐1‐(4‐methoxyphenyl)‐8‐(4‐methyl‐2‐(2‐(1‐(2‐oxo‐2H‐chromen‐3‐yl) ethylidene)hydrazinyl)thiazol‐5‐yl)‐6‐phenyl‐6,9‐dihydropyrido[2,3‐d][1,2,4]triazolo [4,3‐a]pyrimidin‐5(1H)‐one (17a)

Brown solid; m.p. 160°C–161°C; IR (KBr): *v* 3367, 3348 (2NH), 2962, 2931 (C‐H), 1720, 1670, 1642 (3C=O) cm^−1^; ^1^H‐NMR (DMSO‐*d*_6_): *δ* 2.26 (s, 3H, CH_3_), 2.36 (s, 3H, CH_3_), 2.46 (s, 3H, CH_3_), 3.64 (s, 3H, OCH_3_), 6.84–7.96 (m, 14H, Ar‐H, coumarin‐H), 8.40 (s, 1H, pyridine‐H), 11.32 (br s, 1H, NH) ppm; MS m/z (%) 710 (M^+^, 7), 693 (27), 650 (23), 574 (40), 550 (43), 460 (63), 443 (42), 439 (54), 422 (92), 333 (57), 292 (100), 204 (61), 114 (41), 99 (87), 79 (52). Anal. Calcd. for C_38_H_30_N_8_O_5_S (710.77): C, 64.21; H, 4.25; N, 15.77. Found C, 64.30; H, 4.18; N, 15.72%.

##### 3.1.3.2 3‐Acetyl‐1‐(4‐chlorophenyl)‐8‐(4‐methyl‐2‐(2‐(1‐(2‐oxo‐2H‐chromen‐3‐yl)ethylidene) hydrazinyl)thiazol‐5‐yl)‐6‐phenyl‐6,9‐dihydropyrido[2,3‐d][1,2,4]triazolo[4,3‐a]pyrimidin‐5(1H)‐one (17b)

Brown solid; m.p. 175°C–176°C; IR (KBr): *v* 3367, 3217 (2NH), 3016, 2986 (C‐H), 1692, 1654, 1625 (3C=O) cm^−1^; ^1^H‐NMR (DMSO‐*d*_6_): *δ* 2.21 (s, 3H, CH_3_), 2.34 (s, 3H, CH_3_), 2.41 (s, 3H, CH_3_), 7.13–7.71 (m, 14H, Ar‐H, coumarin‐H), 8.14 (s, 1H, pyridine‐H), 11.64 (br s, 1H, NH) ppm; MS m/z (%) 715 (M^+^, 17), 709 (26), 685 (20), 575 (12), 541 (12), 492 (12), 454 (15), 366 (17), 308 (24), 288 (19), 267 (62), 233 (90), 144 (100), 121 (25), 49 (73). Anal. Calcd. for C_37_H_27_ClN_8_O_4_S (715.18): C, 62.14; H, 3.81; N, 15.67. Found C, 62.25; H, 3.76; N, 15.60%.

##### 3.1.3.3 3‐Acetyl‐1‐(2,4‐dichlorophenyl)‐8‐(4‐methyl‐2‐(2‐(1‐(2‐oxo‐2H‐chromen‐3‐yl) ethylidene)hydrazinyl)thiazol‐5‐yl)‐6‐phenyl‐6,9‐dihydropyrido[2,3‐d][1,2,4]triazolo [4,3‐a]pyrimidin‐5(1H)‐one (17c)

Brown solid; m.p. 189°C–190°C; IR (KBr): *v* 3461, 3355 (2NH), 3070, 2963 (C‐H), 1718, 1658, 1633 (3C=O) cm^−1^; ^1^H‐NMR (DMSO‐*d*_6_): *δ* 2.19 (s, 3H, CH_3_), 2.61 (s, 3H, CH_3_), 2.70 (s, 3H, CH_3_), 7.05–7.73 (m, 13H, Ar‐H, coumarin‐H), 8.20 (s, 1H, pyridine‐H), 12.33 (br s, 1H, NH) ppm; MS m/z (%) 749 (M^+^, 16), 698 (14), 676 (19), 619 (28), 560 (18), 522 (26), 502 (100), 485 (49), 364 (42), 207 (34), 180 (46), 148 (31). Anal. Calcd. for C_37_H_26_Cl_2_N_8_O_4_S (749.63): C, 59.28; H, 3.50; N, 14.95. Found C, 59.33; H, 3.44; N, 14.89%.

##### 3.1.3.4 Ethyl‐8‐(4‐methyl‐2‐(2‐(1‐(2‐oxo‐2H‐chromen‐3‐yl)ethylidene)hydrazinyl)thiazol‐5‐yl)‐5‐oxo‐6‐phenyl‐1‐(p‐tolyl)‐1,5,6,9‐tetrahydropyrido[2,3‐d][1,2,4]triazolo[4,3‐a]pyrimidine‐3‐carboxylate (17d)

Brown solid; m.p. 147°C–148°C; IR (KBr): *v* 3364, 3174 (2NH), 3062, 3035 (C‐H), 1747, 1681, 1643 (3C=O) cm^−1^; ^1^H‐NMR (DMSO‐*d*_6_): *δ* 1.13–1.18 (*t*, 3H, CH_3_), 2.24 (s, 3H, CH_3_), 2.42 (s, 3H, CH_3_), 2.60 (s, 3H, CH_3_), 4.04–4.15 (d, 2H, CH_2_), 6.68–7.96 (m, 14H, Ar‐H, coumarin‐H), 8.52 (s, 1H, pyridine‐H), 11.40 (br s, 1H, NH) ppm; MS m/z (%) 724 (M^+^, 9), 695 (28), 689 (45), 663 (44), 639 (61), 620 (61), 580 (24), 569 (43), 418 (49), 388 (29), 375 (100), 313 (33), 199 (69), 152 (62), 106 (34), 57 (57). Anal. Calcd. for C_39_H_32_N_8_O_5_S (724.80): C, 64.63; H, 4.45; N, 15.46. Found C, 64.70; H, 4.39; N, 15.38%.

##### 3.1.3.5 Ethyl‐1‐(4‐chlorophenyl)‐8‐(4‐methyl‐2‐(2‐(1‐(2‐oxo‐2H‐chromen‐3‐yl)ethylidene) hydrazinyl)thiazol‐5‐yl)‐5‐oxo‐6‐phenyl‐1,5,6,9‐tetrahydropyrido[2,3‐d][1,2,4]triazolo [4,3‐a]pyrimidine‐3‐carboxylate (17e)

Brown solid; m.p. 195°C–196°C; IR (KBr): *v* 3364, 3174 (2NH), 3062, 3035 (C‐H), 1747, 1681, 1643 (3C=O) cm^−1^; ^1^H‐NMR (DMSO‐*d*_6_): *δ* 1.15–1.24 (*t*, 3H, CH_3_), 2.25 (s, 3H, CH_3_), 2.48 (s, 3H, CH_3_), 2.64 (s, 3H, CH_3_), 4.04–4.20 (d, 2H, CH_2_), 6.67–7.95 (m, 14H, Ar‐H, coumarin‐H), 8.10 (s, 1H, pyridine‐H), 10.87 (br s, 1H, NH) ppm; MS m/z (%) 745 (M^+^, 11), 726 (16), 669 (16), 579 (16), 562 (14), 503 (29), 455 (92), 393 (50), 381 (54), 337 (100), 287 (61), 174 (24). Anal. Calcd. for C_38_H_29_ClN_8_O_5_S (745.21): C, 61.25; H, 3.92; N, 15.04. Found C, 61.34; H, 3.87; N, 15.00%.

##### 3.1.3.6 8‐(4‐Methyl‐2‐(2‐(1‐(2‐oxo‐2H‐chromen‐3‐yl)ethylidene)hydrazinyl)thiazol‐5‐yl)‐5‐oxo‐N,1,6‐triphenyl‐1,5,6,9‐tetrahydropyrido[2,3‐d][1,2,4]triazolo[4,3‐a]pyrimidine‐3‐carboxamide (17f)

Brown solid; m.p. 179°C–181°C; IR (KBr): *v* 3385, 3329, 3244 (3NH), 3050, 3028 (C‐H), 1730, 1661, 1600 (3C=O) cm^−1^; ^1^H‐NMR (DMSO‐*d*_6_): *δ* 2.40 (s, 3H, CH_3_), 2.68 (s, 3H, CH_3_), 6.64–7.98 (m, 20H, Ar‐H, coumarin‐H), 8.71 (s, 1H, pyridine‐H), 10.22 (s, 1H, NH), 11.66 (br s, 1H, NH) ppm; MS m/z (%) 757 (M^+^, 45), 747 (31), 724 (44), 697 (44), 690 (45), 647 (30), 604 (72), 543 (38), 490 (40), 439 (46), 339 (84), 257 (41), 203 (63), 110 (40), 71 (41), 40 (100). Anal. Calcd. for C_42_H_31_N_9_O_4_S (757.83): C, 66.57; H, 4.12; N, 16.63. Found C, 66.63; H, 4.07; N, 16.58%.

##### 3.1.3.7 8‐(4‐Methyl‐2‐(2‐(1‐(2‐oxo‐2H‐chromen‐3‐yl)ethylidene)hydrazinyl)thiazol‐5‐yl)‐1‐(4‐nitrophenyl)‐5‐oxo‐N,6‐diphenyl‐1,5,6,9‐tetrahydropyrido[2,3‐d][1,2,4]triazolo[4,3‐a]pyrimidine‐3‐carboxamide (17g)

Brown solid; m.p. 187°C–188°C; IR (KBr): *v* 3454, 3362, 3268 (3NH), 3068, 3035 (C‐H), 1730, 1661, 1620 (3C=O) cm^−1^; ^1^H‐NMR (DMSO‐*d*_6_): *δ* 2.32 (s, 3H, CH_3_), 2.61 (s, 3H, CH_3_), 7.24–8.50 (m, 19H, Ar‐H, coumarin‐H), 9.22 (s, 1H, pyridine‐H), 10.40 (s, 1H, NH), 11.47 (br s, 1H, NH) ppm; MS m/z (%) 802 (M^+^, 11), 749 (25), 662 (29), 558 (25), 504 (42), 471 (21), 414 (64), 403 (70), 388 (40), 348 (75), 338 (100), 294 (84), 174 (34), 109 (24), 93 (41), 62 (47). Anal. Calcd. for C_42_H_30_N_10_O_6_S (802.83): C, 62.84; H, 3.77; N, 17.45. Found C, 62.91; H, 3.69; N, 17.39%.

##### 3.1.3.8 3‐(1‐(2‐(5‐(3‐Acetyl‐1‐(4‐chlorophenyl)‐5‐phenyl‐1,5‐dihydro‐[1,2,4]triazolo[4,3‐a]pyrimidin‐7‐yl)‐4‐methylthiazol‐2‐yl)hydrazono)ethyl)‐2H‐chromen‐2‐one (18a)

Brown solid; m.p. 150°C–151°C; IR (KBr): *v* 3163 (NH), 3032, 2970 (C‐H), 1724, 1647 (2C=O) cm^−1^; ^1^H‐NMR (DMSO‐*d*_6_): *δ* 2.22 (s, 3H, CH_3_), 2.34 (s, 3H, CH_3_), 2.60 (s, 3H, CH_3_), 7.01–7.80 (m, 14H, Ar‐H, coumarin‐H), 8.22 (s, 1H, pyrimidin‐H), 11.35 (s, 1H, NH) ppm; MS m/z (%) 648 (M^+^, 14), 634 (17), 574 (39), 539 (56), 503 (34), 460 (33), 407 (100), 379 (32), 310 (41), 306 (68), 281 (74), 203 (42), 146 (45), 105 (34), 83 (61), 66 (51). Anal. Calcd. for C_34_H_26_ClN_7_O_3_S (648.14): C, 63.01; H, 4.04; N, 15.13. Found C, 63.10; H, 4.00; N, 15.08%.

##### 3.1.3.9 3‐(1‐(2‐(5‐(3‐Acetyl‐5‐phenyl‐1‐(p‐tolyl)‐1,5‐dihydro‐[1,2,4]triazolo[4,3‐a]pyrimidin‐7‐yl)‐4‐methylthiazol‐2‐yl)hydrazono)ethyl)‐2H‐chromen‐2‐one (18b)

Brown solid; m.p. 165°C–166°C; IR (KBr): *v* 3200 (NH), 3038, 2977 (C‐H), 1727, 1654 (2C=O) cm^−1^; ^1^H‐NMR (DMSO‐*d*_6_): *δ* 2.19 (s, 3H, CH_3_), 2.28 (s, 3H, CH_3_), 2.44 (s, 3H, CH_3_), 2.67 (s, 3H, CH_3_), 7.11–7.77 (m, 14H, Ar‐H, coumarin‐H), 8.34 (s, 1H, pyrimidin‐H), 11.47 (s, 1H, NH) ppm; MS m/z (%) 627 (M^+^, 14), 605 (59), 574 (30), 563 (59), 548 (100), 537 (46), 506 (53), 465 (51), 425 (34), 316 (34), 308 (68), 262 (30), 199 (54), 100 (96), 87 (44). Anal. Calcd. for C_35_H_29_N_7_O_3_S (627.72): C, 66.97; H, 4.66; N, 15.62. Found C, 67.06; H, 4.61; N, 15.56%.

##### 3.1.3.10 Ethyl‐1‐(4‐chlorophenyl)‐7‐(4‐methyl‐2‐(2‐(1‐(2‐oxo‐2H‐chromen‐3‐yl)ethylidene) hydrazinyl)thiazol‐5‐yl)‐5‐phenyl‐1,5‐dihydro‐[1,2,4]triazolo[4,3‐a]pyrimidine‐3‐carboxylate (18c)

Brown solid; m.p. 150°C–151°C; IR (KBr): *v* 3174 (NH), 3032, 2981 (C‐H), 1728, 1666 (2C=O) cm^−1^; ^1^H‐NMR (DMSO‐*d*_6_): *δ* 1.16–1.34 (t, 3H, CH_3_), 2.20 (s, 3H, CH_3_), 2.33 (s, 3H, CH_3_), 4.16–4.28 (d, 2H, CH_2_), 7.09–7.95 (m, 14H, Ar‐H, coumarin‐H), 8.25 (s, 1H, pyrimidin‐H), 10.84 (s, 1H, NH) ppm; MS m/z (%) 678 (M^+^, 5), 632 (25), 629 (17), 578 (20), 525 (45), 471 (45), 463 (44), 457 (100), 346 (19), 265 (62), 184 (25), 95 (56), 92 (48), 75 (46), 61 (47). Anal. Calcd. for C_35_H_28_ClN_7_O_4_S (678.16): C, 61.99; H, 4.16; N, 14.46. Found C, 62.04; H, 4.12; N, 14.39%.

##### 3.1.3.11 Ethyl‐7‐(4‐methyl‐2‐(2‐(1‐(2‐oxo‐2H‐chromen‐3‐yl)ethylidene)hydrazinyl)thiazol‐5‐yl)‐5‐phenyl‐1‐(p‐tolyl)‐1,5‐dihydro‐[1,2,4]triazolo[4,3‐a]pyrimidine‐3‐carboxylate (18d)

Brown solid; m.p. 177°C–178°C; IR (KBr): *v* 3170 (NH), 3066, 2981 (C‐H), 1724, 1639 (2C=O) cm^−1^; ^1^H‐NMR (DMSO‐*d*_6_): *δ* 1.11–1.30 (t, 3H, CH_3_), 2.09 (s, 3H, CH_3_), 2.35 (s, 3H, CH_3_), 2.40 (s, 3H, CH_3_), 4.20–4.35 (d, 2H, CH_2_), 7.38–7.67 (m, 14H, Ar‐H, coumarin‐H), 8.16 (s, 1H, pyrimidin‐H), 11.50 (s, 1H, NH) ppm; MS m/z (%) 657 (M^+^, 18), 644 (13), 565 (11), 552 (10), 437 (42), 396 (100), 320 (87), 281 (35), 279 (72), 239 (32), 199 (25), 82 (35), 71 (72), 56 (57), 42 (60). Anal. Calcd. for C_36_H_31_N_7_O_4_S (657.75): C, 65.74; H, 4.75; N, 14.91. Found C, 65.78; H, 4.69; N, 15.00%.

##### 3.1.3.12 7‐(4‐Methyl‐2‐(2‐(1‐(2‐oxo‐2H‐chromen‐3‐yl)ethylidene)hydrazinyl)thiazol‐5‐yl)‐1‐(4‐nitrophenyl)‐N,5‐diphenyl‐1,5‐dihydro‐[1,2,4]triazolo[4,3‐a]pyrimidine‐3‐carboxamide (18e)

Brown solid; m.p. 163°C–164°C; IR (KBr): *v* 3288, 3186 (NH), 3070, 2981 (C‐H), 1724, 1663 (2C=O) cm^−1^; ^1^H‐NMR (DMSO‐*d*_6_): *δ* 2.27 (s, 3H, CH_3_), 2.39 (s, 3H, CH_3_), 7.01–7.80 (m, 14H, Ar‐H, coumarin‐H), 8.22 (s, 1H, pyrimidin‐H), 10.50 (s, 1H, NH), 11.44 (s, 1H, NH) ppm; MS m/z (%) 735 (M^+^, 42), 718 (48), 708 (37), 673 (44), 656 (63), 617 (43), 525 (69), 513 (40), 469 (47), 440 (39), 397 (100), 374 (52), 355 (31), 306 (36), 270 (93), 245 (34), 141 (45), 57 (34), 46 (71). Anal. Calcd. for C_39_H_29_N_9_O_5_S (735.78): C, 63.66; H, 3.97; N, 17.13. Found C, 63.71; H, 3.92; N, 17.09%.

### 3.2 Biological Evaluation

#### 3.2.1 In Vitro Cytotoxic Activity

The cytotoxic activity of the synthesized thiazol‐hydrazono‐coumarin derivatives was evaluated against the human cervical cancer cell line HeLa (ATCC, Rockville, MD) using the standard MTT colorimetric assay (Mosmann 1983). Cells were cultured in RPMI‐1640 medium supplemented with 10% inactivated fetal bovine serum and 50 μg/mL gentamycin (Lonza, Belgium), and maintained in a humidified atmosphere at 37°C with 5% CO₂.

For the assay, HeLa cells were seeded at a density of 5 × 10^4^ cells/well in Corning 96‐well tissue culture plates and incubated for 24 h. Test compounds were dissolved in DMSO and serially diluted in culture medium to achieve twelve concentrations per compound. Each concentration was tested in triplicate. Six vehicle control wells containing 0.5% DMSO were included on each plate.

After 24 h of compound exposure, the medium was removed and replaced with 100 μL of fresh phenol red‐free RPMI 1640. Then, 10 μL of a 12 mM MTT stock solution (prepared in PBS) was added to each well, including untreated controls. Next, plates were incubated at 37 C with 5% CO₂ for an additional 4 h. Subsequently, 85 μL of the medium was removed from each well and replaced with 50 μL of DMSO. The contents were mixed thoroughly by pipetting and incubated at 37°C for 10 min to solubilize the formazan crystals. Absorbance was measured at 590 nm using a microplate reader (SunRise, TECAN, USA). Cell viability was expressed as a percentage relative to untreated controls, and IC_50_ values were calculated by non‐linear regression analysis using GraphPad Prism (GraphPad Software, San Diego, CA, USA).

#### 3.2.2 Antibacterial Activity and MIC Determination Against *Staphylococcus aureus*

The antibacterial efficacy of the synthesized compounds was evaluated against *Staphylococcus aureus* strains, including methicillin‐sensitive (MSSA, ATCC 29213), methicillin‐resistant (MRSA, ATCC 700788), and vancomycin‐resistant (VRSA, RCMB 28354), using the broth microdilution method coupled with the XTT reduction assay. The extensively drug‐resistant VRSA strain was obtained from the culture collection unit of the Regional Center for Mycology and Biotechnology (RCMB).

Overnight bacterial cultures were prepared in Brain Heart Infusion (BHI) medium (Oxoid, UK) at 37°C. The bacterial inoculum was adjusted to 10^6^ CFU/mL in tryptic soy broth (TSB). Serial dilutions of the test compounds were prepared in DMSO and added (50 μL per well) to 96‐well microtiter plates containing 100 μL of TSB. Each well was then inoculated with 50 μL of the bacterial suspension. Final compound concentrations ranged from 1000 to 0.24 μg/mL.

Following a 24‐h incubation at 37°C in the dark, 100 μL of freshly prepared XTT reagent (0.5 g/L in Ringer's lactate, filtered through a 0.22 μm membrane) was added to each well, and plates were incubated for an additional hour. Absorbance was measured at 492 nm using a BioTek microplate reader (USA). Percent inhibition was calculated as:

$$\%\text{Inhibition}=\left[1-\left(\frac{{\mathrm{OD}}_{t}}{{\mathrm{OD}}_{c}}\right)\right]\times 100$$

where OD_t_ and OD_c_ refer to absorbance in treated and untreated control wells, respectively. MIC was defined as the lowest concentration yielding 100% inhibition of microbial growth relative to the control (Abuelizz et al. 2021).

#### 3.2.3 Antibacterial Screening Against Gram‐Negative bacteria

The antibacterial activity of the synthesized coumarin derivatives against Gram‐negative bacteria was evaluated using the agar well diffusion method. The tested strains included *Klebsiella pneumoniae* (ATCC 13883), *Pseudomonas aeruginosa* (ATCC 27853), *Acinetobacter baumannii* (ATCC 19606), *Salmonella typhimurium* (ATCC 14028), and *Escherichia coli* (ATCC 25922). All bacterial strains were cultured overnight in nutrient broth at 37°C prior to the assay.

Mueller‐Hinton agar (MHA) plates were prepared and inoculated with 100 μL of each bacterial suspension (10^6^ CFU/mL), evenly spread using a sterile cotton swab. Wells (6 mm diameter) were aseptically punched into the agar and filled with 50 μL of each compound solution (10 μg/mL in DMSO). Gentamycin (10 μg/mL) was used as a positive control, while DMSO served as a negative control. The plates were incubated at 37°C for 24 h, after which the diameter of the zone of inhibition (including the well diameter) was measured in millimeters (Magaldi et al. 2004).

All tests were performed in triplicate, and results were expressed as mean inhibition zone diameters. Compounds exhibiting inhibition zones greater than 20 mm were considered to have strong antibacterial activity.

### 3.3 Molecular Modeling

All computational studies were conducted on a DELL G3‐3500 workstation (Intel Core i5, 2.50 GHz, 16 GB RAM).

#### 3.3.1 Molecular Data Preparation

The 17 novel coumarin derivatives were sketched in ACD/ChemSketch v2024.2.3 and prepared using Discovery Studio Visualizer v25.1.0. An initial curated set of 104 bacterial DNA gyrase, 6 bacterial topoisomerase IV, and 11 human topoisomerase II crystal structures was downloaded from the RCSB Protein Data Bank (PDB) (RCSB Protein Data Bank, n.d.). Structures containing a GyrB or ParE subunit co‐crystallized with a non‐fragment ligand were retained, resulting in a final curated set of 44 GyrB, 6 topoisomerase IV, and 8 topoisomerase II structures. The corresponding 58 co‐crystallized ligands were extracted, combined with the 17 novel coumarins and 20 additional curated‐out ligands to form a 95‐compound SDF library. All ligands were preprocessed using Discovery Studio Visualizer and merged with Open Babel v3.1.0 (O'Boyle et al. 2011).

#### 3.3.2 Crystal Structure Selection

DataWarrior v6.04.02 was used to perform similarity analysis (FragFp, SkelSpheres, and Flexophore fingerprints) to identify crystal structures with binding sites most similar to the novel coumarins (Sander et al. 2015; von Korff et al. 2008). Protein sequences were aligned using MUSCLE in Jalview v2.11.4.1, and residue conservation was mapped onto 3D structures with PyMOL v3.1.0 to identify conserved pharmacophoric residues across the three enzyme families (PyMOL.org, n.d.; Waterhouse et al. 2009). One representative crystal structure per species and enzyme was selected, yielding eight targets: 7PQL ( *A. baumannii* GyrB), 6F94 ( *E. coli* GyrB), 1S14 ( *E. coli* topoisomerase IV), 1ZXM ( *H. sapiens* topoisomerase II), 7PTG ( *P. aeruginosa* GyrB), 6TCK ( *S. aureus* GyrB), and 4URN ( *S. aureus* topoisomerase IV).

#### 3.3.3 Molecular Docking

The five lead coumarins (compounds 11, 13, 17c, 17e, and 18b) were docked into each of the selected targets using Flare v10.0.1 (Extra Precision mode, 10 runs, 30 max poses, other parameters at default) (Bauer and Mackey 2019; Cheeseright et al. 2006; Kuhn et al. 2020). Protein preparation included the removal of crystallographic water and artifacts in PyMOL, followed by default preparation in Flare, which involved protonation and modeling of minor missing residue gaps. Each co‐crystallized ligand was redocked into its protein to validate the docking protocol. RMSD values between the crystallographic and redocked poses were computed using DockRMSD v1.1 (Bell and Zhang 2019). Docking poses were ranked based on Flare scores, pose clustering, and agreement with known ligand‐protein interactions. One pose per ligand per structure was selected (two per ligand in 1ZXM: one coordinating the catalytic Mg^2+^ and one adjacent).

#### 3.3.4 MM/GBSA Calculations

Binding free energies and per‐residue energy decompositions were computed using Uni‐GBSA v0.1.7 in decomposition mode (Yang et al. 2023), using the default GBSA settings, with the GB model based on parameters recommended by Wang et al. (Wang et al. 2019).

#### 3.3.5 Molecular Dynamics Simulation

The protein‐ligand complex from 6TCK ( *S. aureus* GyrB) and compound 13 was subjected to a 100 ns molecular dynamics simulation using StreaMD v0.2.6 (Ivanova et al. 2024). The default StreaMD protocols for force field, solvation, and PBC treatment were employed. Trajectory visualization and interaction inspection were performed using UCSF ChimeraX v1.9 (Meng et al. 2023).

#### 3.3.6 Statistical Analysis and Molecular Visualization

Statistical analyses and energy plots from MD simulations were generated automatically by StreaMD's integrated Python modules. Figures of protein‐ligand complexes and protein overlays were prepared using Flare, PyMOL, and ChimeraX.

#### 3.3.7 Pharmacokinetics and Toxicity Prediction

Pharmacokinetic properties of compound 13 were predicted using the SwissADME server, and the toxicity profile of compound 13 was estimated using the ProTox 3.0 server (Banerjee et al. 2024; Daina et al. 2017).

## 4 Conclusion

In this study, a novel series of thiazol‐hydrazono‐coumarin derivatives was synthesized and evaluated for their dual anticancer and antibacterial activities. Several compounds, most notably compound 13, exhibited potent cytotoxic effects against HeLa cells, with significantly higher selectivity indices relative to normal human lung fibroblast cells (WI‐38), indicating a favorable therapeutic window. A subset of derivatives also demonstrated strong antibacterial activity against methicillin‐sensitive, methicillin‐resistant, and vancomycin‐resistant *Staphylococcus aureus* strains, with minimum inhibitory concentrations (MICs) reaching the sub‐microgram per milliliter range, surpassing the performance of vancomycin in certain cases.

Molecular docking and MM/GBSA binding free energy calculations revealed that the five most active compounds (11, 13, 17c, 17e, and 18b) bind favorably within the ATP‐binding pockets of bacterial DNA gyrase and topoisomerase IV, adopting conserved poses consistent with known GyrB inhibitors. Compound 13, in particular, displayed strong predicted affinity across multiple bacterial targets, as well as for the human topoisomerase IIα, providing a molecular rationale for its dual bioactivity profile. Comparative sequence and structure alignment uncovered exploitable differences between bacterial and human topoisomerases, especially at pharmacophoric residues such as serine‐129, which engaged in a novel hydrogen‐bond interaction with the coumarin ring of compound 13 as revealed by molecular dynamics (MD) simulations. The MD results further confirmed the structural stability of the 13‐GyrB complex over a 100 ns trajectory, with minimal RMSD fluctuations and persistent protein‐ligand interactions that support the predicted binding pose.

Chemical similarity analysis further supported the hypothesis that these derivatives form a structurally homogeneous cluster with potentially conserved binding behavior, while their divergence from known co‐crystallized ligands highlights their novelty. In silico ADMET profiling of compound 13 indicated low acute toxicity, no significant interactions with major CYP450 isoforms, and broadly acceptable drug‐likeness, albeit specific structural alerts advised caution during lead optimization.

Collectively, these integrated experimental and computational findings position thiazol‐hydrazono‐coumarins as promising multifunctional therapeutic leads with dual activity against bacterial and human topoisomerases. Future work will focus on further structural optimization, in vivo efficacy testing, and mechanistic elucidation of their polypharmacological potential in relevant cancer‐infection models.

## Author Contributions

**Islam K. Matar:** conceptualization (equal); methodology (equal); investigation (equal); software (lead); formal analysis (equal); data curation (equal); visualization (equal); validation (equal); project administration (equal); funding acquisition (supporting); writing – original draft (equal); writing – review and editing (equal). **Magdi E. A. Zaki:** funding acquisition (lead); supervision (equal); project administration (equal); investigation (equal); visualization (equal); writing – review and editing (equal). **Zeinab A. Muhammad:** conceptualization (equal); methodology (equal); formal analysis (equal); data curation (equal); resources (equal); validation (equal); visualization (equal); project administration (equal); writing – original draft (equal); writing – review and editing (equal). **Dahlia A. Awwad:** conceptualization (equal); software (supporting); formal analysis (equal); visualization (equal); writing – original draft (equal). **Sami A. Al‐Hussain:** formal analysis (equal); data curation (equal); funding acquisition (supporting); writing – original draft (equal). **Chérif F. Matta:** supervision (equal); resources (equal); funding acquisition (supporting); writing – review and editing (equal). **Refaie M. Kassab:** conceptualization (equal); investigation (equal); data curation (equal); resources (equal); supervision (equal); writing – original draft (equal); writing – review and editing (equal).

## Funding

This work was supported and funded by the Deanship of Scientific Research at Imam Mohammad Ibn Saud Islamic University (IMSIU) (Grant IMSIU‐DDRSP2501).

## Disclosure

The authors declare the use of OpenAI's ChatGPT as a tool for paraphrasing and condensing selected passages.

## Conflicts of Interest

The authors declare no conflicts of interest.

## Supporting information


**Data S1:** cbdd70261‐sup‐0001‐DataS1.docx.

**Video S1:** cbdd70261‐sup‐0002‐VideoS1.mp4.

**Data S3:** cbdd70261‐sup‐0003‐DataS3.pdf.pdf.

**Data S4:** cbdd70261‐sup‐0004‐DataS4.pdf.pdf.

## Data Availability

The data that supports the findings of this study are available in the Supporting Information of this article.

## References

[cbdd70261-bib-0001] Abdelhamid, A. O. , E. K. A. Abdelall , N. A. Abdel‐Riheem , and S. A. Ahmed . 2010. “Synthesis and Antimicrobial Activity of Some New 5‐Arylazothiazole, Pyrazolo[1,5‐a] Pyrimidine, [1,2,4]Triazolo[4,3‐a]Pyrimidine, and Pyrimido[1,2‐a]Benzimidazole Derivatives Containing the Thiazole Moiety.” Phosphorus, Sulfur, and Silicon and the Related Elements 185, no. 4: 709–718. 10.1080/10426500902922933.

[cbdd70261-bib-0002] Abdulrehman, T. , S. Qadri , Y. Haik , et al. 2024. “Advances in the Targeted Theragnostics of Osteomyelitis Caused by *Staphylococcus aureus* .” Archives of Microbiology 206, no. 7: 288. 10.1007/s00203-024-04015-2. 38834761

[cbdd70261-bib-0003] Abuelizz, H. A. , M. Marzouk , A. Bakhiet , et al. 2021. “In Silico Study and Biological Screening of Benzoquinazolines as Potential Antimicrobial Agents Against Methicillin‐Resistant *Staphylococcus aureus* , Carbapenem‐Resistant Klebsiella pneumoniae, and Fluconazole‐Resistant *Candida albicans* .” Microbial Pathogenesis 160: 105157. 10.1016/j.micpath.2021.105157. 34454024

[cbdd70261-bib-0004] Adams, D. E. , E. M. Shekhtman , E. L. Zechiedrich , M. B. Schmid , and N. R. Cozzarelli . 1992. “The Role of Topoisomerase IV in Partitioning Bacterial Replicons and the Structure of Catenated Intermediates in DNA Replication.” Cell 71, no. 2: 277–288. 10.1016/0092-8674(92)90356-h. 1330320

[cbdd70261-bib-0005] Azam, M. A. , J. Thathan , and S. Jubie . 2015. “Dual Targeting DNA Gyrase B (GyrB) and Topoisomerse IV (ParE) Inhibitors: A Review.” Bioorganic Chemistry 62: 41–63. 10.1016/j.bioorg.2015.07.004. 26232660

[cbdd70261-bib-0006] Banerjee, P. , E. Kemmler , M. Dunkel , and R. Preissner . 2024. “ProTox 3.0: A Webserver for the Prediction of Toxicity of Chemicals.” Nucleic Acids Research 52, no. W1: W513–W520. 10.1093/nar/gkae303. 38647086 PMC11223834

[cbdd70261-bib-0007] Basappa, V. C. , V. H. Kameshwar , K. Kumara , D. K. Achutha , L. N. Krishnappagowda , and A. K. Kariyappa . 2020. “Design and Synthesis of Coumarin‐Triazole Hybrids: Biocompatible Anti‐Diabetic Agents, In Silico Molecular Docking and ADME Screening.” Heliyon 6, no. 10: e05290. 10.1016/j.heliyon.2020.e05290. 33102875 PMC7575805

[cbdd70261-bib-0008] Bauer, M. R. , and M. D. Mackey . 2019. “Electrostatic Complementarity as a Fast and Effective Tool to Optimize Binding and Selectivity of Protein–Ligand Complexes.” Journal of Medicinal Chemistry 62, no. 6: 3036–3050. 10.1021/acs.jmedchem.8b01925. 30807144

[cbdd70261-bib-0009] Bell, E. W. , and Y. Zhang . 2019. “DockRMSD: An Open‐Source Tool for Atom Mapping and RMSD Calculation of Symmetric Molecules Through Graph Isomorphism.” Journal of Cheminformatics 11, no. 1: 40. 10.1186/s13321-019-0362-7. 31175455 PMC6556049

[cbdd70261-bib-0010] Bouvier, G. , N. Evrard‐Todeschi , J.‐P. Girault , and G. Bertho . 2010. “Automatic Clustering of Docking Poses in Virtual Screening Process Using Self‐Organizing Map.” Bioinformatics 26, no. 1: 53–60. 10.1093/bioinformatics/btp623. 19910307

[cbdd70261-bib-0011] Bush, N. G. , K. Evans‐Roberts , and A. Maxwell . 2015. “DNA Topoisomerases.” EcoSal Plus 6, no. 2: 0010‐2014. 10.1128/ecosalplus.esp-0010-2014. PMC11575854 26435256

[cbdd70261-bib-0012] Cheeseright, T. , M. Mackey , S. Rose , and A. Vinter . 2006. “Molecular Field Extrema as Descriptors of Biological Activity: Definition and Validation.” Journal of Chemical Information and Modeling 46, no. 2: 665–676. 10.1021/ci050357s. 16562997

[cbdd70261-bib-0013] Collin, F. , S. Karkare , and A. Maxwell . 2011. “Exploiting Bacterial DNA Gyrase as a Drug Target: Current State and Perspectives.” Applied Microbiology and Biotechnology 92, no. 3: 479–497. 10.1007/s00253-011-3557-z. 21904817 PMC3189412

[cbdd70261-bib-0014] Confreres, A. , and A. Maxwell . 1992. “gyrB Mutations Which Confer Coumarin Resistance Also Affect DNA Supercoiling and ATP Hydrolysis by *Escherichia coli* DNA Gyrase.” Molecular Microbiology 6, no. 12: 1617–1624. 10.1111/j.1365-2958.1992.tb00886.x. 1323022

[cbdd70261-bib-0015] Coumermycin A1 . n.d. “PubChem.” https://pubchem.ncbi.nlm.nih.gov/compound/54675768.

[cbdd70261-bib-0016] Curini, M. , F. Epifano , F. Maltese , M. C. Marcotullio , S. P. Gonzales , and J. C. Rodriguez . 2003. “Synthesis of Collinin, an Antiviral Coumarin.” Australian Journal of Chemistry 56, no. 1: 59–60. 10.1071/ch02177.

[cbdd70261-bib-0017] Daina, A. , O. Michielin , and V. Zoete . 2017. “SwissADME: A Free Web Tool to Evaluate Pharmacokinetics, Drug‐Likeness and Medicinal Chemistry Friendliness of Small Molecules.” Scientific Reports 7, no. 1: 42717. 10.1038/srep42717. 28256516 PMC5335600

[cbdd70261-bib-0018] Docking and Scoring . 2021. “Schrödinger.” https://www.schrodinger.com/life‐science/learn/white‐papers/docking‐and‐scoring/.

[cbdd70261-bib-0019] Ebaid, M. S. , H. Farag , M. Abdelraof , et al. 2025. “Design, Synthesis, and Biological Assessment of a Novel Series of Coumarin‐Tethered Thiazole Derivatives as Potential Antibacterial Agents.” Frontiers in Chemistry 13: 1627186. 10.3389/fchem.2025.1627186. 40979185 PMC12447640

[cbdd70261-bib-0020] Elseginy, S. A. , and M. M. Anwar . 2022. “Pharmacophore‐Based Virtual Screening and Molecular Dynamics Simulation for Identification of a Novel DNA Gyrase B Inhibitor With Benzoxazine Acetamide Scaffold.” ACS Omega 7, no. 1: 1150–1164. 10.1021/acsomega.1c05732. 35036778 PMC8756603

[cbdd70261-bib-0021] Emami, S. , A. Foroumadi , M. A. Faramarzi , and N. Samadi . 2008. “Synthesis and Antibacterial Activity of Quinolone‐Based Compounds Containing a Coumarin Moiety.” Archiv der Pharmazie 341, no. 1: 42–48. 10.1002/ardp.200700090. 18072241

[cbdd70261-bib-0022] Flatman, R. H. , A. Eustaquio , S.‐M. Li , L. Heide , and A. Maxwell . 2006. “Structure‐Activity Relationships of Aminocoumarin‐Type Gyrase and Topoisomerase IV Inhibitors Obtained by Combinatorial Biosynthesis.” Antimicrobial Agents and Chemotherapy 50, no. 4: 1136–1142. 10.1128/AAC.50.4.1136-1142.2006. 16569821 PMC1426943

[cbdd70261-bib-0023] Gellert, M. , M. H. O'Dea , T. Itoh , and J. Tomizawa . 1976. “Novobiocin and Coumermycin Inhibit DNA Supercoiling Catalyzed by DNA Gyrase.” Proceedings of the National Academy of Sciences 73, no. 12: 4474–4478. 10.1073/pnas.73.12.4474. PMC431506 794878

[cbdd70261-bib-0024] Gomaa, H. A. M. , A. M. Atta , M. E. Shaker , et al. 2025. “Design, Synthesis, and Biological Investigation of New Thiazole‐Based Derivatives as Multi‐Targeted Inhibitors Endowed With Antiproliferative, Antioxidant, and Antibacterial Properties.” Frontiers in Chemistry 13: 1595997. 10.3389/fchem.2025.1595997. 40395767 PMC12088962

[cbdd70261-bib-0025] Goth, A. 1945. “The Antibacterial Properties of Dicumarol.” Science 101, no. 2624: 383.a. 10.1126/science.101.2624.383.a. 17780325

[cbdd70261-bib-0026] Guillemin, M. N. , H. M. Miles , and M. I. McDonald . 1986. “Activity of Coumermycin Against Clinical Isolates of Staphylococci.” Antimicrobial Agents and Chemotherapy 29, no. 4: 608–610. 10.1128/aac.29.4.608. 3707109 PMC180451

[cbdd70261-bib-0027] Halip, L. , S. Avram , and C. Neanu . 2021. “The B‐Factor Index for the Binding Site (BFIbs) to Prioritize Crystal Protein Structures for Docking.” Structural Chemistry 32, no. 4: 1693–1699. 10.1007/s11224-021-01751-9.

[cbdd70261-bib-0028] Hardy, C. D. , and N. R. Cozzarelli . 2003. “Alteration of *Escherichia coli* Topoisomerase IV to Novobiocin Resistance.” Antimicrobial Agents and Chemotherapy 47, no. 3: 941–947. 10.1128/AAC.47.3.941-947.2003. 12604525 PMC149342

[cbdd70261-bib-0029] Hayama, R. , and K. J. Marians . 2010. “Physical and Functional Interaction Between the Condensin MukB and the Decatenase Topoisomerase IV in *Escherichia coli* .” Proceedings of the National Academy of Sciences 107, no. 44: 18826–18831. 10.1073/pnas.1008140107. PMC2973858 20696938

[cbdd70261-bib-0030] Hoeksema, H. , J. L. Johnson , and J. W. Hinman . 1955. “Structural Studies on Streptonivicin,1 a New Antibiotic.” Journal of the American Chemical Society 77, no. 24: 6710–6711. 10.1021/ja01629a129.

[cbdd70261-bib-0031] Hooper, D. C. , and G. A. Jacoby . 2016. “Topoisomerase Inhibitors: Fluoroquinolone Mechanisms of Action and Resistance.” Cold Spring Harbor Perspectives in Medicine 6, no. 9: a025320. 10.1101/cshperspect.a025320. 27449972 PMC5008060

[cbdd70261-bib-0032] Insuasty, B. , A. Montoya , D. Becerra , et al. 2013. “Synthesis of Novel Analogs of 2‐Pyrazoline Obtained From [(7‐Chloroquinolin‐4‐Yl)amino]Chalcones and Hydrazine as Potential Antitumor and Antimalarial Agents.” European Journal of Medicinal Chemistry 67: 252–262. 10.1016/j.ejmech.2013.06.049. 23871905

[cbdd70261-bib-0033] Ivanova, A. , O. Mokshyna , and P. Polishchuk . 2024. “StreaMD: The Toolkit for High‐Throughput Molecular Dynamics Simulations.” Journal of Cheminformatics 16, no. 1: 123. 10.1186/s13321-024-00918-w. 39501332 PMC11539841

[cbdd70261-bib-0034] Jain, K. S. , N. Arya , N. N. Inamdar , et al. 2016. “The Chemistry and Bio‐Medicinal Significance of Pyrimidines & Condensed Pyrimidines.” Current Topics in Medicinal Chemistry 16, no. 28: 3133–3174. 10.2174/1568026616666160609100410. 27291985

[cbdd70261-bib-0035] Kato, J. , Y. Nishimura , R. Imamura , H. Niki , S. Hiraga , and H. Suzuki . 1990. “New Topoisomerase Essential for Chromosome Segregation in *E. coli* .” Cell 63, no. 2: 393–404. 10.1016/0092-8674(90)90172-b. 2170028

[cbdd70261-bib-0037] Kuhn, M. , S. Firth‐Clark , P. Tosco , A. S. J. S. Mey , M. Mackey , and J. Michel . 2020. “Assessment of Binding Affinity via Alchemical Free‐Energy Calculations.” Journal of Chemical Information and Modeling 60, no. 6: 3120–3130. 10.1021/acs.jcim.0c00165. 32437145

[cbdd70261-bib-0038] Lafitte, D. , V. Lamour , P. O. Tsvetkov , et al. 2002. “DNA Gyrase Interaction With Coumarin‐Based Inhibitors: The Role of the Hydroxybenzoate Isopentenyl Moiety and the 5′‐Methyl Group of the Noviose.” Biochemistry 41, no. 23: 7217–7223. 10.1021/bi0159837. 12044152

[cbdd70261-bib-0039] Liu, H. , D.‐G. Xia , Z.‐W. Chu , R. Hu , X. Cheng , and X.‐H. Lv . 2020. “Novel Coumarin‐Thiazolyl Ester Derivatives as Potential DNA Gyrase Inhibitors: Design, Synthesis, and Antibacterial Activity.” Bioorganic Chemistry 100: 103907. 10.1016/j.bioorg.2020.103907. 32413631

[cbdd70261-bib-0040] Lynch, B. J. , D. G. Guinee , and J. A. Holden . 1997. “Human DNA Topoisomerase II‐Alpha: A New Marker of Cell Proliferation in Invasive Breast cancer.” Human Pathology 28, no. 10: 1180–1188. 10.1016/S0046-8177(97)90256-2. 9343325

[cbdd70261-bib-0041] Ma, Y.‐X. , C.‐Y. Wang , Y.‐Y. Li , et al. 2020. “Considerations and Caveats in Combating ESKAPE Pathogens Against Nosocomial Infections.” Advanced Science 7, no. 1: 1901872. 10.1002/advs.201901872. 31921562 PMC6947519

[cbdd70261-bib-0042] Magaldi, S. , S. Mata‐Essayag , C. H. de Capriles , et al. 2004. “Well Diffusion for Antifungal Susceptibility Testing.” International Journal of Infectious Diseases 8, no. 1: 39–45. 10.1016/j.ijid.2003.03.002. 14690779

[cbdd70261-bib-0043] Makeneni, S. , D. F. Thieker , and R. J. Woods . 2018. “Applying Pose Clustering and MD Simulations to Eliminate False Positives in Molecular Docking.” Journal of Chemical Information and Modeling 58, no. 3: 605–614. 10.1021/acs.jcim.7b00588. 29431438 PMC6067002

[cbdd70261-bib-0044] Manvar, A. , A. Bavishi , A. Radadiya , et al. 2011. “Diversity Oriented Design of Various Hydrazides and Their in Vitro Evaluation Against *Mycobacterium tuberculosis* H37Rv Strains.” Bioorganic & Medicinal Chemistry Letters 21, no. 16: 4728–4731. 10.1016/j.bmcl.2011.06.074. 21752642

[cbdd70261-bib-0045] Matar, I. K. , Z. A. Muhammad , S. M. Gomha , et al. 2024. “Novel 3‐Substituted Coumarins Inspire a Custom Pharmacology Prediction Pipeline: An Anticancer Discovery Adventure.” Future Medicinal Chemistry 16, no. 17: 1761–1776. 10.1080/17568919.2024.2379232. 39230519 PMC11457655

[cbdd70261-bib-0046] Maxwell, A. 1993. “The Interaction Between Coumarin Drugs and DNA Gyrase.” Molecular Microbiology 9, no. 4: 681–686. 10.1111/j.1365-2958.1993.tb01728.x. 8231802

[cbdd70261-bib-0047] McKie, S. J. , K. C. Neuman , and A. Maxwell . 2021. “DNA Topoisomerases: Advances in Understanding of Cellular Roles and Multi‐Protein Complexes via Structure‐Function Analysis.” BioEssays 43, no. 4: 2000286. 10.1002/bies.202000286. PMC7614492 33480441

[cbdd70261-bib-0048] Meng, E. C. , T. D. Goddard , E. F. Pettersen , et al. 2023. “UCSF ChimeraX: Tools for Structure Building and Analysis.” Protein Science 32, no. 11: e4792. 10.1002/pro.4792. 37774136 PMC10588335

[cbdd70261-bib-0049] Michaeli, D. , B. Meyers , and L. Weinstein . 1969. “In Vitro Studies of the Activity of Coumermycin‐A1 Against Staphylococci Resistant to Methicillin and Cephalothin.” Journal of Infectious Diseases 120, no. 4: 488–490. 10.1093/infdis/120.4.488. 5195766

[cbdd70261-bib-0050] Miller, W. R. , and C. A. Arias . 2024. “ESKAPE Pathogens: Antimicrobial Resistance, Epidemiology, Clinical Impact and Therapeutics.” Nature Reviews Microbiology 22, no. 10: 598–616. 10.1038/s41579-024-01054-w. 38831030 PMC13147291

[cbdd70261-bib-0051] Mosmann, T. 1983. “Rapid Colorimetric Assay for Cellular Growth and Survival: Application to Proliferation and Cytotoxicity Assays.” Journal of Immunological Methods 65, no. 1–2: 55–63. 10.1016/0022-1759(83)90303-4. 6606682

[cbdd70261-bib-0052] Mulani, M. S. , E. E. Kamble , S. N. Kumkar , M. S. Tawre , and K. R. Pardesi . 2019. “Emerging Strategies to Combat ESKAPE Pathogens in the Era of Antimicrobial Resistance: A Review.” Frontiers in Microbiology 10: 539. 10.3389/fmicb.2019.00539. 30988669 PMC6452778

[cbdd70261-bib-0053] Nanayakkara, A. K. , H. W. Boucher , A. Jezek , K. Outterson , and D. E. Greenberg . 2021. “Antibiotic Resistance in the Patient With cancer: Escalating Challenges and Paths Forward.” CA: A Cancer Journal for Clinicians 71, no. 6: 488–504. 10.3322/caac.21697. 34546590

[cbdd70261-bib-0054] Nehra, B. , S. Rulhania , S. Jaswal , B. Kumar , G. Singh , and V. Monga . 2020. “Recent Advancements in the Development of Bioactive Pyrazoline Derivatives.” European Journal of Medicinal Chemistry 205: 112666. 10.1016/j.ejmech.2020.112666. 32795767

[cbdd70261-bib-0055] Nepali, K. , S. Sharma , M. Sharma , P. M. S. Bedi , and K. L. Dhar . 2014. “Rational Approaches, Design Strategies, Structure Activity Relationship and Mechanistic Insights for Anticancer Hybrids.” European Journal of Medicinal Chemistry 77: 422–487. 10.1016/j.ejmech.2014.03.018. 24685980

[cbdd70261-bib-0056] Nielsen, C. F. , T. Zhang , M. Barisic , P. Kalitsis , and D. F. Hudson . 2020. “Topoisomerase IIα Is Essential for Maintenance of Mitotic Chromosome Structure.” Proceedings of the National Academy of Sciences 117, no. 22: 12131–12142. 10.1073/pnas.2001760117. PMC7275761 32414923

[cbdd70261-bib-0057] Novobiocin . n.d. “PubChem.” https://pubchem.ncbi.nlm.nih.gov/compound/54675769.

[cbdd70261-bib-0058] O'Boyle, N. M. , M. Banck , C. A. James , C. Morley , T. Vandermeersch , and G. R. Hutchison . 2011. “Open Babel: An Open Chemical Toolbox.” Journal of Cheminformatics 3, no. 1: 33. 10.1186/1758-2946-3-33. 21982300 PMC3198950

[cbdd70261-bib-0059] Ostrov, D. A. , J. A. Hernández Prada , P. E. Corsino , K. A. Finton , N. Le , and T. C. Rowe . 2007. “Discovery of Novel DNA Gyrase Inhibitors by High‐Throughput Virtual Screening.” Antimicrobial Agents and Chemotherapy 51, no. 10: 3688–3698. 10.1128/aac.00392-07. 17682095 PMC2043263

[cbdd70261-bib-0060] Patel, M. , N. Pandey , J. Timaniya , et al. 2021. “Coumarin–Carbazole Based Functionalized Pyrazolines: Synthesis, Characterization, Anticancer Investigation and Molecular Docking.” RSC Advances 11, no. 44: 27627–27644. 10.1039/D1RA03970A. 35480680 PMC9037808

[cbdd70261-bib-0061] Pommier, Y. , E. Leo , H. Zhang , and C. Marchand . 2010. “DNA Topoisomerases and Their Poisoning by Anticancer and Antibacterial Drugs.” Chemistry & Biology 17, no. 5: 421–433. 10.1016/j.chembiol.2010.04.012. 20534341 PMC7316379

[cbdd70261-bib-0062] Pommier, Y. , A. Nussenzweig , S. Takeda , and C. Austin . 2022. “Human Topoisomerases and Their Roles in Genome Stability and Organization.” Nature Reviews Molecular Cell Biology 23, no. 6: 407–427. 10.1038/s41580-022-00452-3. 35228717 PMC8883456

[cbdd70261-bib-0063] PyMOL.org . n.d. “PyMOL.” https://pymol.org/2/.

[cbdd70261-bib-0064] Qu, D. , Z. Hou , J. Li , et al. 2020. “A New Coumarin Compound DCH Combats Methicillin‐Resistant *Staphylococcus aureus* Biofilm by Targeting Arginine Repressor.” Science Advances 6, no. 30: eaay9597. 10.1126/sciadv.aay9597. 32832655 PMC7439407

[cbdd70261-bib-0065] Radwan, I. T. , I. M. El‐Sherbiny , A. M. Selim , and N. H. Metwally . 2024. “Design, Synthesis of Some Novel Coumarins and Their Nanoformulations Into Lipid‐Chitosan Nanocapsule as Unique Antimicrobial Agents.” Scientific Reports 14, no. 1: 30598. 10.1038/s41598-024-79861-7. 39715779 PMC11666591

[cbdd70261-bib-0066] Rajakumari, K. , K. Aravind , M. Balamugundhan , et al. 2024. “Comprehensive Review of DNA Gyrase as Enzymatic Target for Drug Discovery and Development.” European Journal of Medicinal Chemistry Reports 12: 100233. 10.1016/j.ejmcr.2024.100233.

[cbdd70261-bib-0067] RCSB Protein Data Bank . n.d. “RCSB PDB.” https://www.rcsb.org/.

[cbdd70261-bib-0068] Reece, R. J. , and A. Maxwell . 1991. “DNA Gyrase: Structure and Function.” Critical Reviews in Biochemistry and Molecular Biology 26, no. 3–4: 335–375. 10.3109/10409239109114072. 1657531

[cbdd70261-bib-0069] Reen, F. J. , J. A. Gutiérrez‐Barranquero , M. L. Parages , and F. O'Gara . 2018. “Coumarin: A Novel Player in Microbial Quorum Sensing and Biofilm Formation Inhibition.” Applied Microbiology and Biotechnology 102, no. 5: 2063–2073. 10.1007/s00253-018-8787-x. 29392389 PMC5814477

[cbdd70261-bib-0036] Rehman, S. U. , R. Khan , K. A. Bhat , A. F. Raja , A. S. Shawl , and M. S. Alam . 2010. “Isolation, Characterisation and Antibacterial Activity Studies of Coumarins From Rhododendron Lepidotum Wall. Ex G. Don, Ericaceae.” Revista Brasileira de Farmacognosia 20: 886–890. 10.1590/S0102-695X2010005000037.

[cbdd70261-bib-0070] Sairam, K. V. , B. M. Gurupadayya , B. I. Vishwanathan , R. S. Chandan , and D. K. Nagesha . 2016. “Cytotoxicity Studies of Coumarin Analogs: Design, Synthesis and Biological Activity.” RSC Advances 6, no. 101: 98816–98828. 10.1039/C6RA22466K.

[cbdd70261-bib-0071] Sander, T. , J. Freyss , M. V. Korff , and C. Rufener . 2015. “DataWarrior: An Open‐Source Program for Chemistry Aware Data Visualization and Analysis.” Journal of Chemical Information and Modeling 55, no. 2: 460–473. 10.1021/ci500588j. 25558886

[cbdd70261-bib-0073] Skok, Ž. , M. Durcik , D. Gramec Skledar , et al. 2020. “Discovery of New ATP‐Competitive Inhibitors of Human DNA Topoisomerase IIα Through Screening of Bacterial Topoisomerase Inhibitors.” Bioorganic Chemistry 102: 104049. 10.1016/j.bioorg.2020.104049. 32688116

[cbdd70261-bib-0074] Sugino, A. , N. P. Higgins , P. O. Brown , C. L. Peebles , and N. R. Cozzarelli . 1978. “Energy Coupling in DNA Gyrase and the Mechanism of Action of Novobiocin.” Proceedings of the National Academy of Sciences 75, no. 10: 4838–4842. 10.1073/pnas.75.10.4838. PMC336216 368801

[cbdd70261-bib-0075] Tanimoto, T. T. 1958. An Elementary Mathematical Theory of Classification and Prediction. International Business Machines Corporation.

[cbdd70261-bib-0076] Vanden Broeck, A. , C. Lotz , R. Drillien , L. Haas , C. Bedez , and V. Lamour . 2021. “Structural Basis for Allosteric Regulation of Human Topoisomerase IIα.” Nature Communications 12, no. 1: 2962. 10.1038/s41467-021-23136-6. PMC8137924 34016969

[cbdd70261-bib-0077] Vanden Broeck, A. , A. G. McEwen , Y. Chebaro , N. Potier , and V. Lamour . 2019. “Structural Basis for DNA Gyrase Interaction With Coumermycin A1.” Journal of Medicinal Chemistry 62, no. 8: 4225–4231. 10.1021/acs.jmedchem.8b01928. 30920824

[cbdd70261-bib-0078] von Korff, M. , J. Freyss , and T. Sander . 2008. “Flexophore, a New Versatile 3D Pharmacophore Descriptor That Considers Molecular Flexibility.” Journal of Chemical Information and Modeling 48, no. 4: 797–810. 10.1021/ci700359j. 18393490

[cbdd70261-bib-0079] Wahab, A. , F. Nadeem , U. Salar , et al. 2024. “Coumarin Derivatives as New Anti‐Biofilm Agents Against *Staphylococcus aureus* .” PLoS One 19, no. 9: e0307439. 10.1371/journal.pone.0307439. 39298451 PMC11412489

[cbdd70261-bib-0080] Wang, E. , H. Sun , J. Wang , et al. 2019. “End‐Point Binding Free Energy Calculation With MM/PBSA and MM/GBSA: Strategies and Applications in Drug Design.” Chemical Reviews 119, no. 16: 9478–9508. 10.1021/acs.chemrev.9b00055. 31244000

[cbdd70261-bib-0081] Wang, X. , D. Chen , S. Yu , et al. 2016. “Synthesis and Evaluation of Biological and Antitumor Activities of Tetrahydrobenzothieno[2,3‐d]Pyrimidine Derivatives as Novel Inhibitors of FGFR1.” Chemical Biology & Drug Design 87, no. 4: 499–507. 10.1111/cbdd.12687. 26575787

[cbdd70261-bib-0082] Waterhouse, A. M. , J. B. Procter , D. M. A. Martin , M. Clamp , and G. J. Barton . 2009. “Jalview Version 2—A Multiple Sequence Alignment Editor and Analysis Workbench.” Bioinformatics 25, no. 9: 1189–1191. 10.1093/bioinformatics/btp033. 19151095 PMC2672624

[cbdd70261-bib-0083] Watson, J. D. , and F. H. C. Crick . 1953. “Genetical Implications of the Structure of Deoxyribonucleic Acid.” Nature 171, no. 4361: 964–967. 10.1038/171964b0. 13063483

[cbdd70261-bib-0084] Wei, N.‐N. , A. Hamza , C. Hao , Z. Xiu , and C.‐G. Zhan . 2013. “Microscopic Modes and Free Energies for Topoisomerase I‐DNA Covalent Complex Binding With Non‐Camptothecin Inhibitors by Molecular Docking and Dynamics Simulations.” Theoretical Chemistry Accounts 132, no. 8: 1379. 10.1007/s00214-013-1379-z. PMC3867144 24363608

[cbdd70261-bib-0085] Wendorff, T. J. , B. H. Schmidt , P. Heslop , C. A. Austin , and J. M. Berger . 2012. “The Structure of DNA‐Bound Human Topoisomerase II Alpha: Conformational Mechanisms for Coordinating Inter‐Subunit Interactions With DNA Cleavage.” Journal of Molecular Biology 424, no. 3–4: 109–124. 10.1016/j.jmb.2012.07.014. 22841979 PMC3584591

[cbdd70261-bib-0086] Willett, P. , J. M. Barnard , and G. M. Downs . 1998. “Chemical Similarity Searching.” Journal of Chemical Information and Computer Sciences 38, no. 6: 983–996. 10.1021/ci9800211.

[cbdd70261-bib-0072] Yadav, C. S. , I. Azad , A. Rahman Khan , et al. 2024. “Recent Advances in the Synthesis of Pyrazoline Derivatives From Chalcones as Potent Pharmacological Agents: A Comprehensive Review.” Results in Chemistry 7: 101326. 10.1016/j.rechem.2024.101326.

[cbdd70261-bib-0087] Yang, M. , Z. Bo , T. Xu , B. Xu , D. Wang , and H. Zheng . 2023. “Uni‐GBSA: An Open‐Source and Web‐Based Automatic Workflow to Perform MM/GB(PB)SA Calculations for Virtual Screening.” Briefings in Bioinformatics 24, no. 4: bbad218. 10.1093/bib/bbad218. 37328705

[cbdd70261-bib-0088] Yildirim, M. , S. Poyraz , and M. Ersatir . 2023. “Recent Advances on Biologically Active Coumarin‐Based Hybrid Compounds.” Medicinal Chemistry Research 32, no. 4: 617–642. 10.1007/s00044-023-03025-x.

[cbdd70261-bib-0089] Zhang, Y. , B. Zou , Z. Chen , et al. 2011. “Synthesis and Antioxidant Activities of Novel 4‐Schiff Base‐7‐Benzyloxy‐Coumarin Derivatives.” Bioorganic & Medicinal Chemistry Letters 21, no. 22: 6811–6815. 10.1016/j.bmcl.2011.09.029. 21978674

[cbdd70261-bib-0090] Zhen, X. , C. S. Lundborg , X. Sun , X. Hu , and H. Dong . 2019. “Economic Burden of Antibiotic Resistance in ESKAPE Organisms: A Systematic Review.” Antimicrobial Resistance & Infection Control 8, no. 1: 137. 10.1186/s13756-019-0590-7. 31417673 PMC6692939

[cbdd70261-bib-0091] 李明凯, 明. , 艳. 刘艳 , 梦. 徐梦娴 , et al. 2018. “China Patent No. CN108635346A.” https://patents.google.com/patent/CN108635346A/en.

[cbdd70261-bib-0092] 李明凯, 明. , 晓. 罗晓星 , 征. 侯征 , et al. 2013. “China Patent No. CN103333148A.” https://patents.google.com/patent/CN103333148A/en.

