# Supplementary material for: Coumarin‐Augmented Thiazole Hybrids as Dual Anticancer and Antibacterial Agents
Source: Chem Biol Drug Des. 2026 Feb 20;107(2):e70261. doi: 10.1111/cbdd.70261 (PMC12923669; doi:10.1111/cbdd.70261)
Supplement: Supplementary file 1 — Data S1: cbdd70261‐sup‐0001‐DataS1.docx. [file CBDD-107-e70261-s004.docx]

**Supporting Information for:**

Coumarin-Augmented Thiazole Hybrids as Dual Anticancer and Antibacterial Agents

Islam K. Matar^1,2*^[
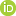
](https://orcid.org/0000-0001-5997-2574), Magdi E. A. Zaki^3^[
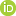
](https://orcid.org/0000-0002-5643-9202), Zeinab A. Muhammad^4^[
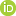
](https://orcid.org/0000-0003-1711-8497), Dahlia A. Awwad^5^[
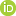
](https://orcid.org/0009-0001-0118-5284),

Sami A. Al-Hussain^3^[
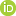
](https://orcid.org/0000-0002-4305-934X), Chérif F. Matta^1,2^[
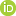
](https://orcid.org/0000-0001-8397-5353), Refaie M. Kassab^6^**[
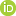
](https://orcid.org/0000-0002-8304-736X)**

^1^Department of Chemistry, Saint Mary's University, 923 Robie Street, B3H 3C3 Halifax, NS, Canada

^2^Department of Chemistry and Physics, Mount Saint Vincent University, 166 Bedford Highway, B3M 2J6 Halifax, NS, Canada

^3^Department of Chemistry, Faculty of Science, Imam Mohammad Ibn Saud Islamic University (IMSIU), Riyadh 11623, KSA

^4^Department of Pharmaceutical Chemistry, Egyptian Drug Authority (EDA), Giza 12311, Egypt

^5^University of Science and Technology (UST), Zewail City of Science and Technology, Giza, Egypt

^6^Department of Chemistry, Faculty of Science, Cairo University, Giza 12613, Egypt

* E-mail: [islam.matar@smu.ca](mailto:islam.matar@smu.ca)

**Figures**


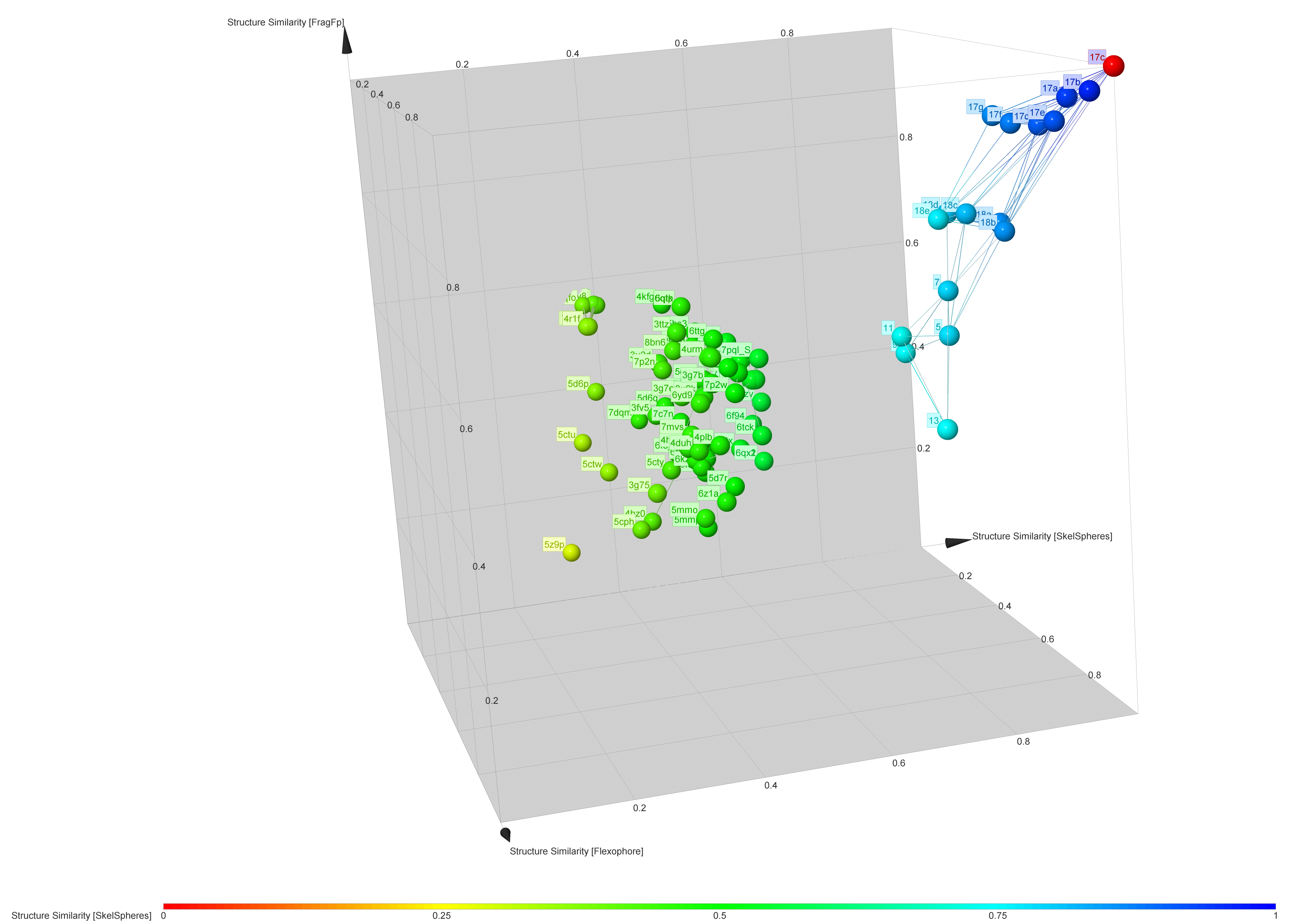


**Figure S1.** 3D chemical similarity map of the 95-compound dataset based on FragFp, SkelSpheres, and Flexophore descriptors.


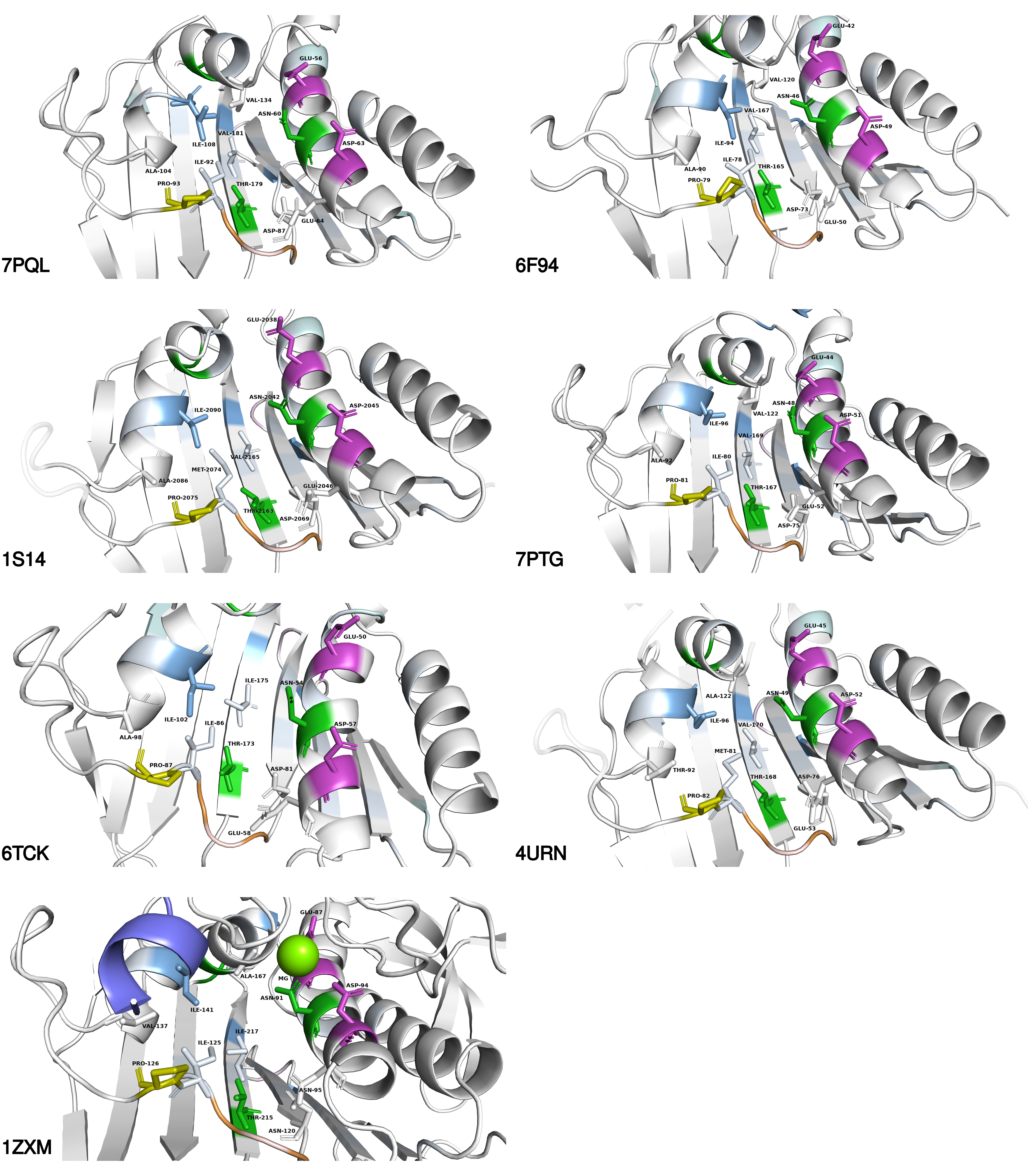


**Figure S2.** Conservation analysis montage showing the aligned ATPase domains across the selected topoisomerases, with pharmacophoric residues depicted as sticks.


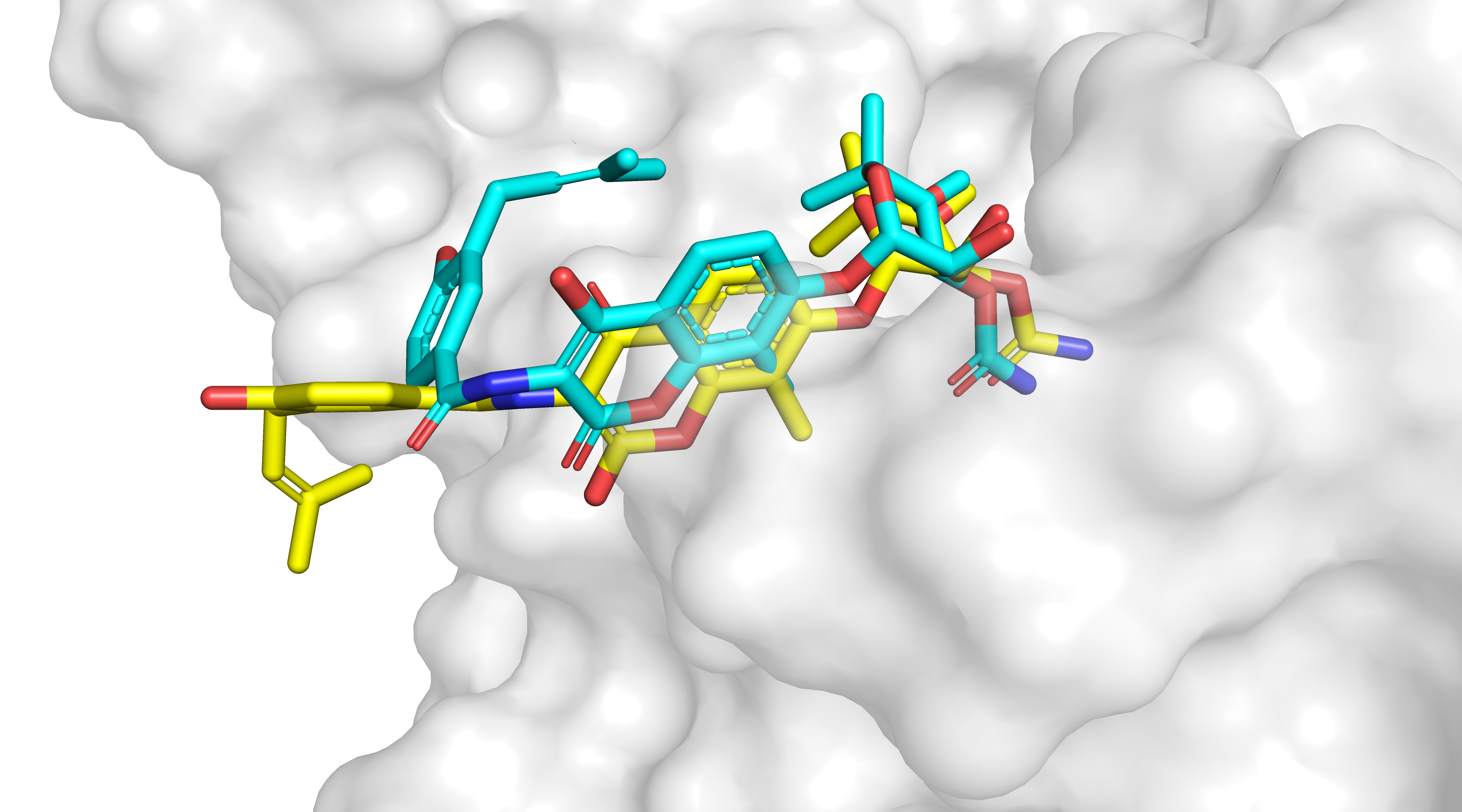


**Figure S3.** Redocking pose deviations for E. coli topoisomerase IV (1S14), showing flexible ligand tail regions; Reference pose shown in cyan.


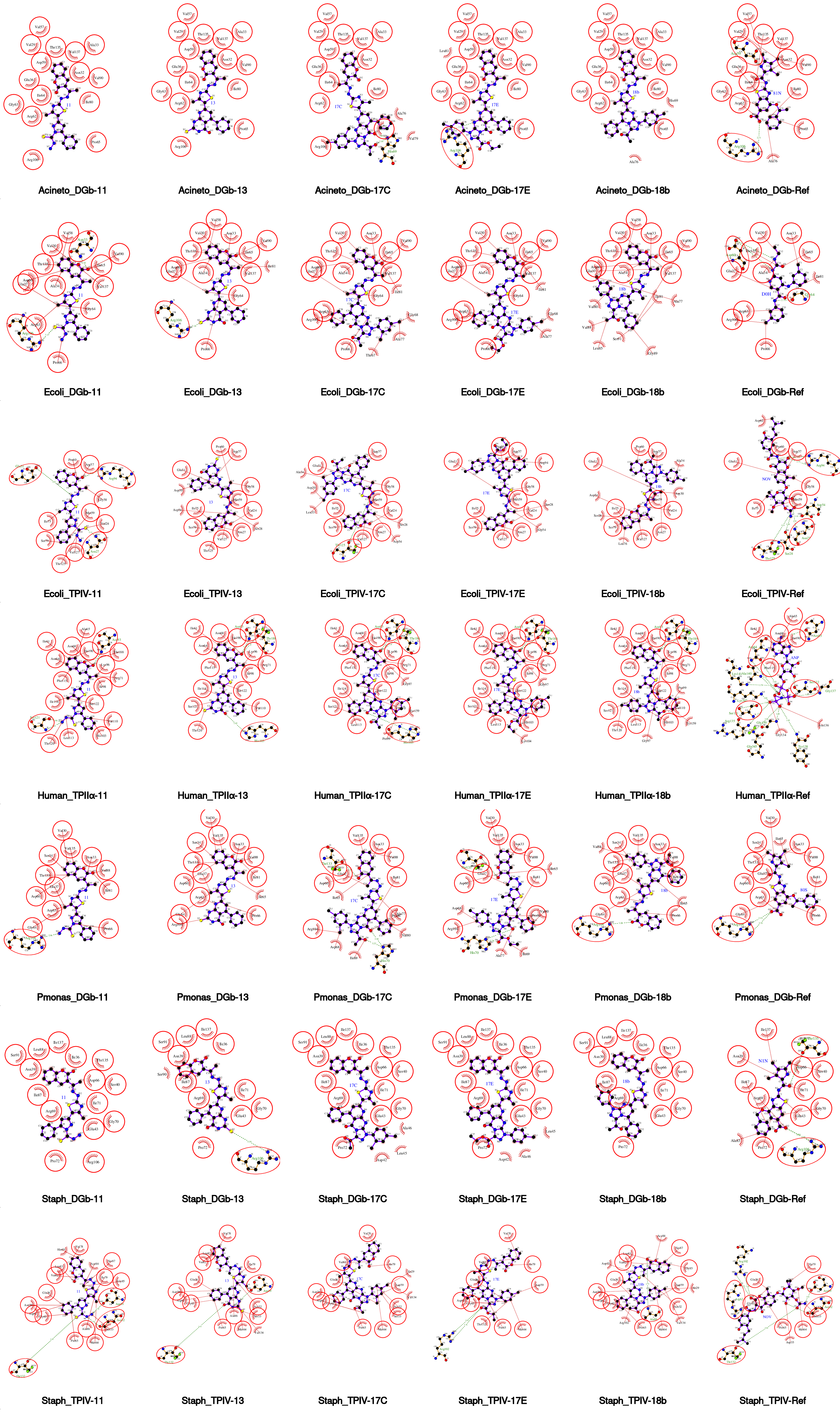


**Figure S4.** 2D protein-ligand interaction diagrams for all prioritized coumarins and reference ligands across seven targets.


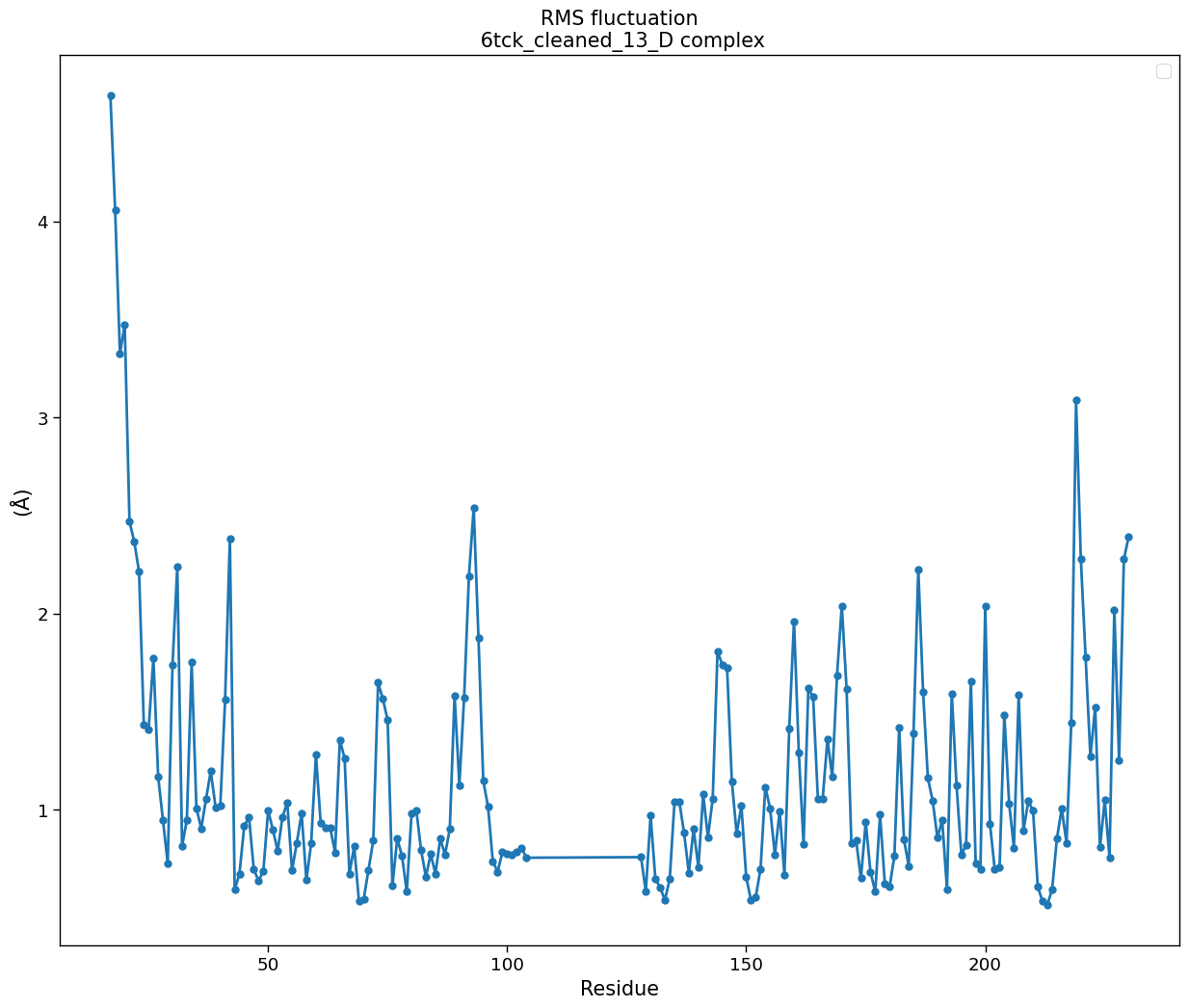


**Figure S5.** Per-residue root mean square fluctuation (RMSF) plot from the MD simulation of compound 13 bound to 6TCK.


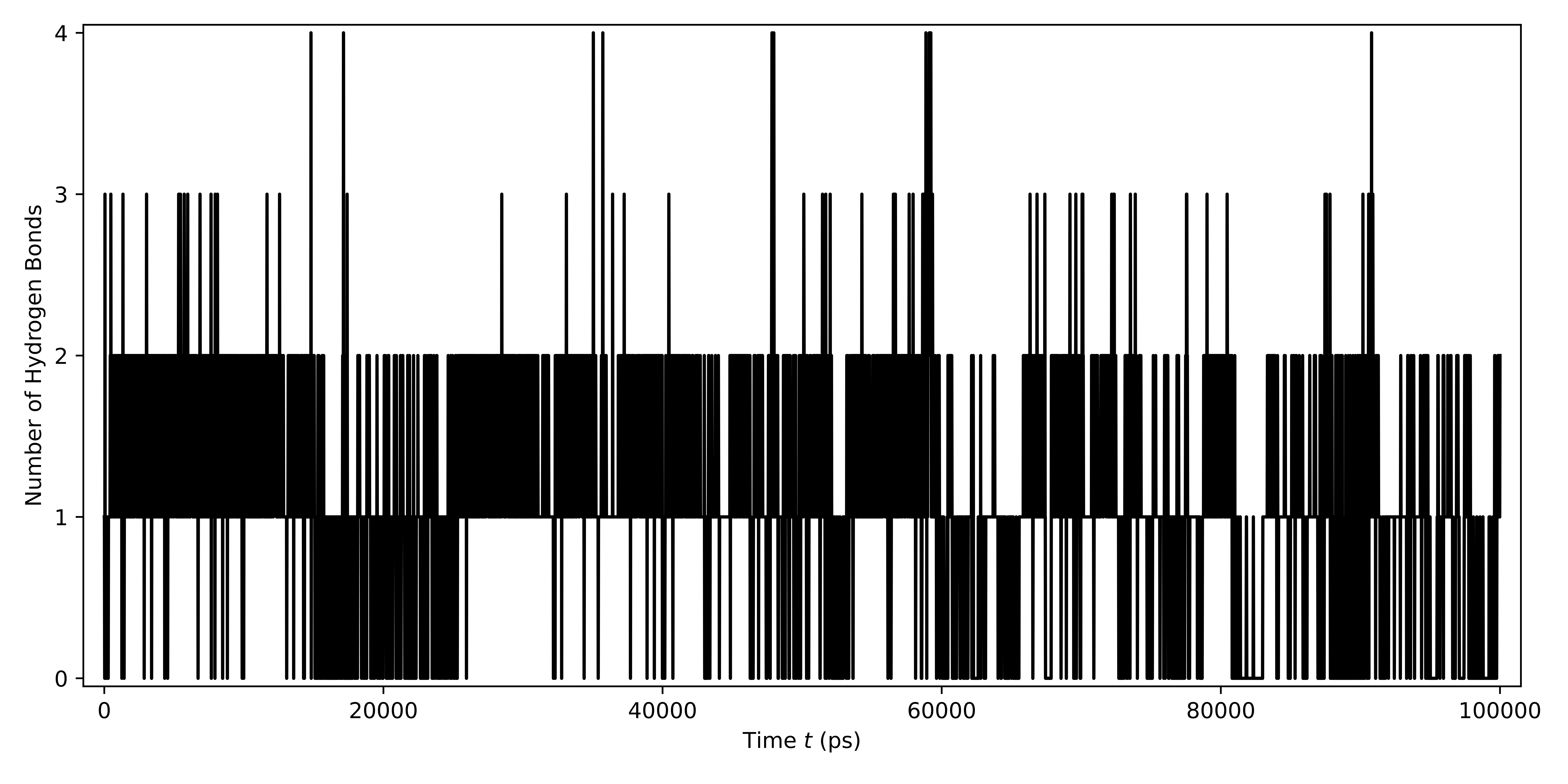


**Figure S6.** Time evolution of the number of hydrogen bonds formed between compound 13 and 6TCK during the 100 ns molecular dynamics (MD) simulation.


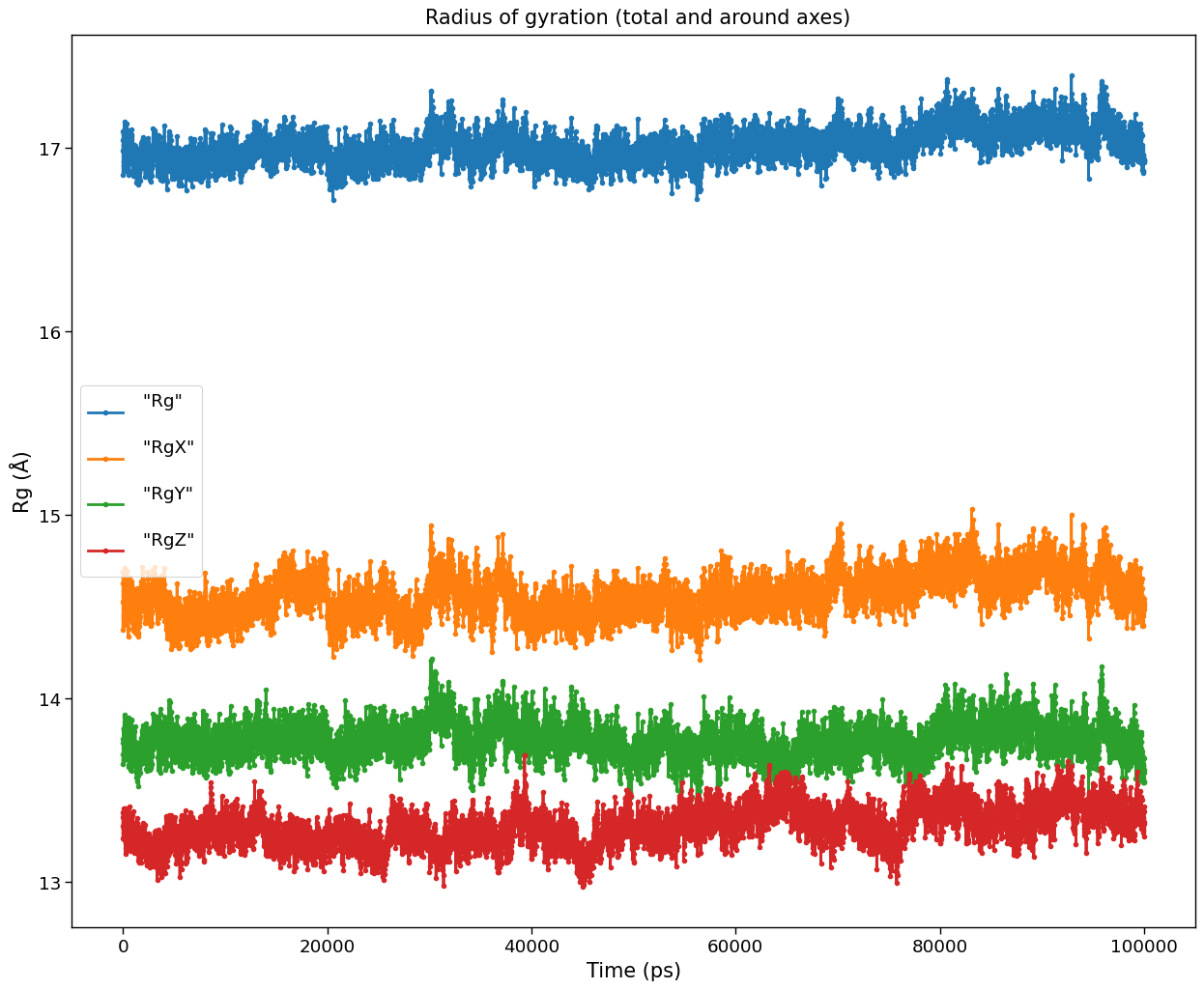


**Figure S7.** Radius of gyration (Rg) of the 6TCK-compound 13 complex over the 100 ns MD simulation. The plot shows the total Rg as well as components along the X, Y, and Z axes, indicating structural compactness and anisotropy of the complex throughout the trajectory.


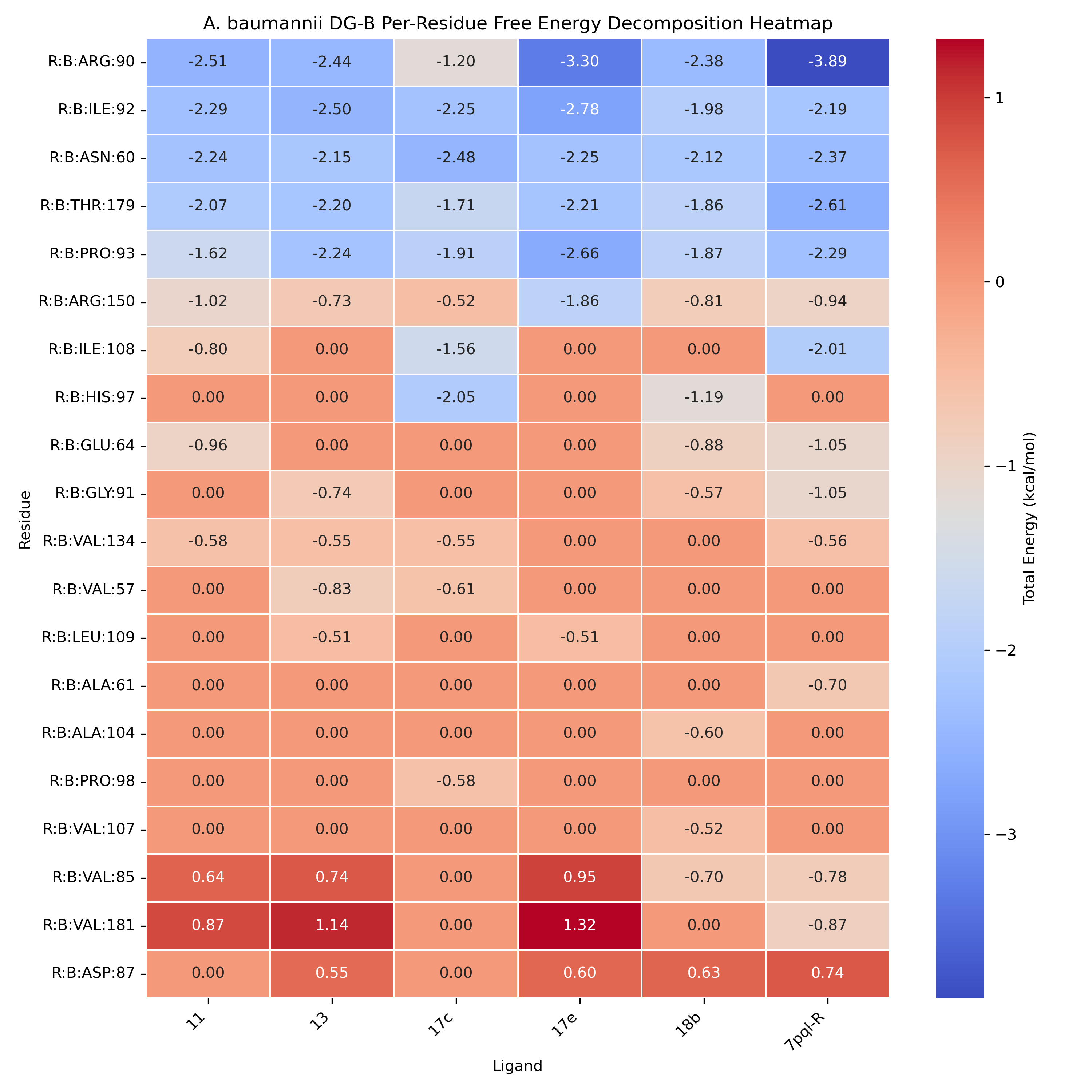


**Figure S8A.** MM/GBSA per-residue free energy decomposition heatmap for A. baumannii DG-B.


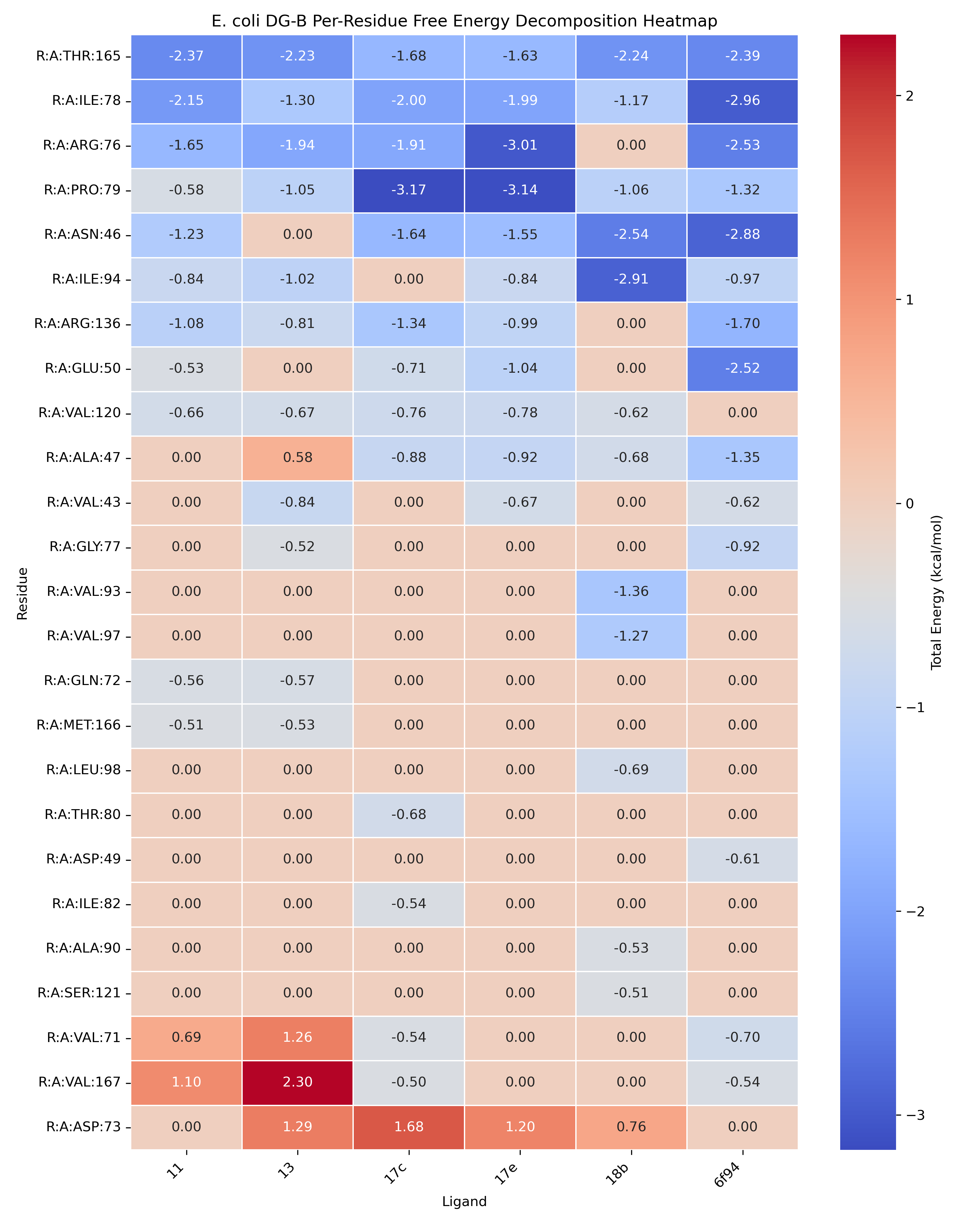


**Figure S8B.** MM/GBSA per-residue free energy decomposition heatmap for E. coli DG-B.


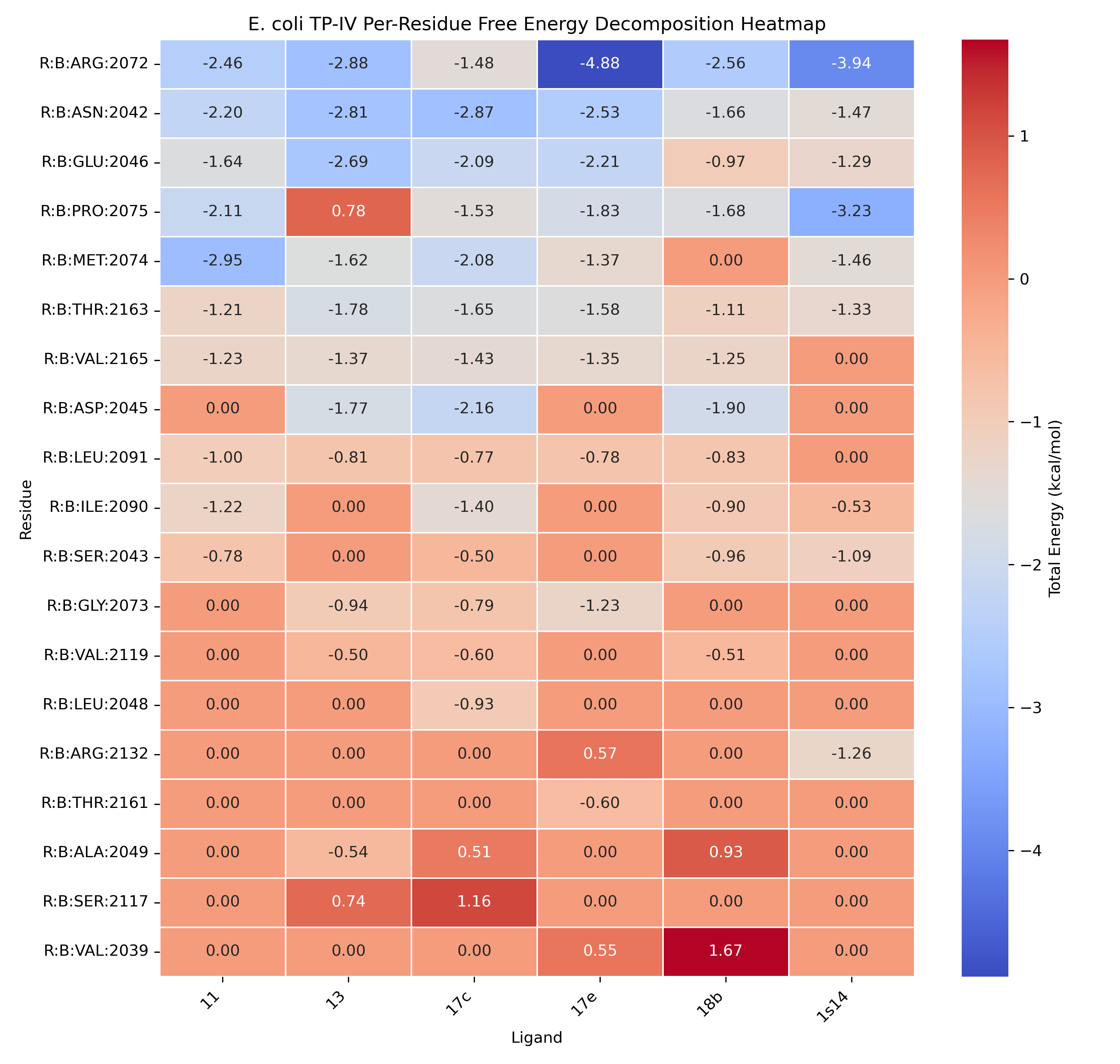


**Figure S8C.** MM/GBSA per-residue free energy decomposition heatmap for E. coli TP-IV.


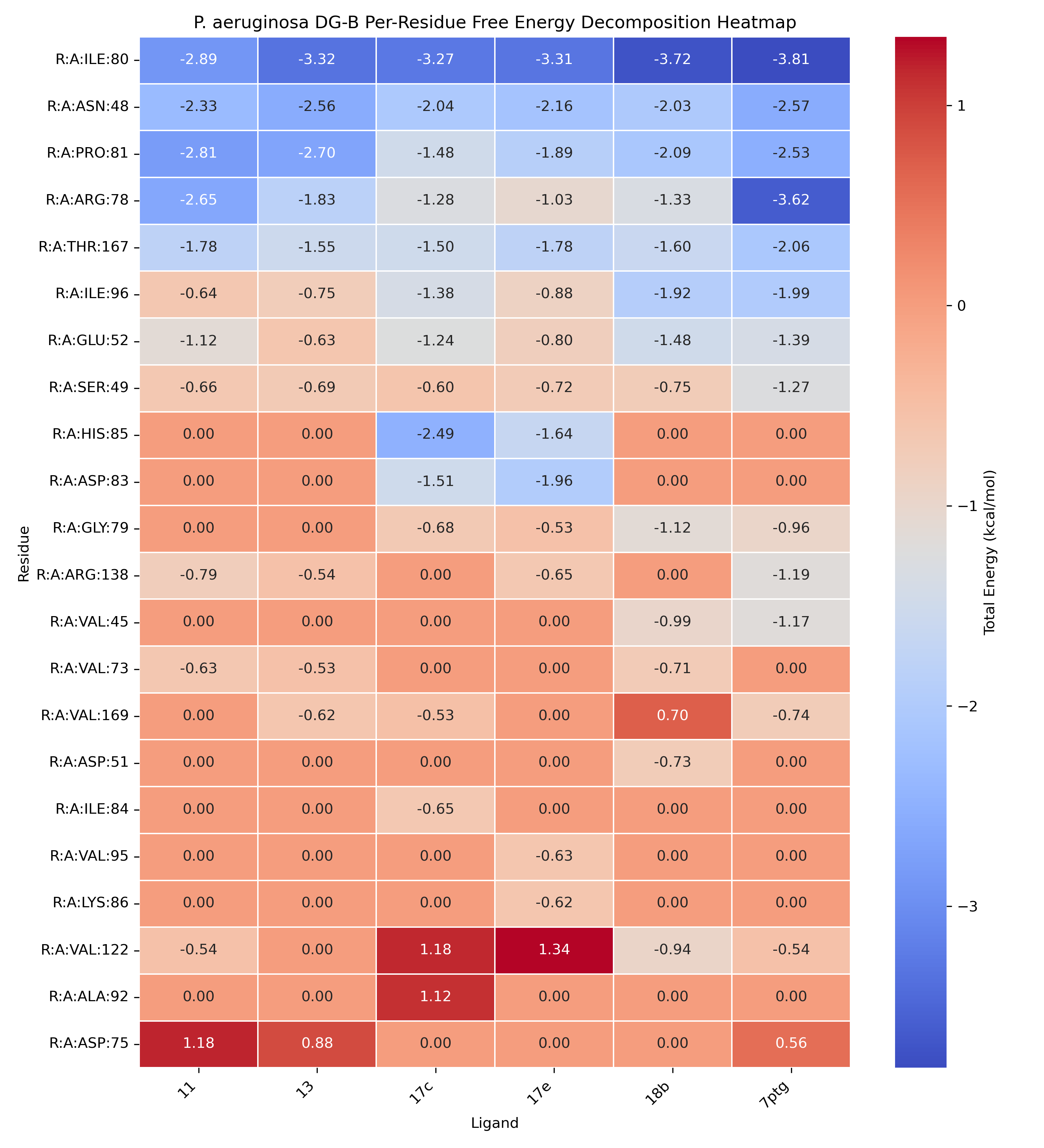


**Figure S8D.** MM/GBSA per-residue free energy decomposition heatmap for P. aeruginosa DG-B.


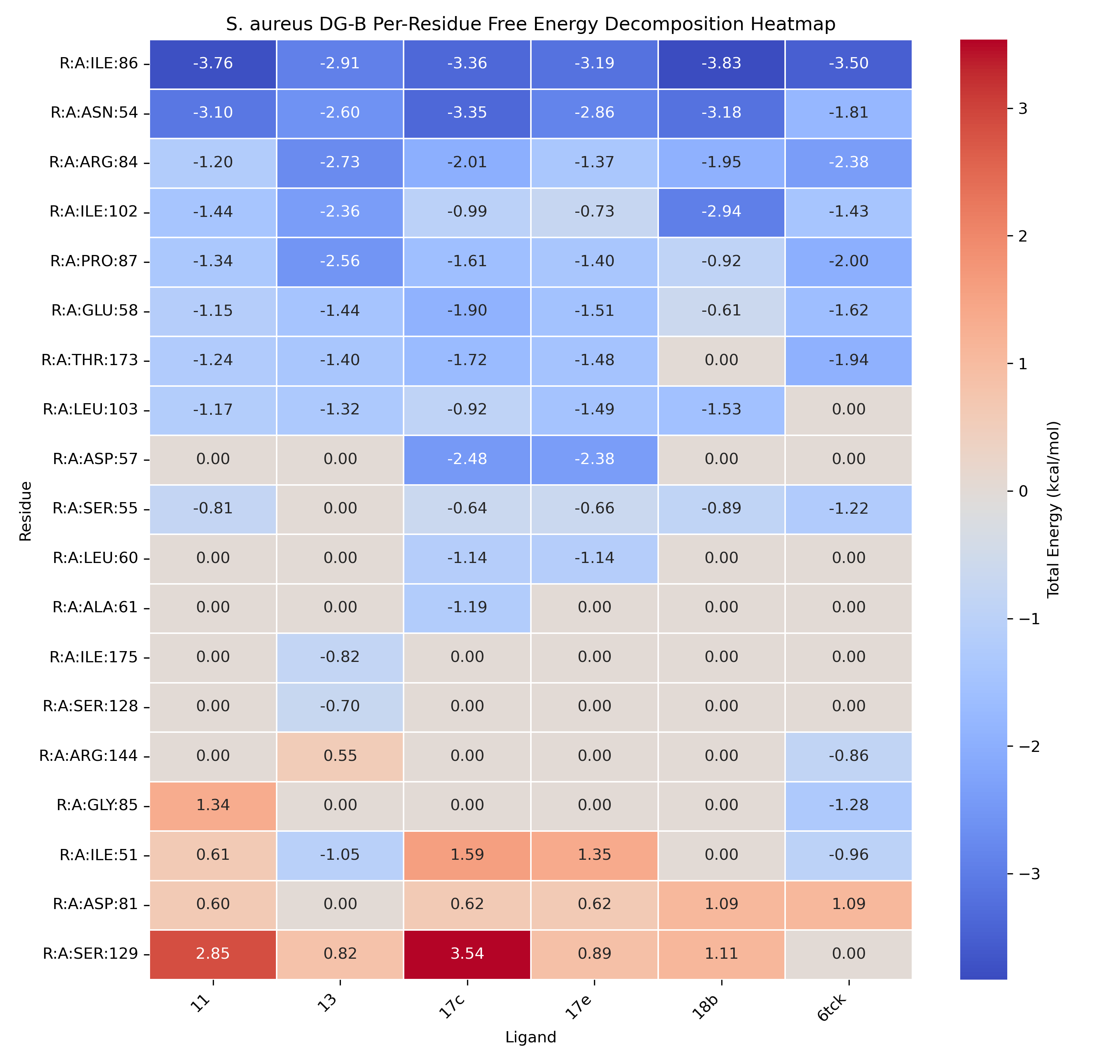


**Figure S8E.** MM/GBSA per-residue free energy decomposition heatmap for S. aureus DG-B.


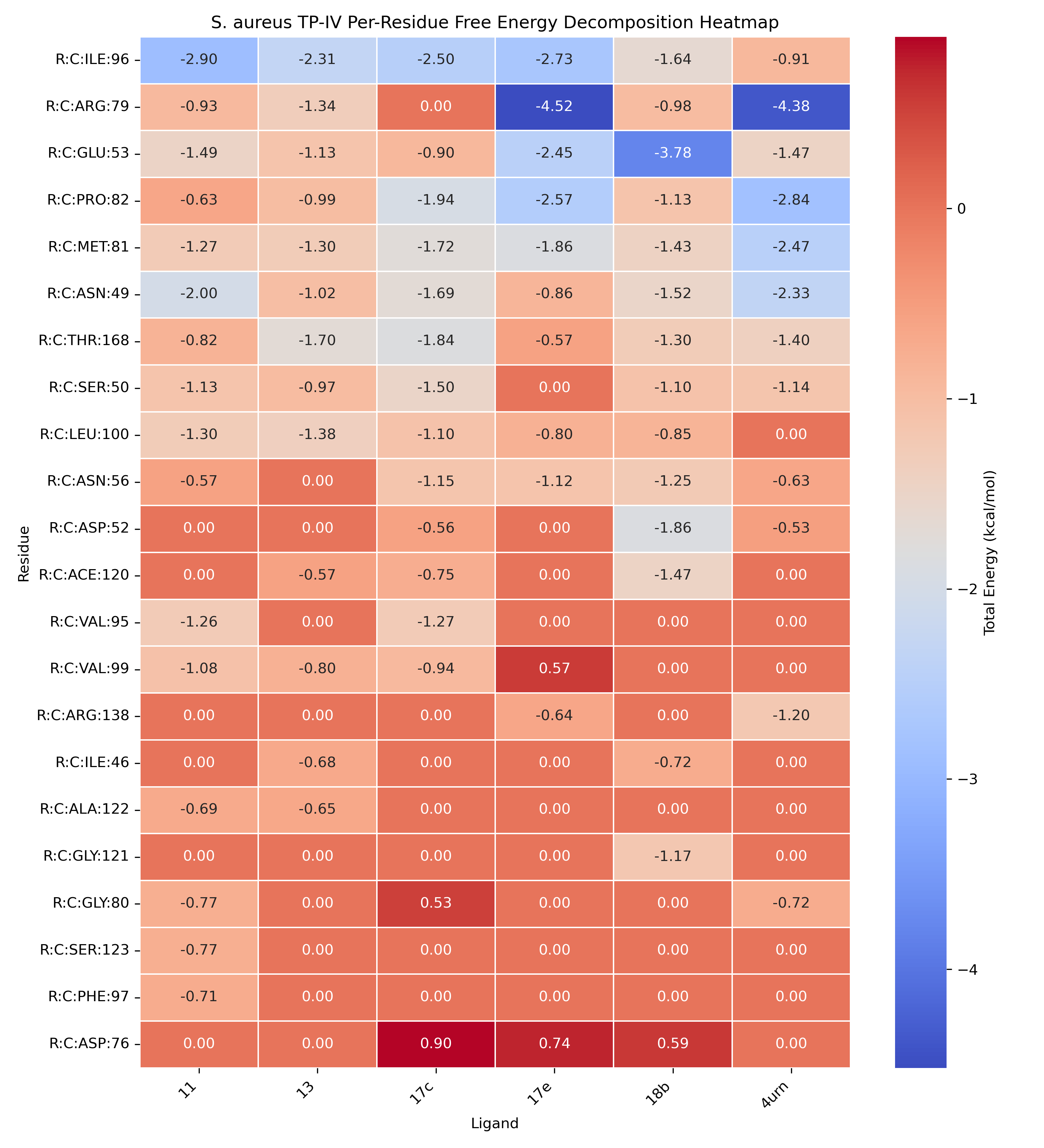


**Figure S8F.** MM/GBSA per-residue free energy decomposition heatmap for S. aureus TP-IV.


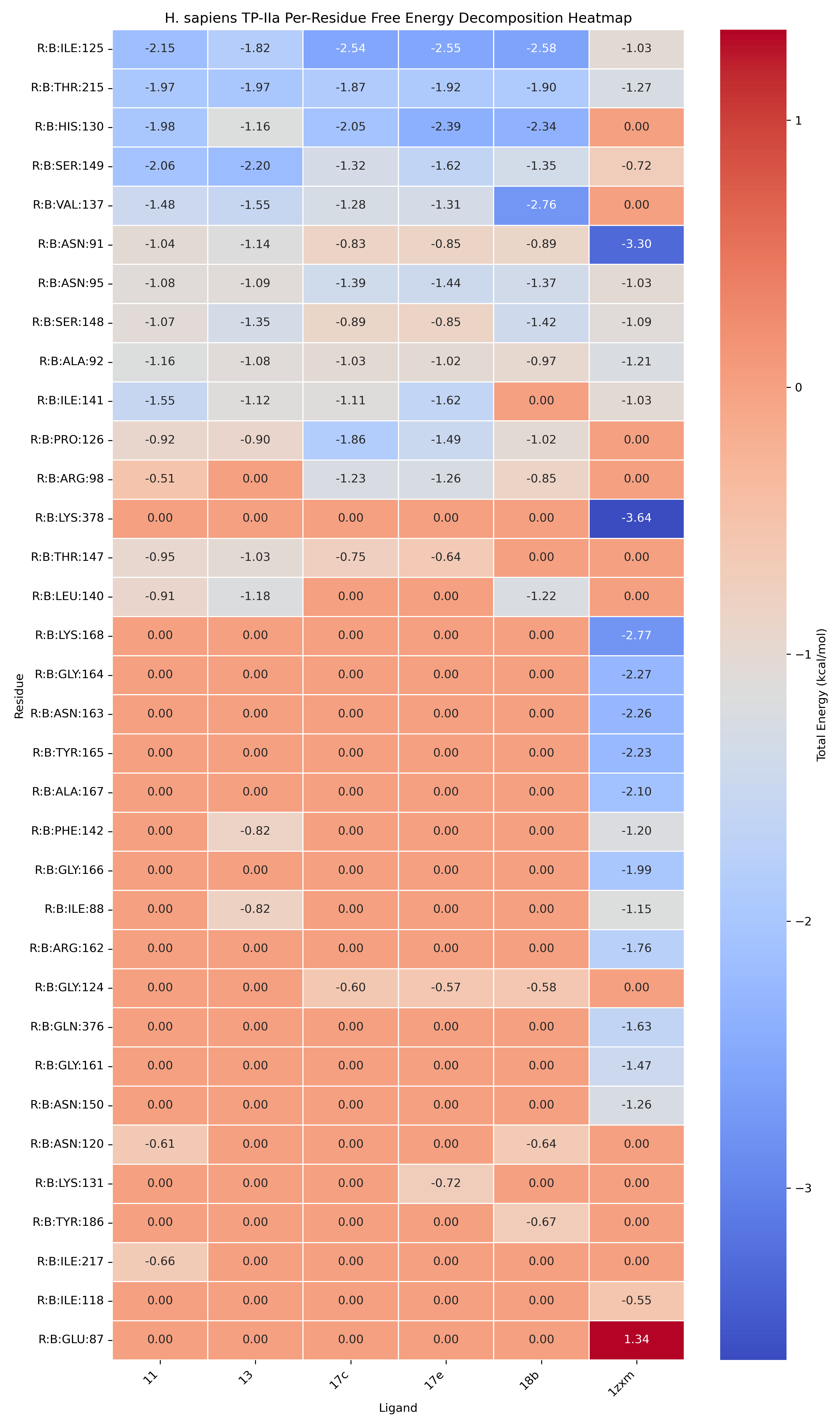


**Figure S8G.** MM/GBSA per-residue free energy decomposition heatmap for human topoisomerase IIα.


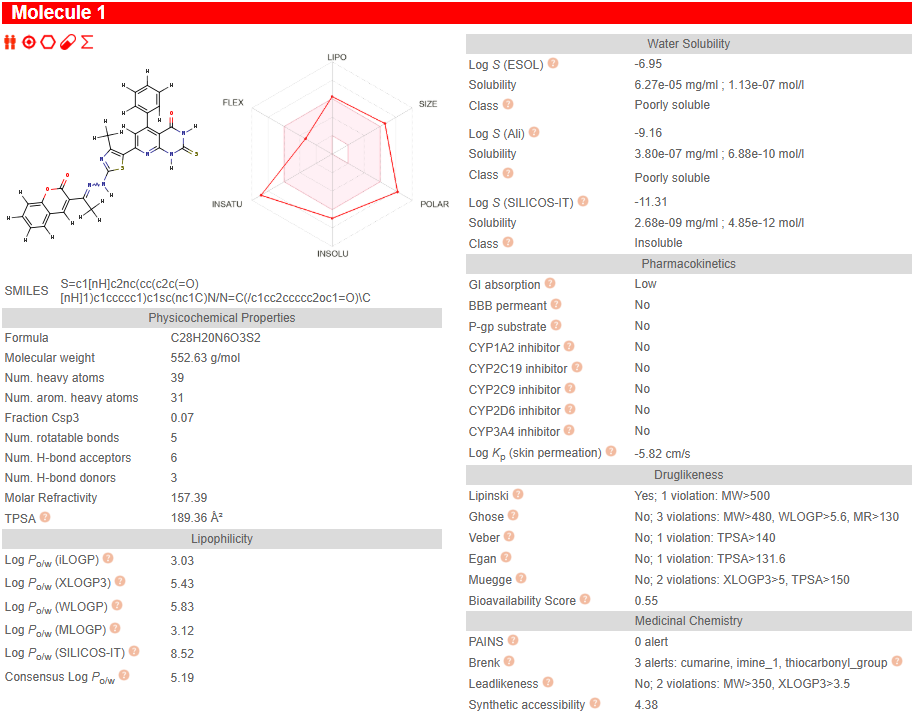


**Figure S9.** SwissADME summary report for compound 13, showing physicochemical properties, pharmacokinetics, drug-likeness, and medicinal chemistry alerts.


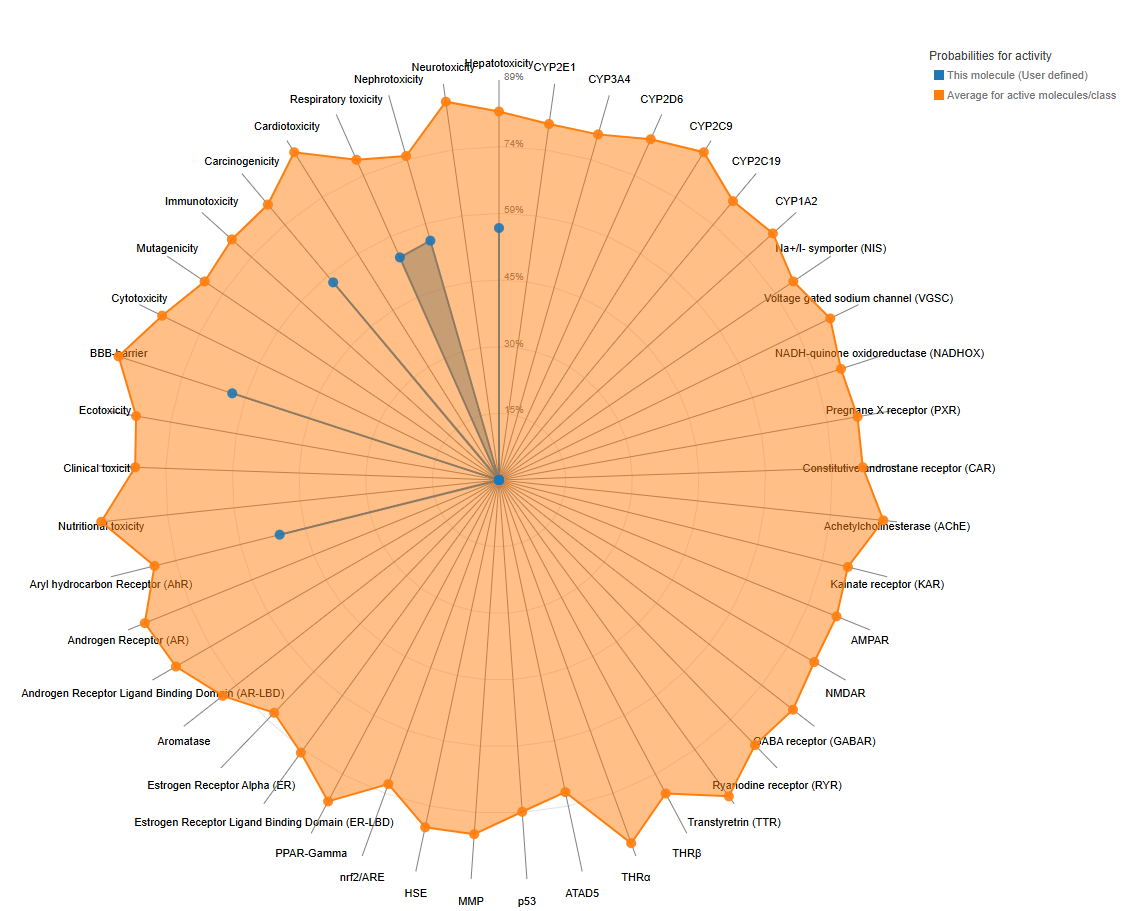


**Figure S10A.** ProTox-3.0 toxicity radar chart for compound 13.


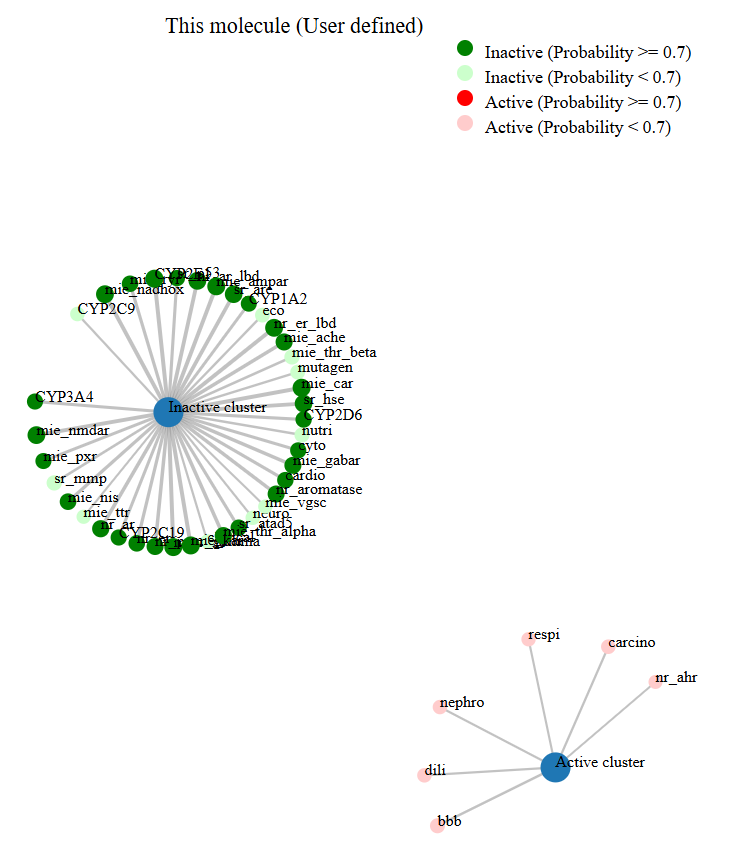


**Figure S10B.** ProTox-3.0 toxicity network chart for compound 13.

**Tables**

| Sample Code | IC_50_ values (µg/mL) |
| --- | --- |
| 5 | 106.0 ± 6.21 |
| 7 | 68.6 ± 5.08 |
| 9 | 100 ± 4.69 |
| 11 | 47.5 ± 2.13 |
| 13 | 26.8 ± 0.97 |
| 17a | 239.1± 10.17 |
| 17b | 285.2 ± 14.28 |
| 17c | 91.7 ± 5.93 |
| 17d | 88.4 ± 4.87 |
| 17e | 54.4 ± 3.06 |
| 17f | 224.8 ± 9.37 |
| 17g | 239.3 ± 10.23 |
| 18a | 70.6 ± 4.08 |
| 18b | 37.02 ± 2.13 |
| 18c | 59.1± 2.91 |
| 18d | 204.3 ± 6.43 |
| 18e | 58.9 ± 2.71 |
| Doxorubicin | 10.59 ± 1.03 |

**Table S1.** IC₅₀ values (µg/mL) of all synthesized coumarin derivatives against HeLa cervical cancer cells.

| **Compounds** | **Methicillin-sensitive *Staphylococcus aureus* (MSSA)** | **Methicillin-resistant *Staphylococcus aureus* (MRSA)** | **Vancomycin-resistant *Staphylococcus aureus* (VRSA)** |
| --- | --- | --- | --- |
| 5 | 7.81 | 31.25 | NA |
| 7 | 7.81 | 15.63 | NA |
| 9 | 0.98 | 1.95 | 7.81 |
| 11 | 0.24 | 0.98 | 3.9 |
| 13 | 0.12 | 0.48 | 3.9 |
| 17a | 7.81 | 15.63 | 500 |
| 17b | 7.81 | 31.25 | NA |
| 17c | 0.24 | 0.98 | 7.81 |
| 17d | 3.9 | 7.81 | 62.5 |
| 17e | 0.98 | 1.95 | 3.9 |
| 17f | 0.98 | 3.9 | NA |
| 17g | 3.9 | 15.63 | 125 |
| 18a | 1.95 | 7.81 | 31.25 |
| 18b | 0.49 | 0.98 | 7.81 |
| 18c | 0.49 | 1.95 | 15.63 |
| 18d | 7.81 | 15.63 | NA |
| 18e | 3.9 | 15.63 | 250 |
| Vancomycin | 0.49 | 1.95 | ND |

**Table S2.** Minimum inhibitory concentrations (MICs, µg/mL) of the synthesized coumarin derivatives against Staphylococcus aureus strains (MSSA, MRSA, VRSA).

| **Sample** | ***Klebsiella pneumoniae*** | ***Pseudomonas aeruginosa*** | ***Acinetobacter baumannii*** | ***Salmonella typhimurium*** | ***Escherichia coli*** |
| --- | --- | --- | --- | --- | --- |
| 5 | 21 | 14 | 8 | 22 | 14 |
| 7 | 12 | 18 | 12 | 18 | 9 |
| 9 | NA | 13 | NA | 11 | NA |
| 11 | 18 | 24 | 19 | 20 | 11 |
| 13 | 13 | 25 | 14 | 9 | 33 |
| 17a | 23 | 9 | NA | 17 | 17 |
| 17b | 18 | 19 | 11 | 7 | 7 |
| 17c | 9 | 11 | 30 | 14 | 25 |
| 17d | 7 | NA | 32 | 12 | 26 |
| 17e | 17 | NA | 17 | 21 | 13 |
| 17f | 20 | 15 | 22 | 26 | 8 |
| 17g | NA | 29 | NA | NA | NA |
| 18a | 23 | NA | 21 | NA | 11 |
| 18b | NA | 22 | 20 | 22 | NA |
| 18c | 8 | 34 | 14 | 14 | 22 |
| 18d | NA | NA | 33 | NA | NA |
| 18e | 14 | 13 | NA | 18 | 19 |
| Gentamycin | 17 | 20 | 24 | 26 | 30 |
| DMSO | 0 | 0 | 0 | 0 | 0 |

**Table S3.** Antibacterial activity against Gram-negative pathogens, expressed as inhibition zone diameters (mm) at 10 µg/mL.

| **Sample Code** | **IC_50_ values (µg/mL)** |
| --- | --- |
| 5 | 365.1 ± 2.3 |
| 7 | 270.2 ± 4.8 |
| 9 | 341.8 ± 5.1 |
| 11 | 255.3 ± 1.8 |
| 13 | 220.7 ± 4.0 |
| 17a | 312.9± 1.2 |
| 17b | 285.2 ± 5.3 |
| 17c | 291.7 ± 2.4 |
| 17d | 308 ± 3.1 |
| 17e | 406.3 ± 1.9 |
| 17f | 225.8 ± 2.7 |
| 17g | 219.3 ± 4.1 |
| 18a | 266.4 ± 3.6 |
| 18b | 238.5 ± 2.2 |
| 18c | 442.0± 4.0 |
| 18d | 294.4 ± 3.4 |
| 18e | 320.4 ± 1.5 |

**Table S4.** IC₅₀ values (µg/mL) of all synthesized coumarin derivatives against WI-38 normal human lung fibroblast cells.

| **Ligand** | **Homo sapiens** | | **Acinetobacter baumannii** | **Escherichia coli** | | **Pseudomonas aeruginosa** | **Staphylococcus aureus** | |
| --- | --- | --- | --- | --- | --- | --- | --- | --- |
|  | **TP-** **IIα** | | **DG-B** | **DG-B** | **TP-IV** | **DG-B** | **DG-B** | **TP-IV** |
|  | **1ZXM** | | **7PQL** | **6F94** | **1S14** | **7PTG** | **6TCK** | **4URN** |
|  | **Mg bound** | **Mg non-bound** |  |  |  |  |  |  |
| 11 | -10.6 | -11.4 | -10.3 | -10.5 | -11.5 | -9.7 | -10.9 | -11.5 |
| 13 | -10.9 | -12.3 | -10.6 | -10.4 | -10.7 | -10.2 | -11.6 | -13.8 |
| 17c | -14.1 | -11.9 | -11.9 | -11.2 | -12.4 | -12.1 | -12.8 | -11.8 |
| 17e | -12.5 | -11.6 | -10.9 | -10.2 | -12.3 | -11.5 | -12.1 | -10.0 |
| 18b | -9.8 | -11.9 | -10.9 | -11.6 | -11.4 | -12.1 | -11.1 | -11.1 |
| Ref. | -18.4 | NA | -11.6 | -10.4 | -10.6 | -11.2 | -10.7 | -10.8 |

**Table S5A.** Docking scores for all synthesized compounds across selected topoisomerases.

| **Ligand** | **Homo sapiens** | | **Acinetobacter baumannii** | **Escherichia coli** | | **Pseudomonas aeruginosa** | **Staphylococcus aureus** | |
| --- | --- | --- | --- | --- | --- | --- | --- | --- |
|  | **TP-** **IIα** | | **DG-B** | **DG-B** | **TP-IV** | **DG-B** | **DG-B** | **TP-IV** |
|  | **1ZXM** | | **7PQL** | **6F94** | **1S14** | **7PTG** | **6TCK** | **4URN** |
|  | **Mg bound** | **Mg non-bound** |  |  |  |  |  |  |
| 11 | -49.4 | -53.1 | -35.7 | -30.6 | -45.6 | -41.6 | -31.5 | -45.0 |
| 13 | -50.9 | -51.0 | -35.2 | -20.0 | -40.8 | -40.1 | -47.3 | -40.4 |
| 17c | -46.2 | -52.7 | -41.7 | -40.8 | -47.1 | -43.1 | -42.3 | -46.2 |
| 17e | -44.3 | -55.0 | -38.4 | -43.5 | -45.4 | -42.8 | -44.3 | -46.2 |
| 18b | -53.3 | -56.5 | -40.7 | -43.1 | -36.9 | -49.7 | -41.3 | -50.5 |
| Ref. | -84.5 | NA | -48.0 | -52.7 | -39.0 | -54.3 | -43.8 | -48.2 |

**Table S5B.** MM/GBSA binding free energies (kcal/mol) for all synthesized compounds across selected topoisomerases.

**Supplementary Video**

**Video S1.** Molecular dynamics (MD) trajectory showing the hydrogen bond formation between compound 13 and SER129 in S. aureus DNA gyrase (6TCK). *Provided as a separate video file.*

**Supplementary Reports**

**Report S1.** ProTox-3.0 full toxicity report for compound 13, detailing predicted toxicological endpoints and risk assessments. *Provided as a separate PDF file.*

**Report S2.** Dose-response curves from the MTT cytotoxicity assay against HeLa cervical cancer cells, including IC₅₀ values for the tested compounds. *Provided as a separate PDF file.*

**Spectra**


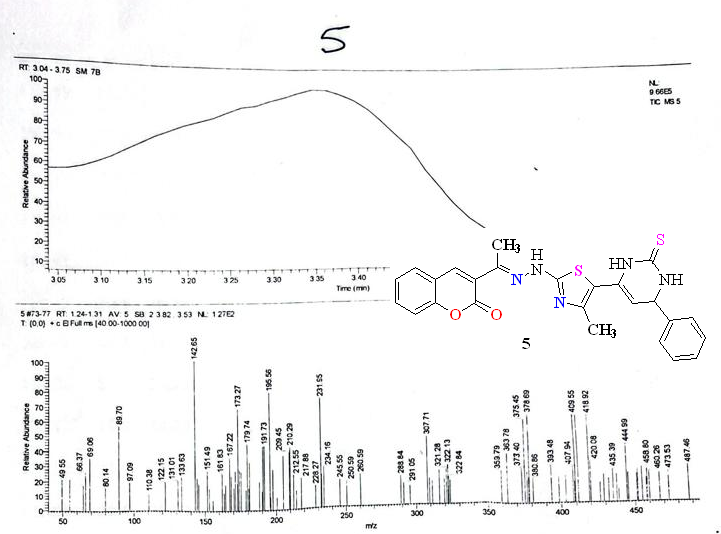


**Mass spectrum compound 5**


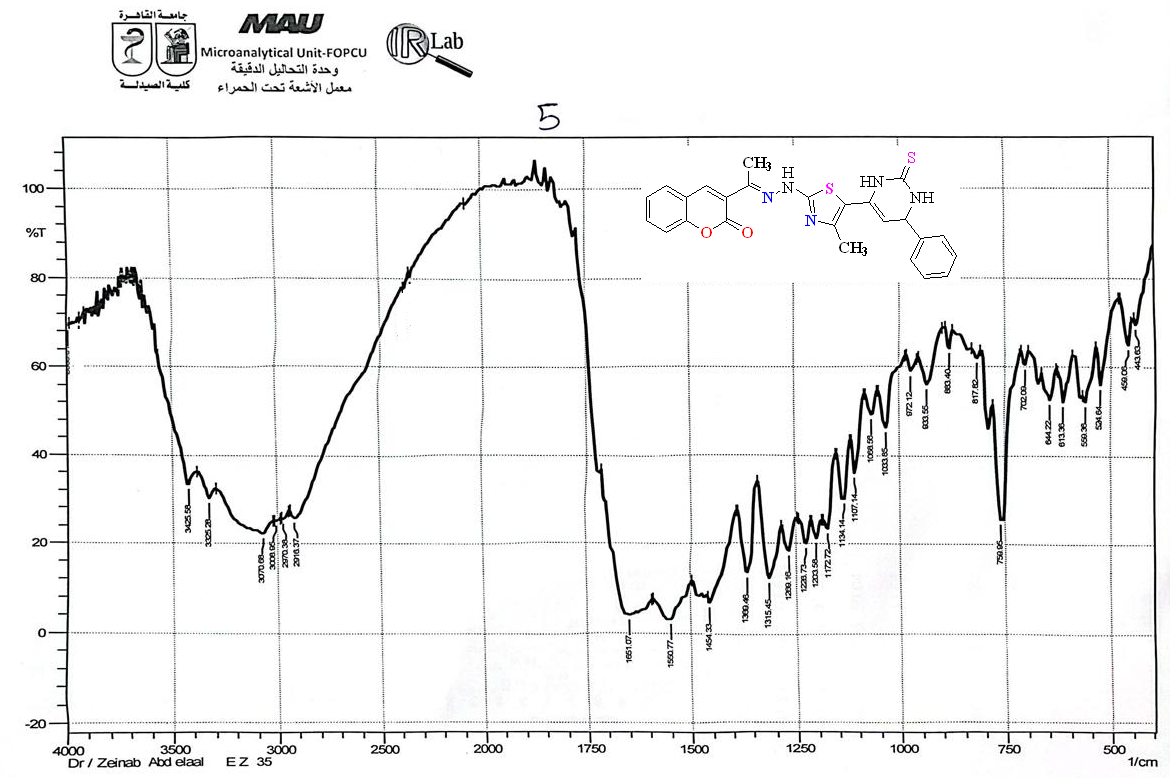


**IR spectrum compound 5**


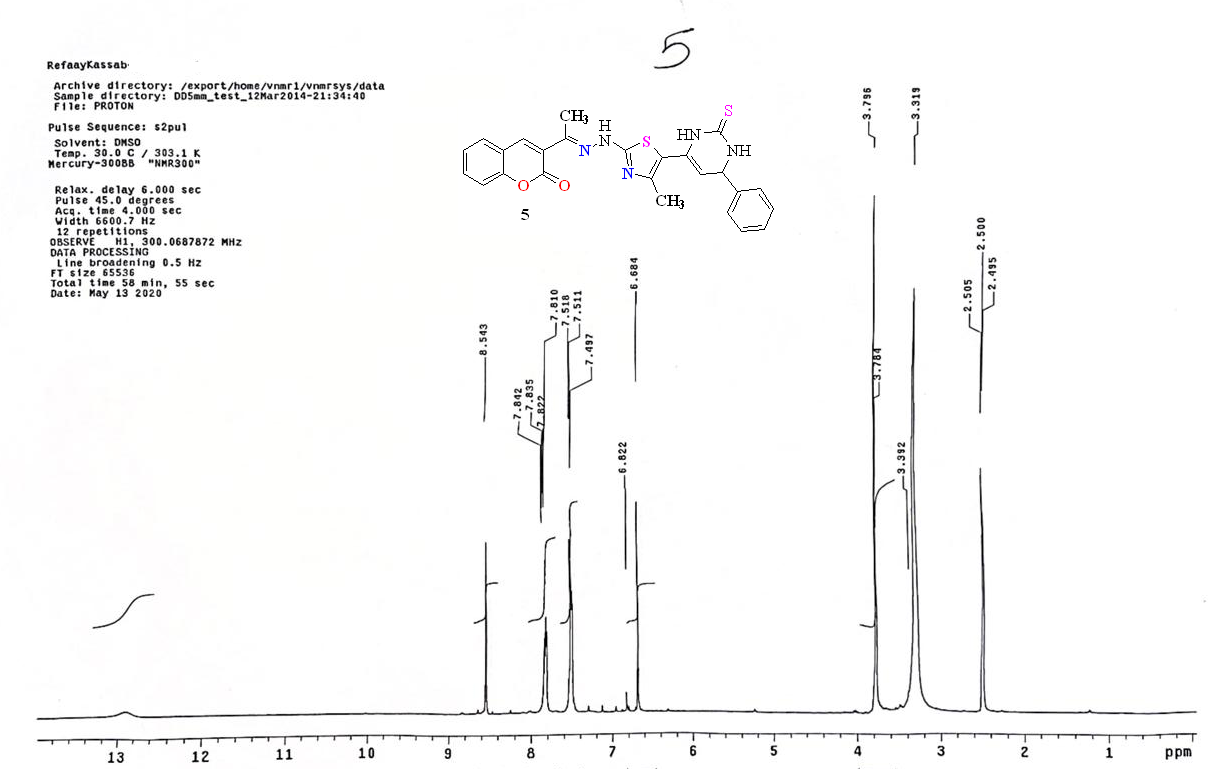


**^I^H NMR spectrum compound 5**


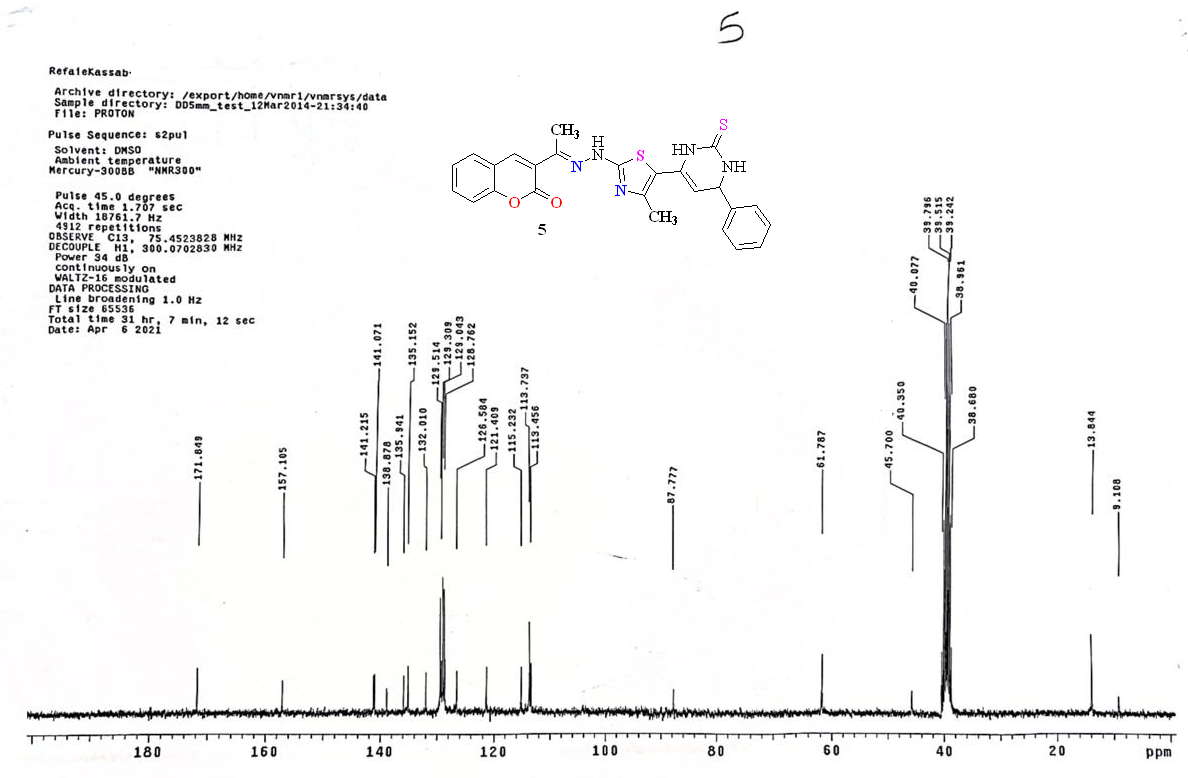


**^13^C NMR spectrum compound 5**


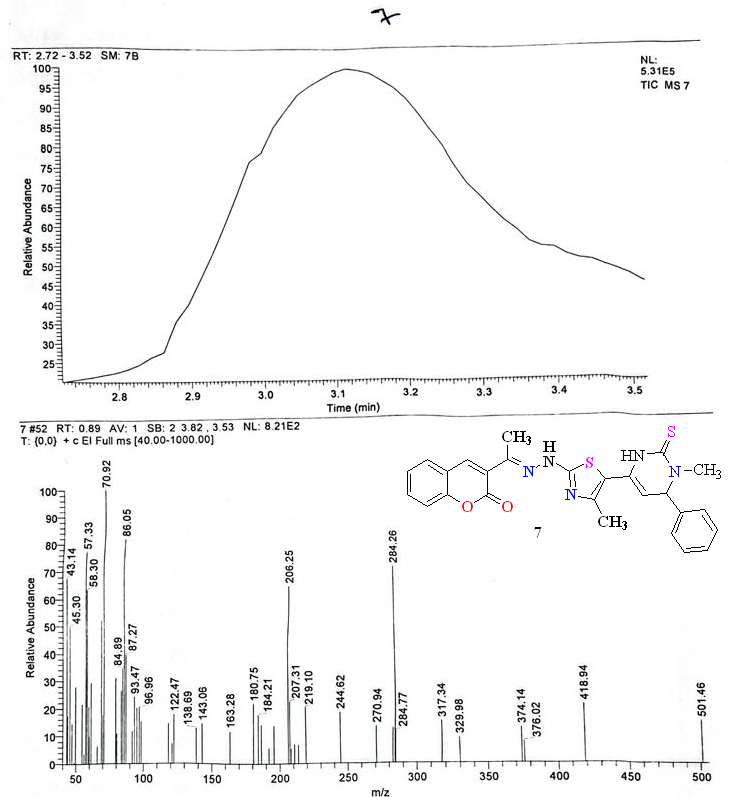


**Mass spectrum compound 7**


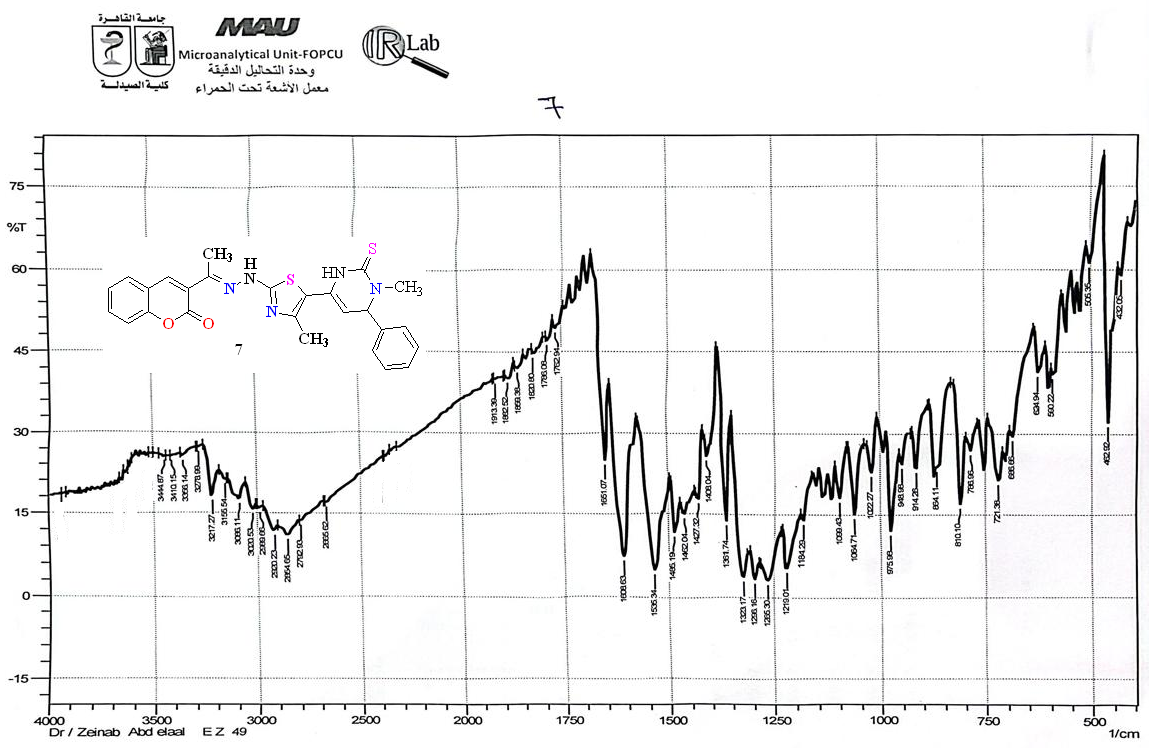


**IR spectrum compound 7**


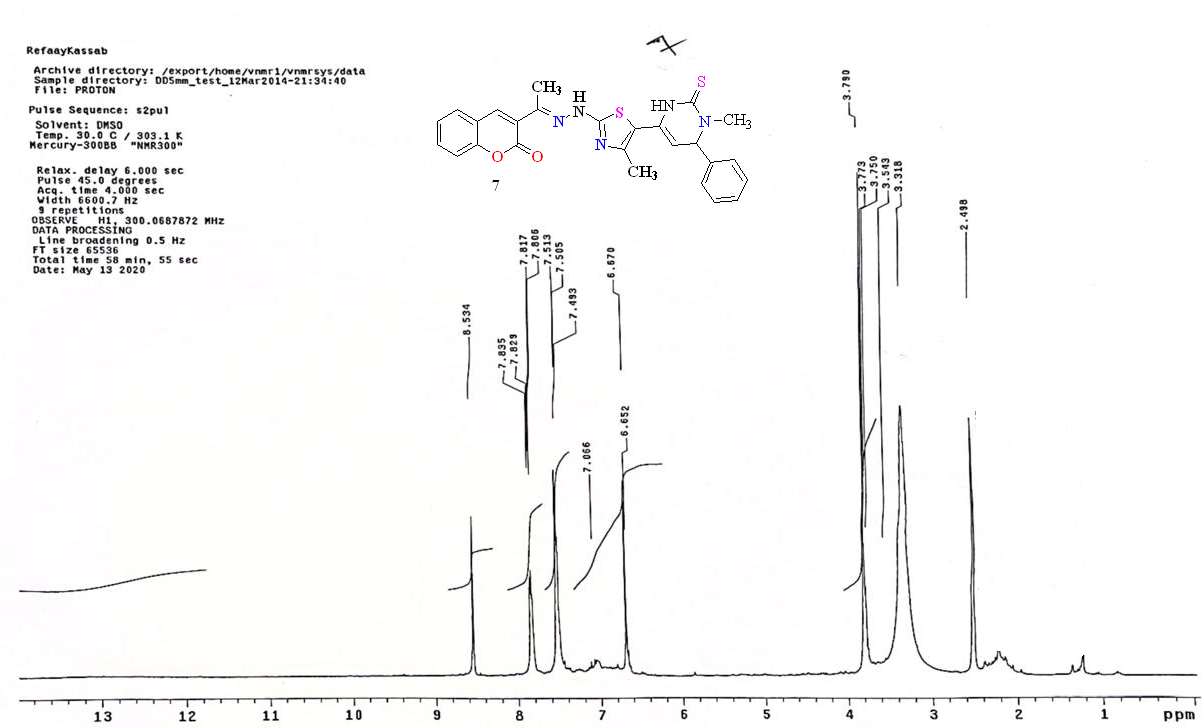


**^I^H NMR spectrum compound 7**


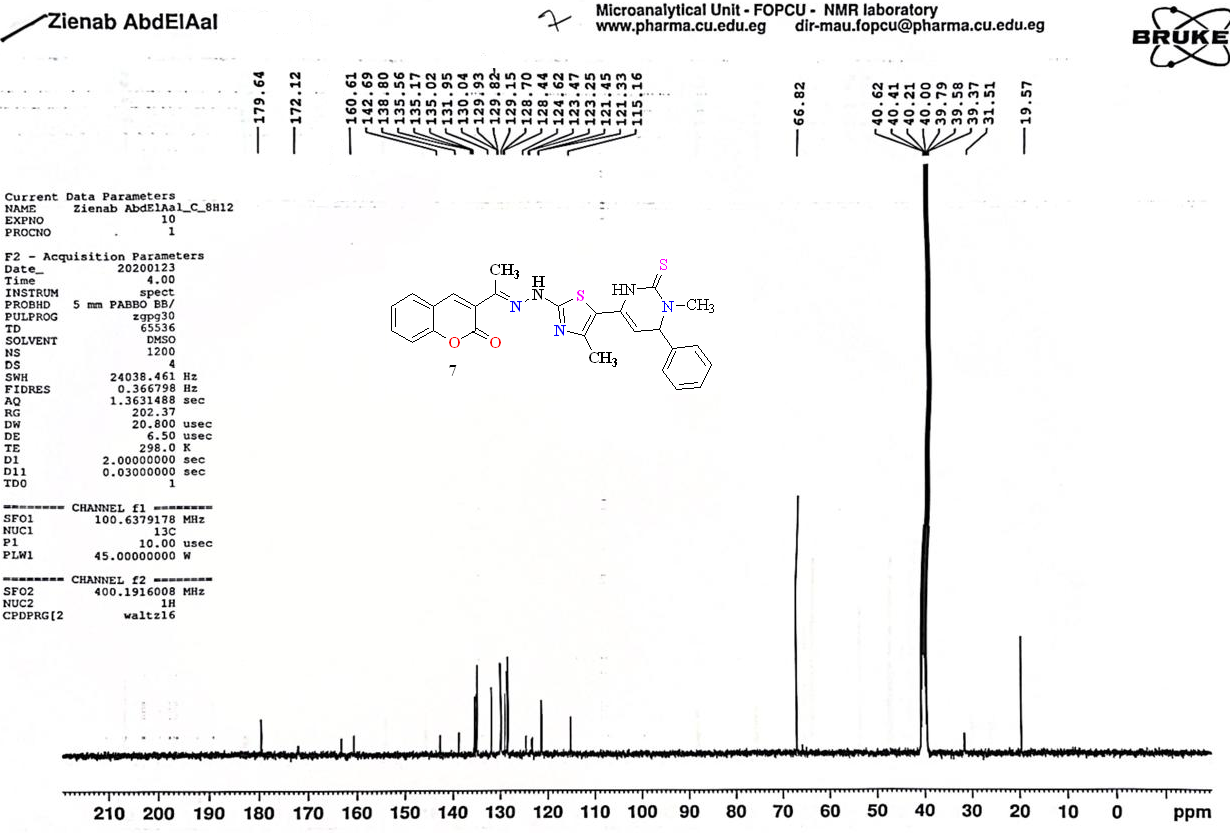


**^13^C NMR spectrum compound 7**


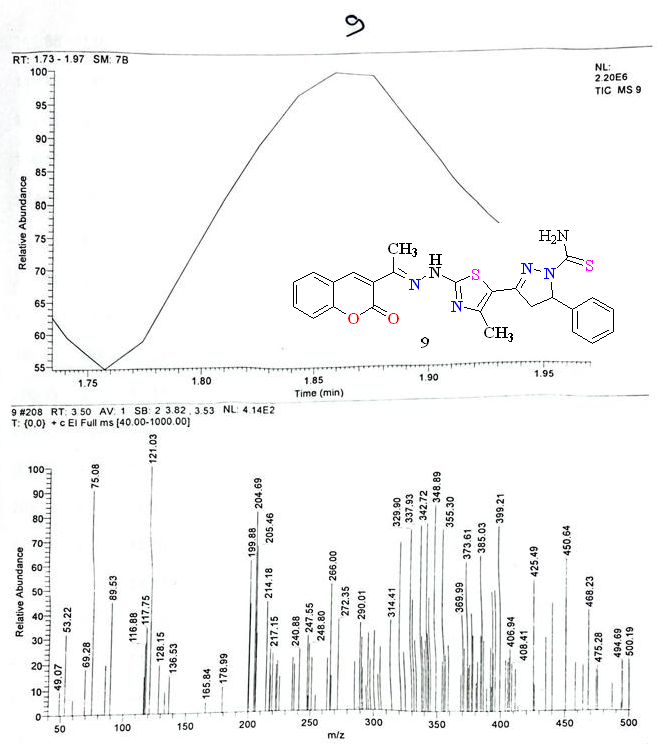


**Mass spectrum compound 9**


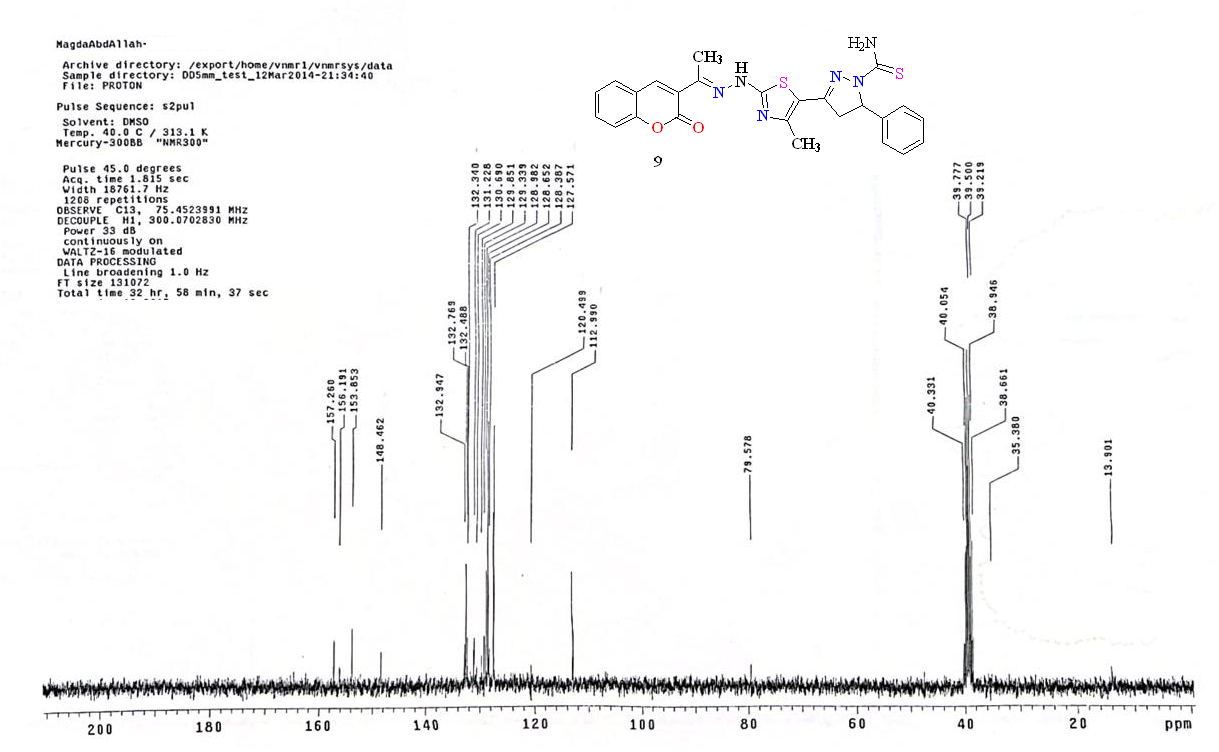


**^13^C NMR spectrum compound 9**


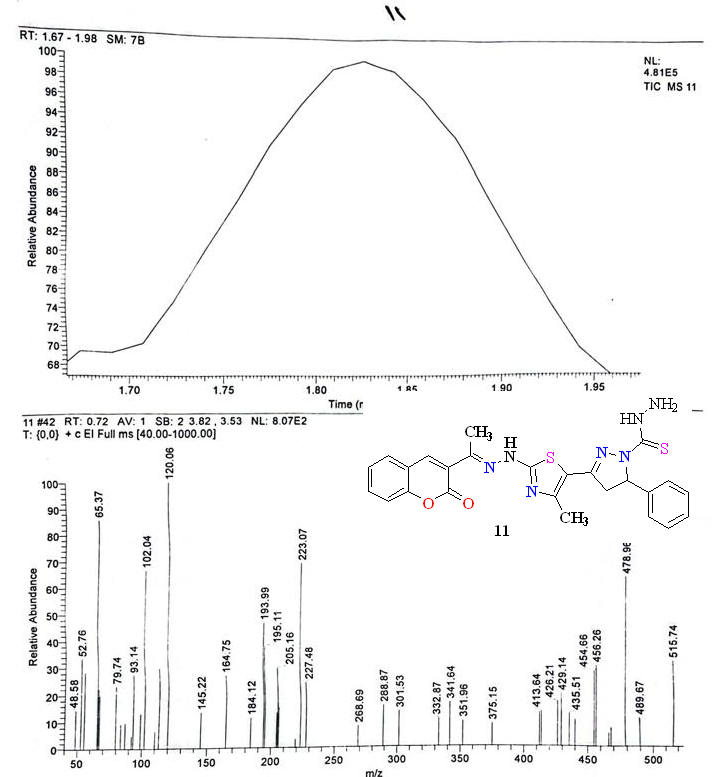


**Mass spectrum compound 11**


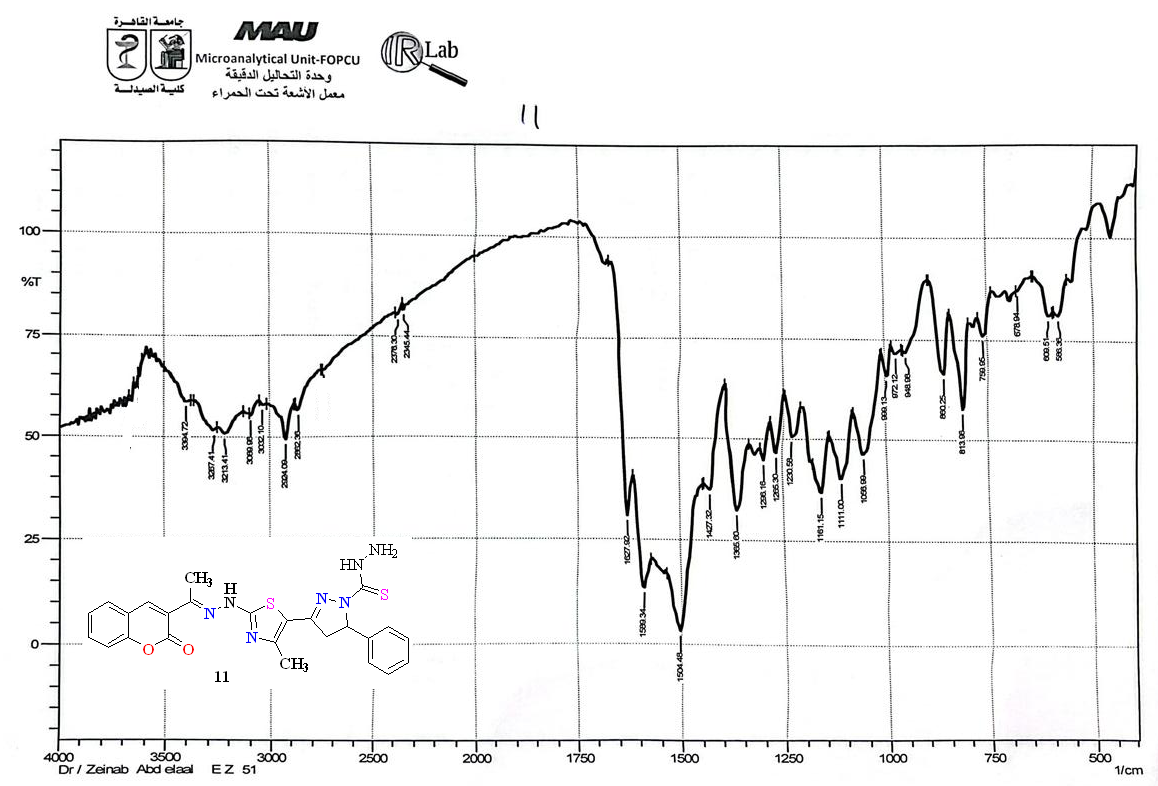


**IR spectrum compound 11**


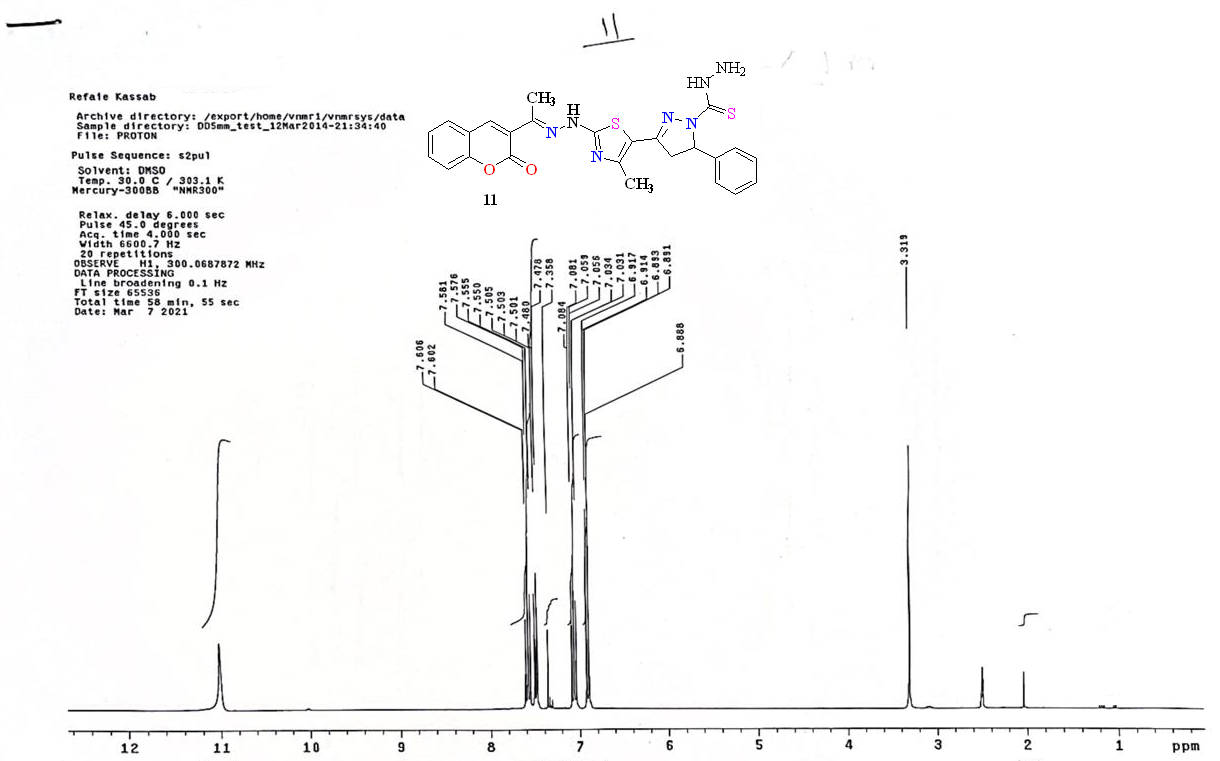


**^I^H NMR spectrum compound 11**


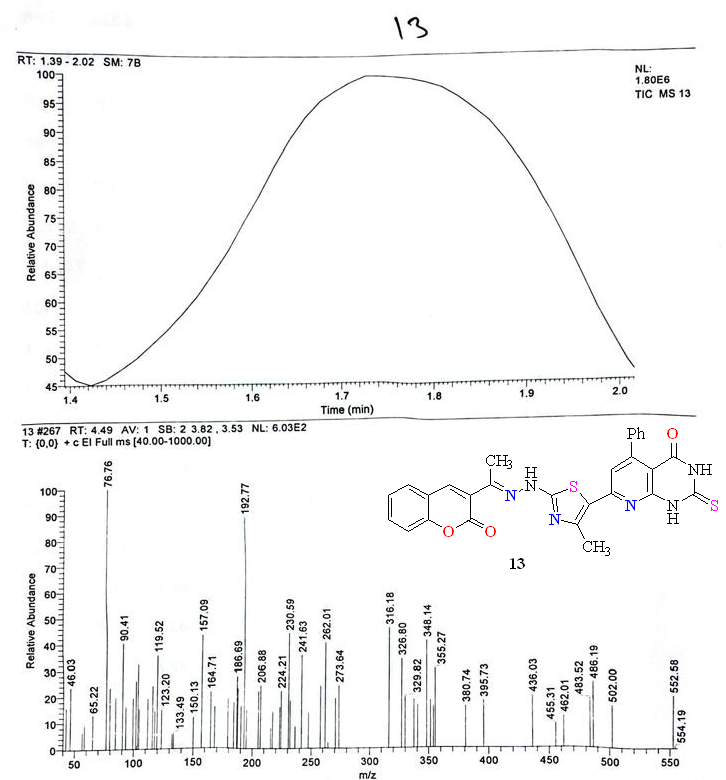


**Mass spectrum compound 13**


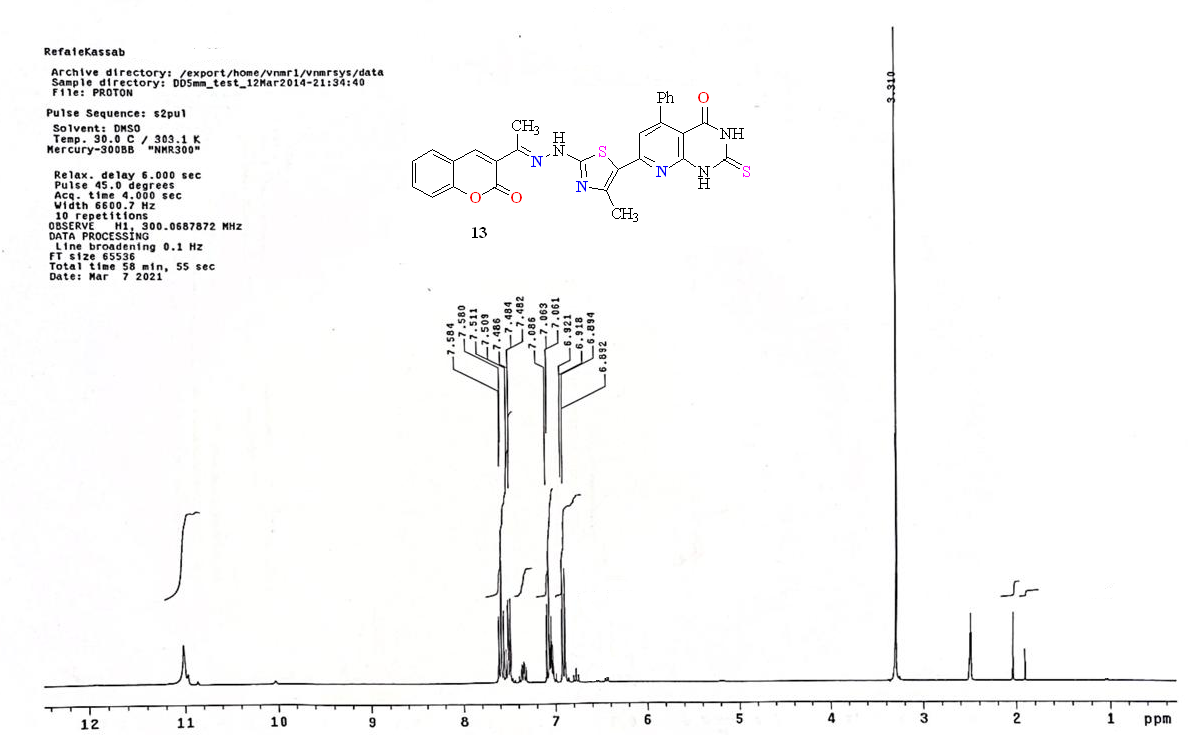


**^I^H NMR spectrum compound 13**


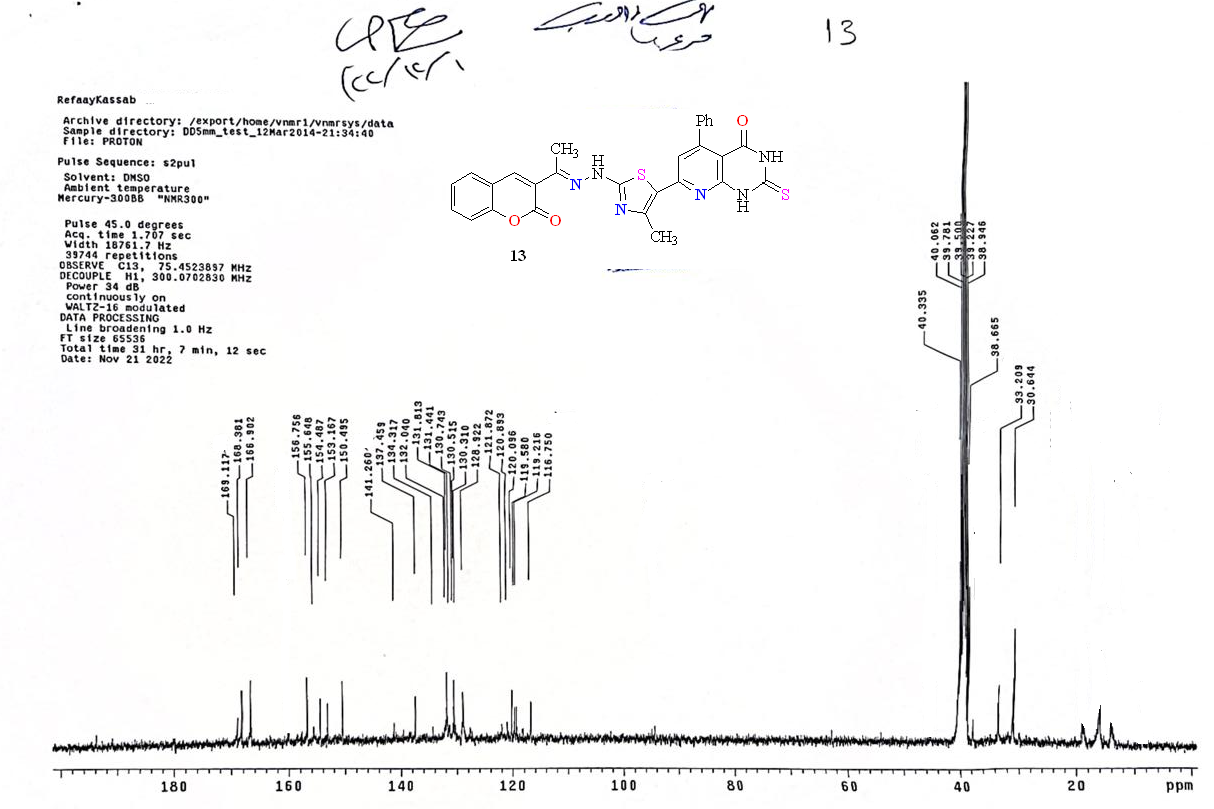


**^13^C NMR spectrum compound 13**


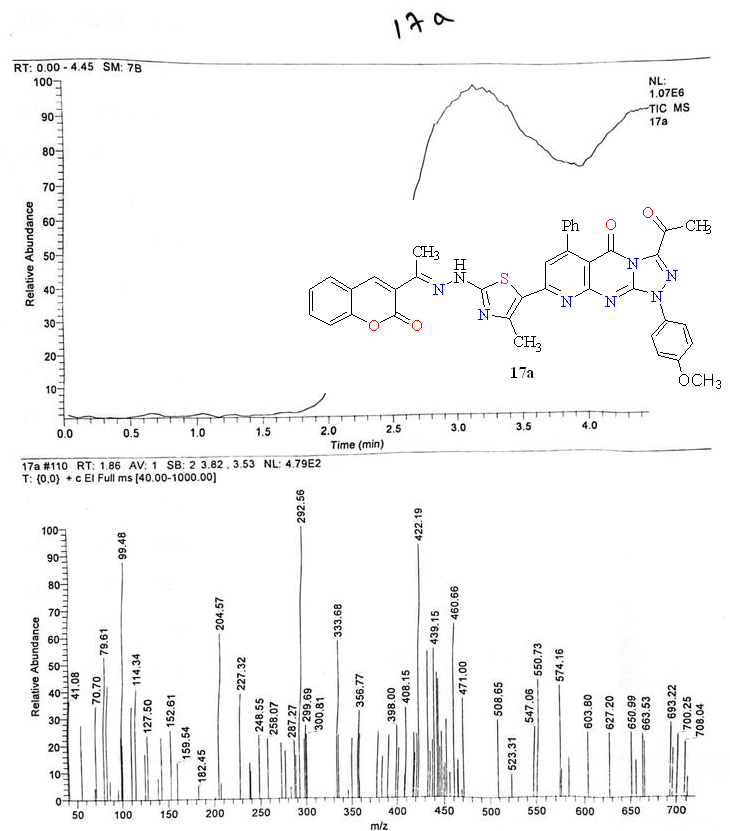


**Mass spectrum compound 17a**


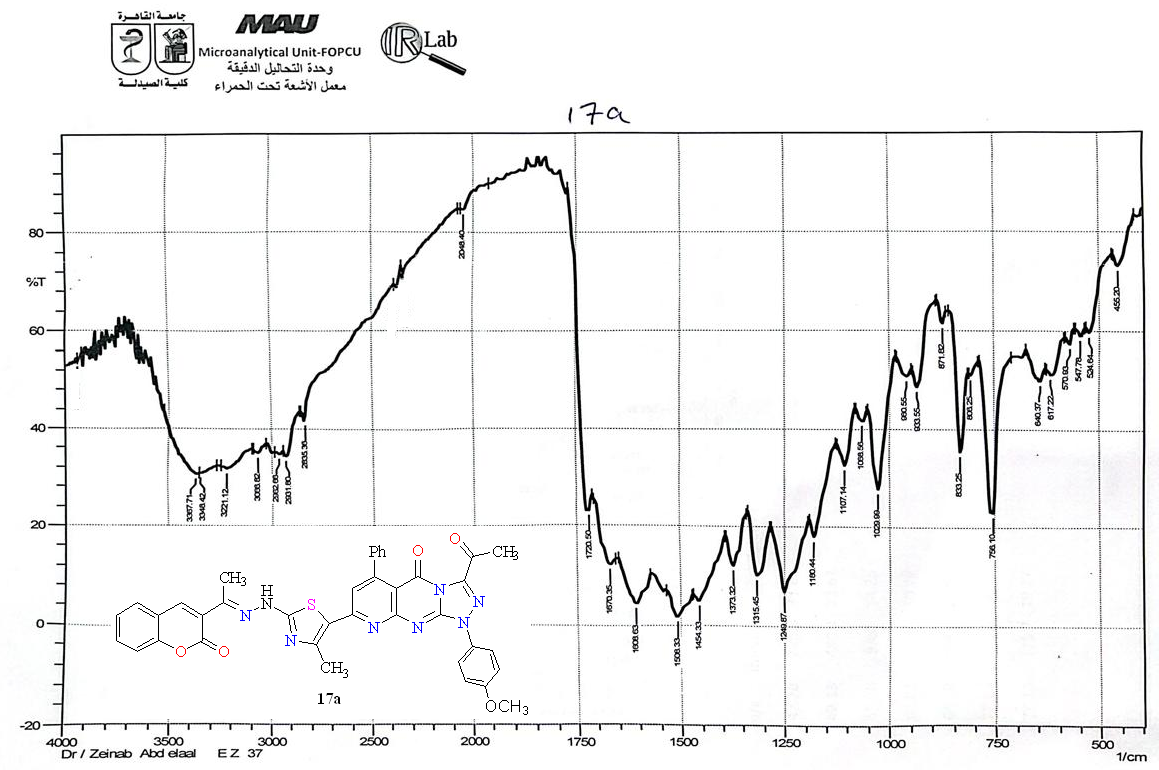


**IR spectrum compound 17a**


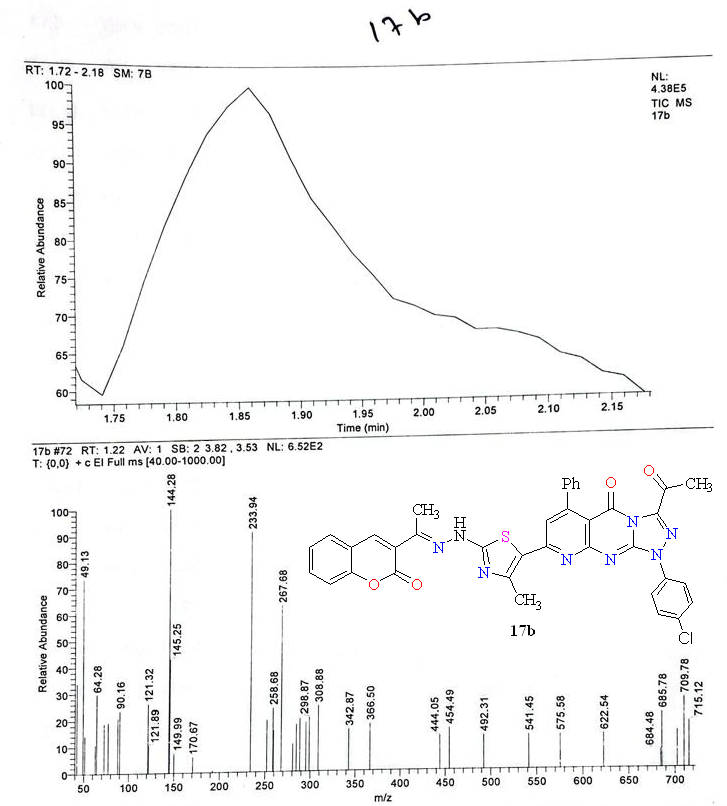


**Mass spectrum compound 17b**


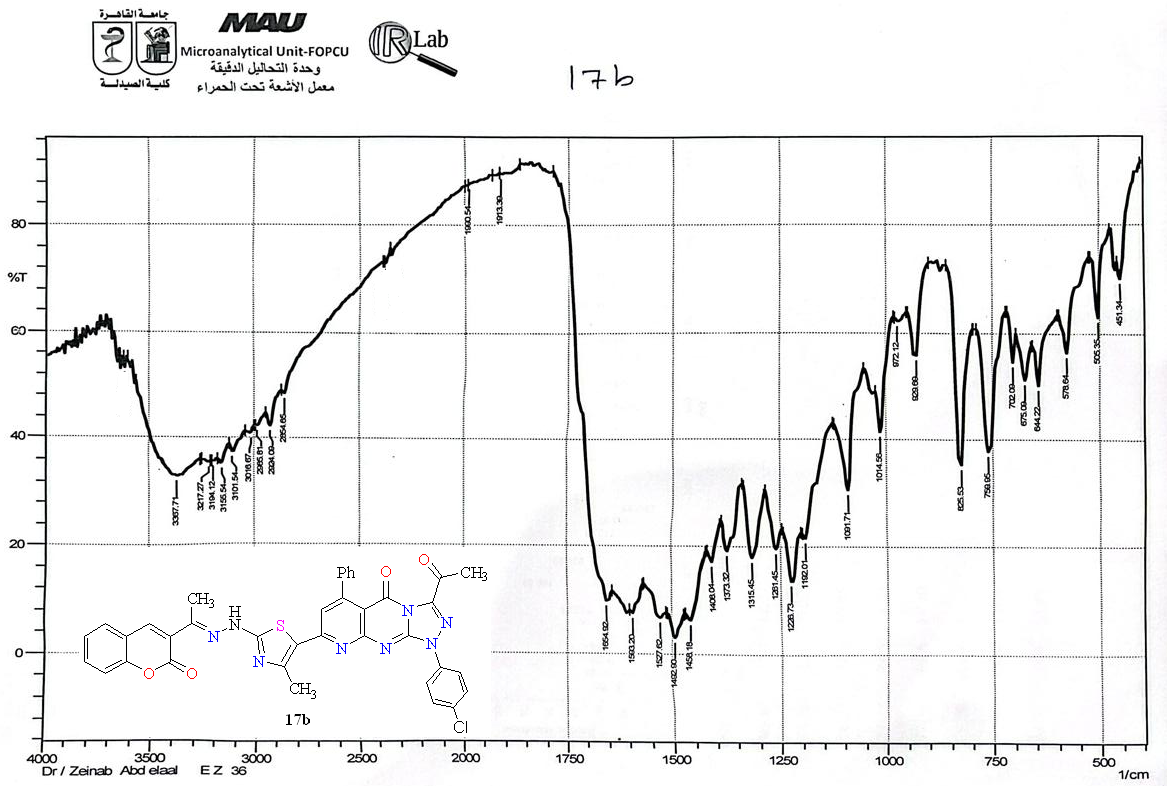


**IR spectrum compound 17b**


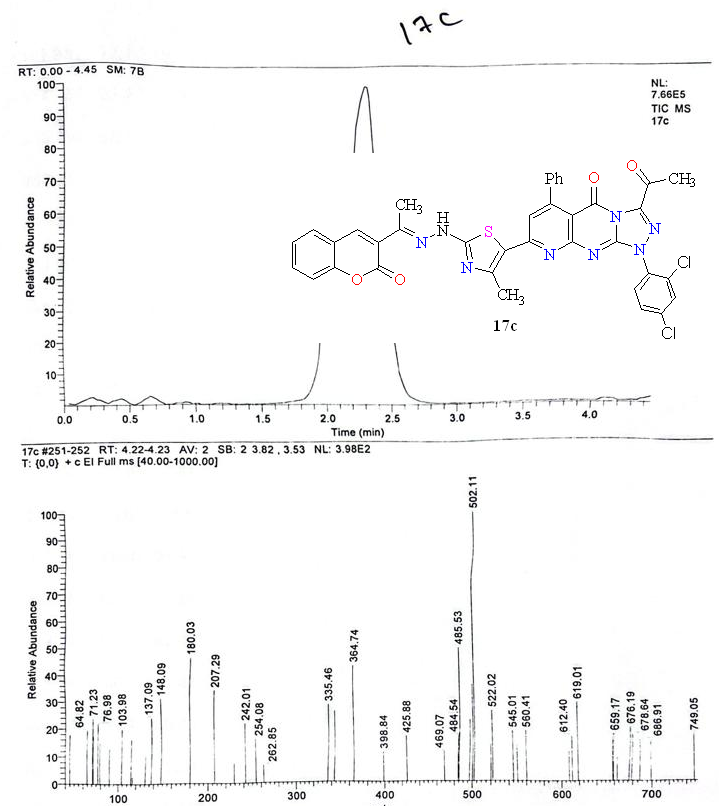


**Mass spectrum compound 17c**


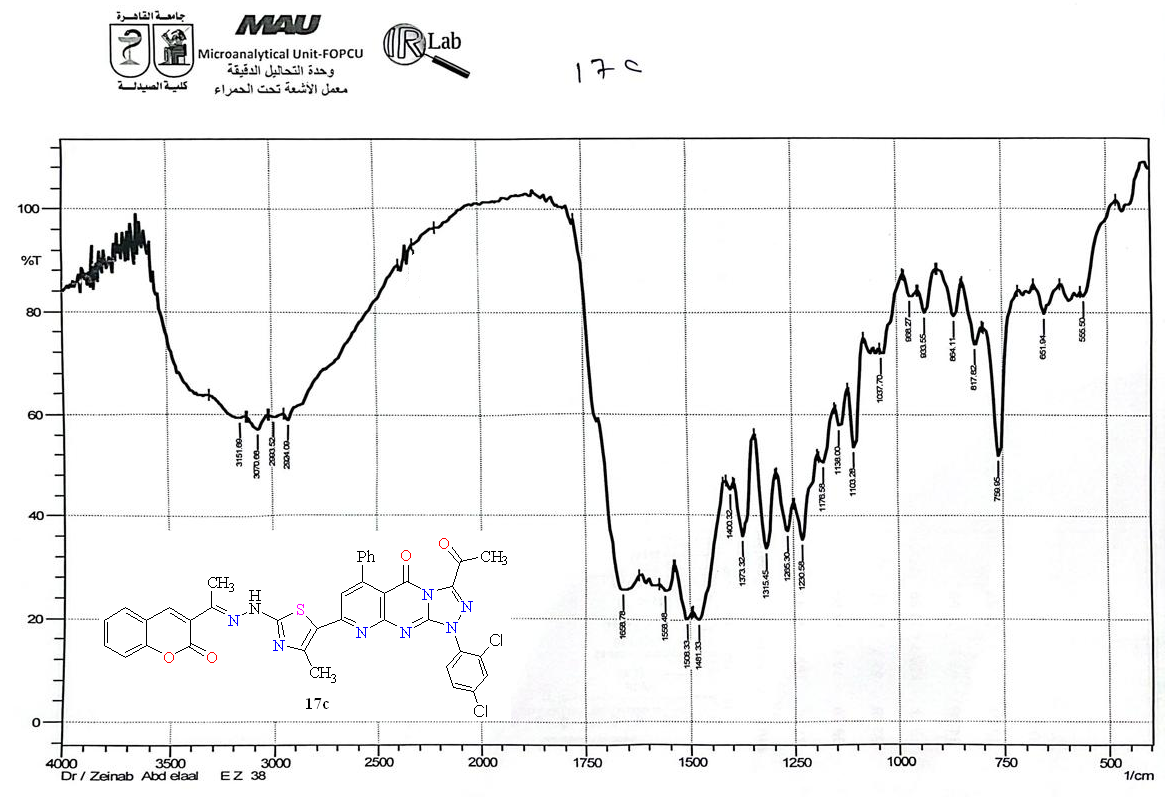


**IR spectrum compound 17c**


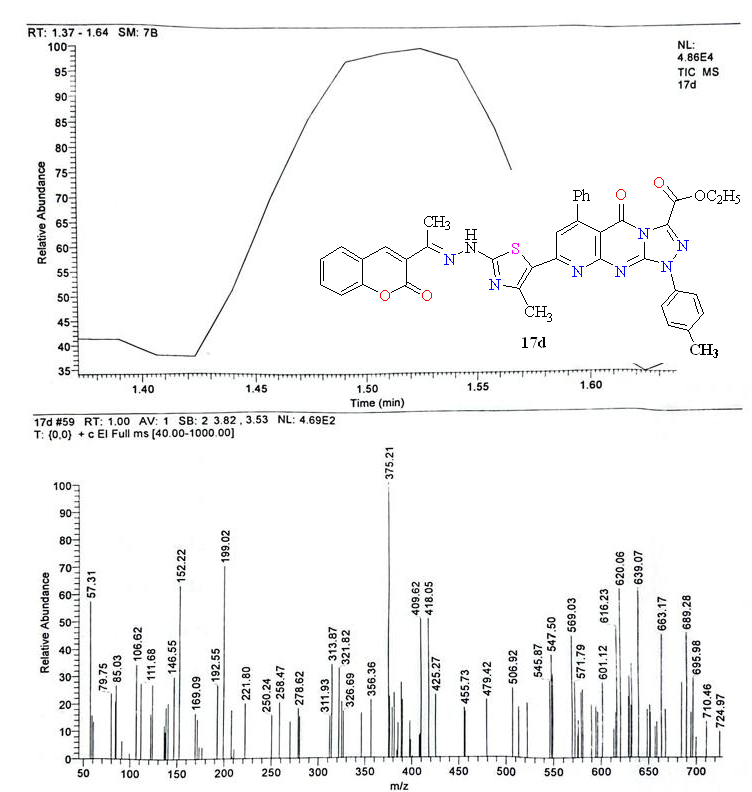


**Mass spectrum compound 17d**


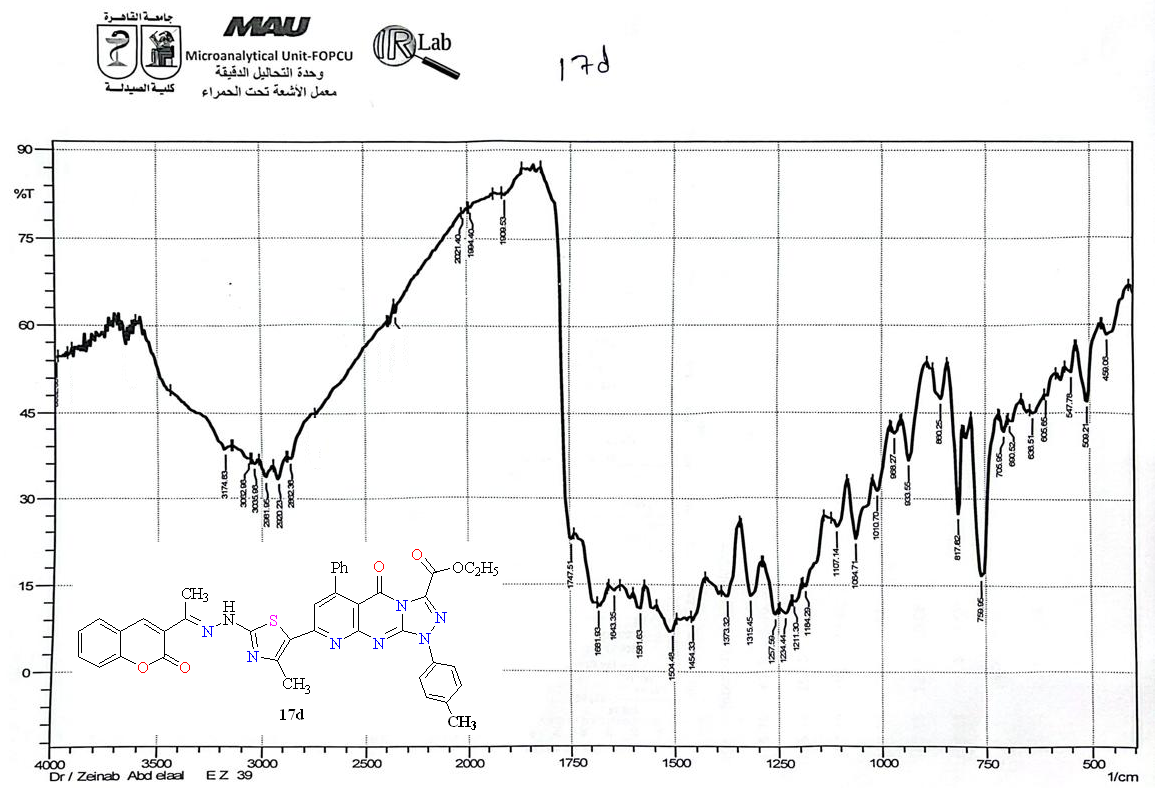


**IR spectrum compound 17d**


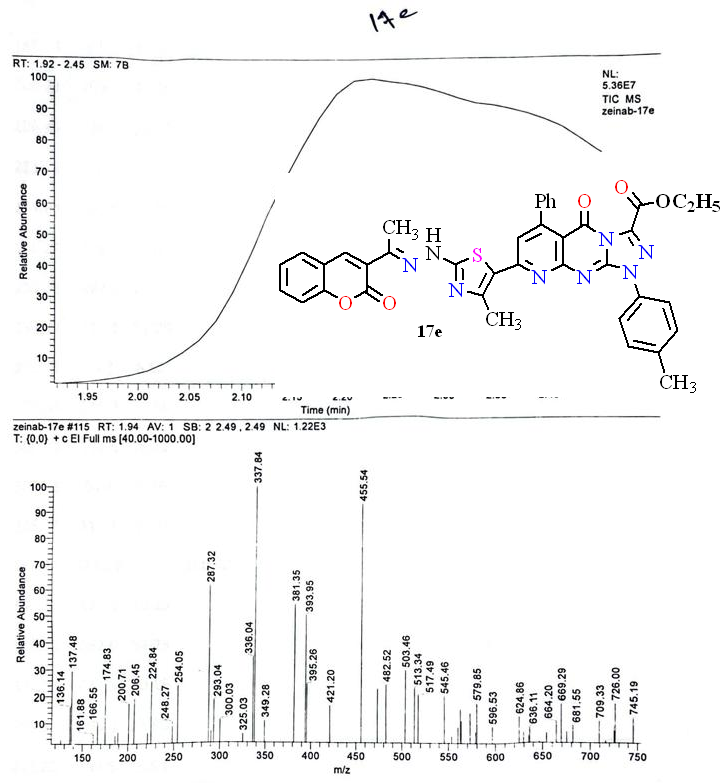


**Mass spectrum compound 17e**


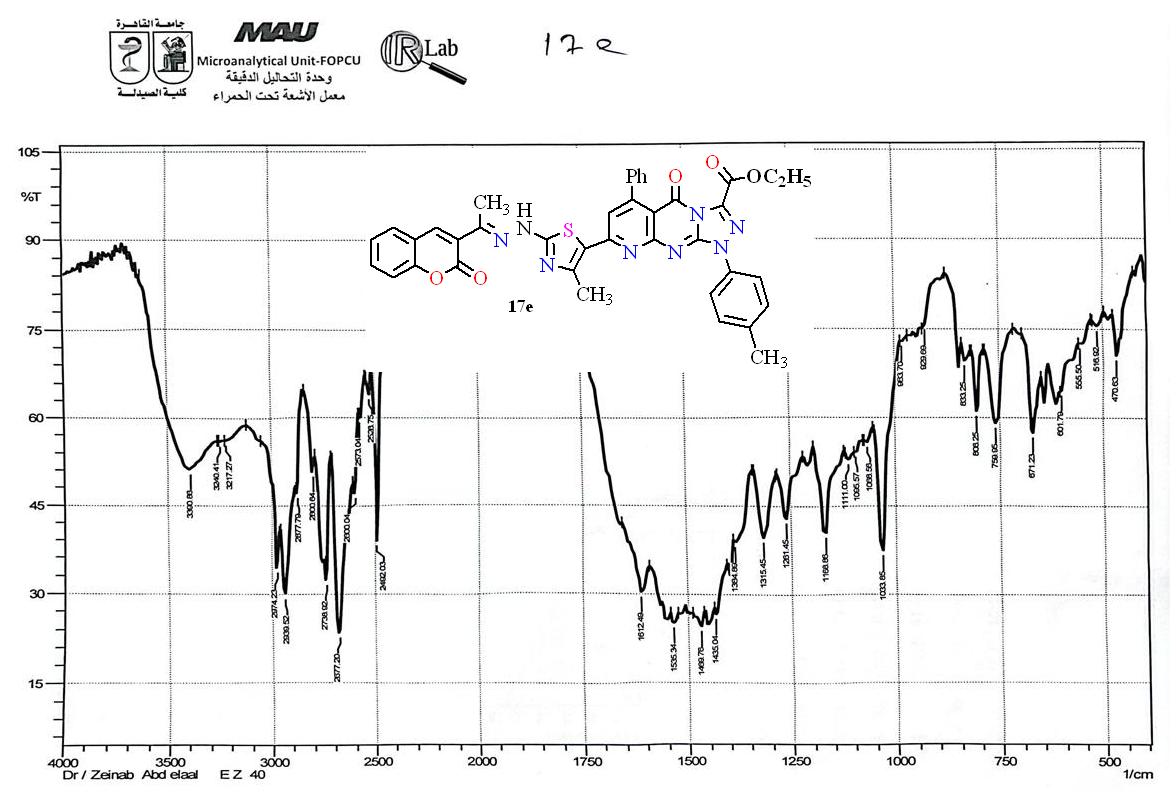


**IR spectrum compound 17e**


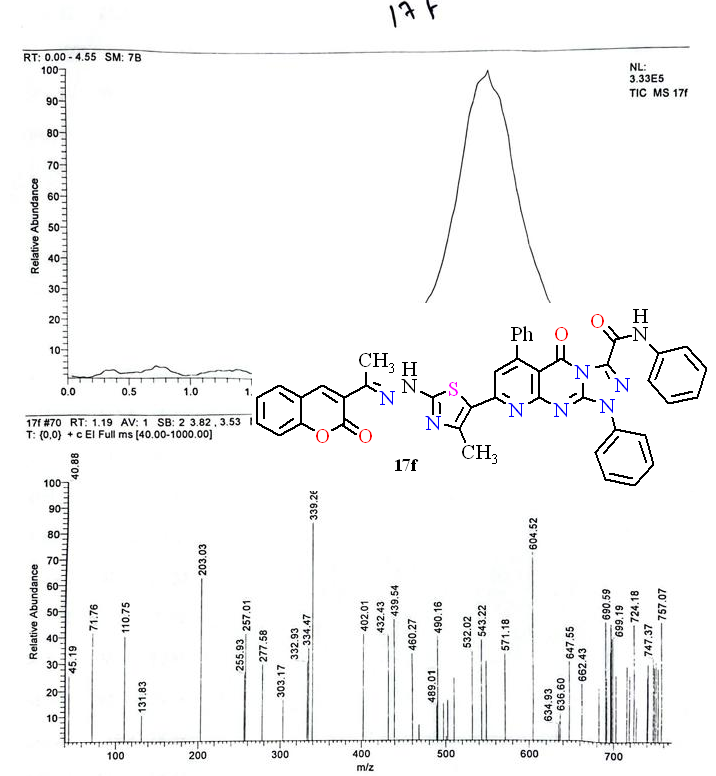


**Mass spectrum compound 17f**


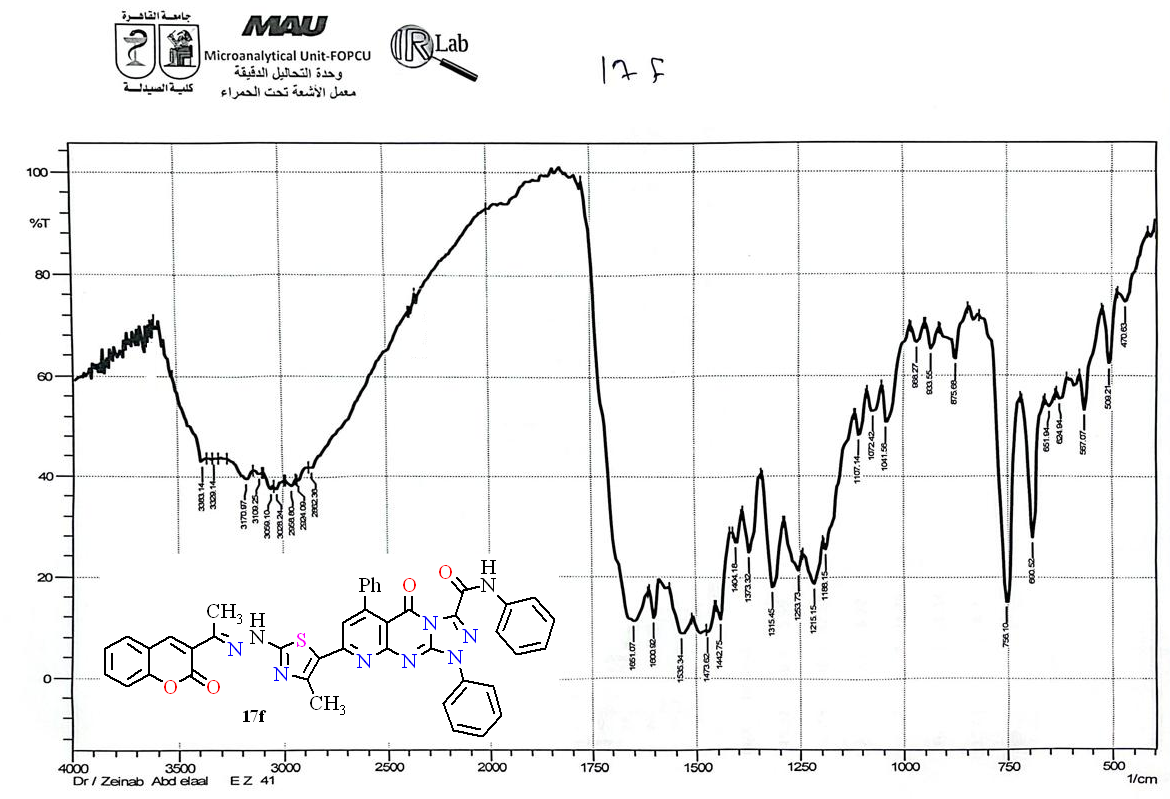


**IR spectrum compound 17f**


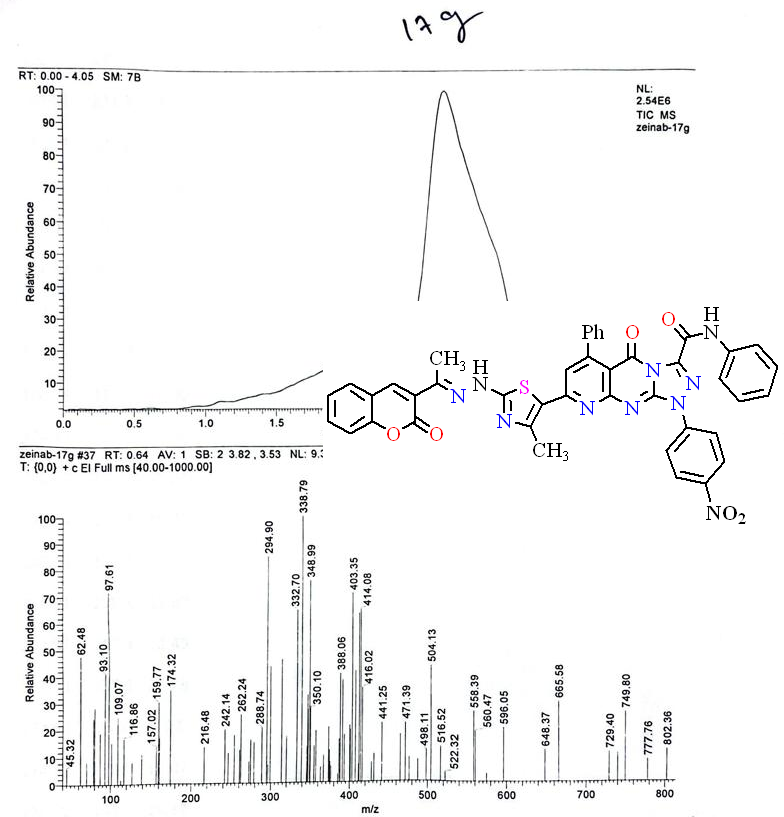


**Mass spectrum compound 17g**


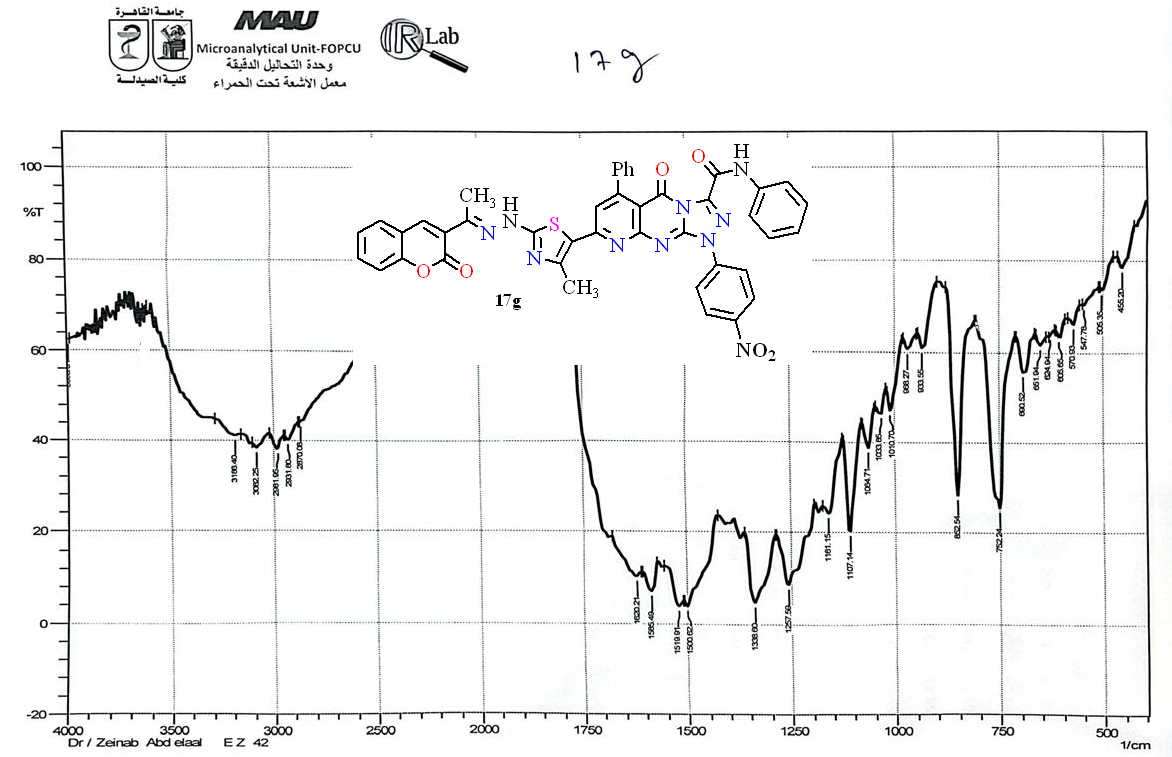


**IR spectrum compound 17g**


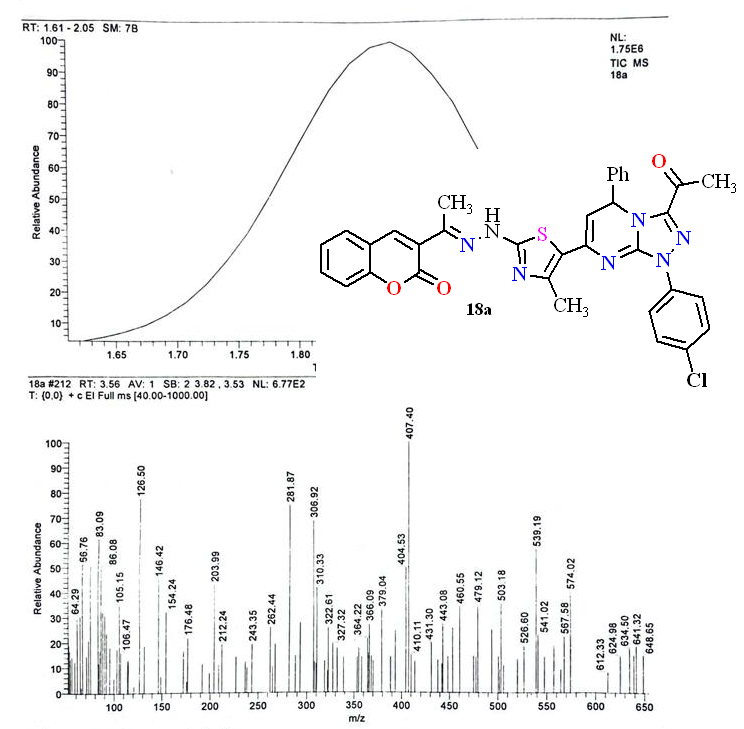


**Mass spectrum compound 18a**


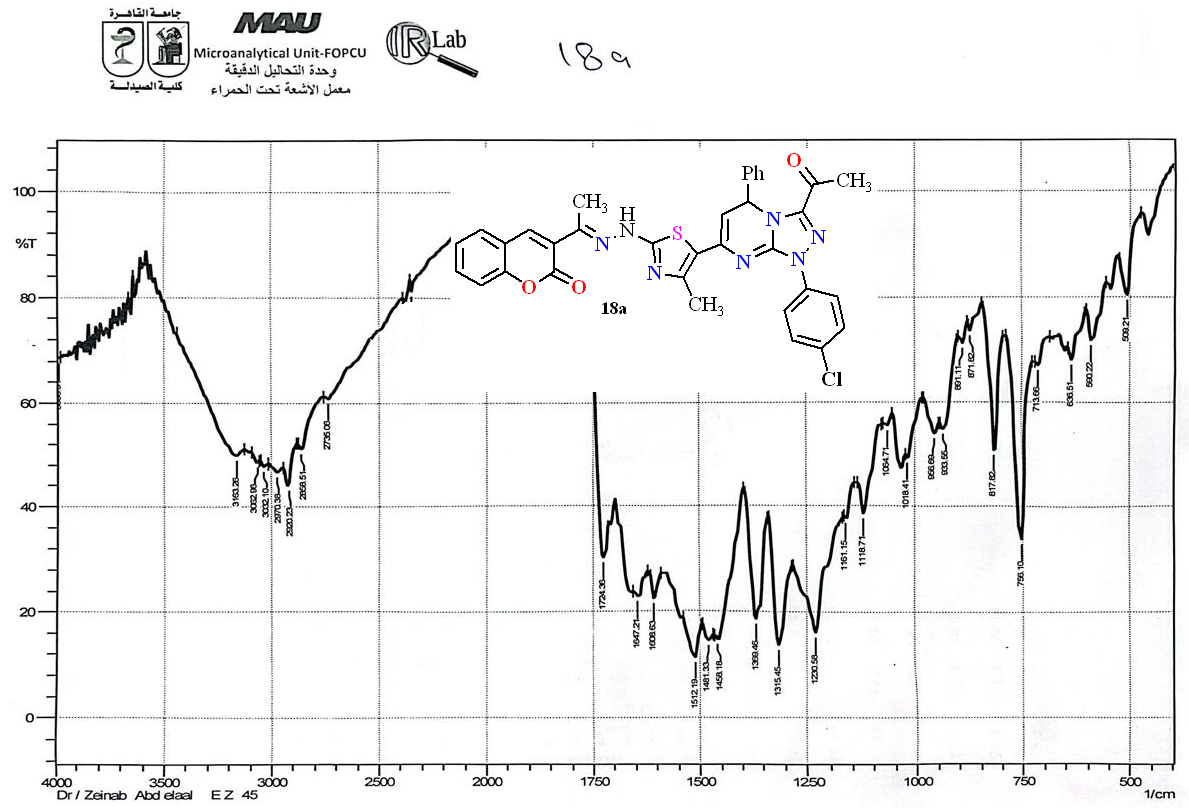


**IR spectrum compound 18a**


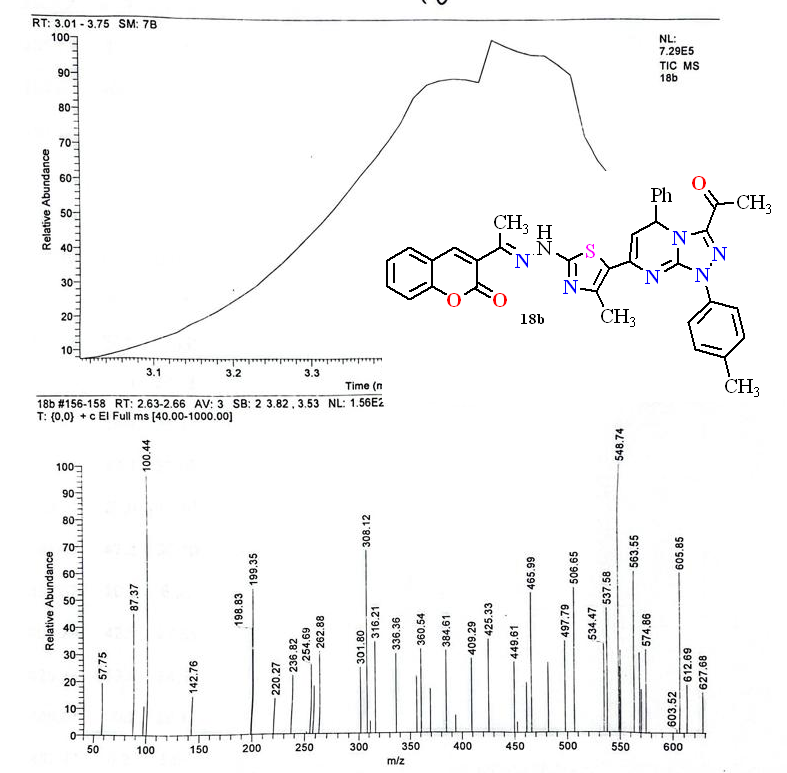


**Mass spectrum compound 18b**


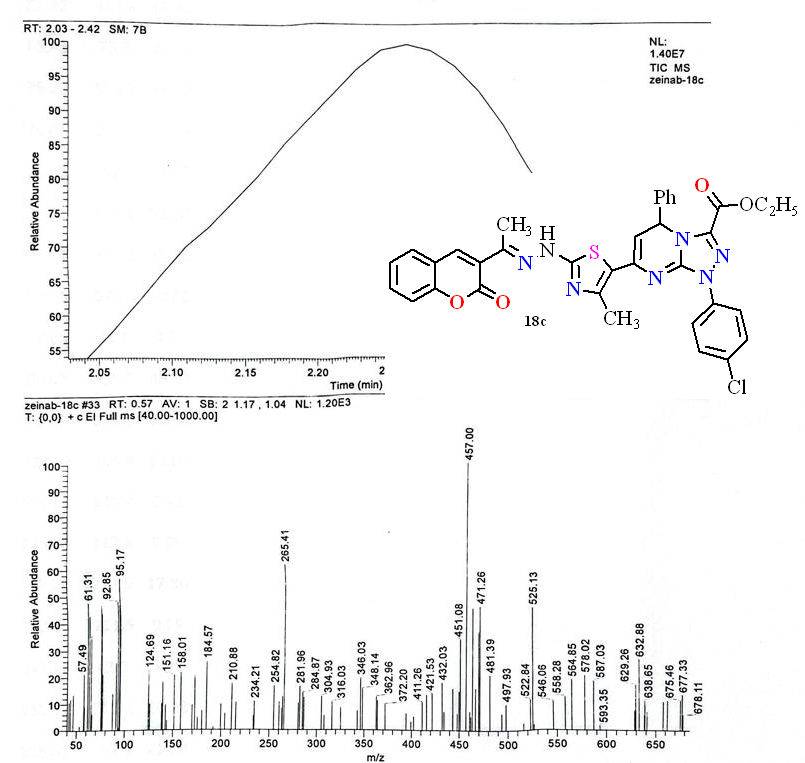


**Mass spectrum compound 18c**


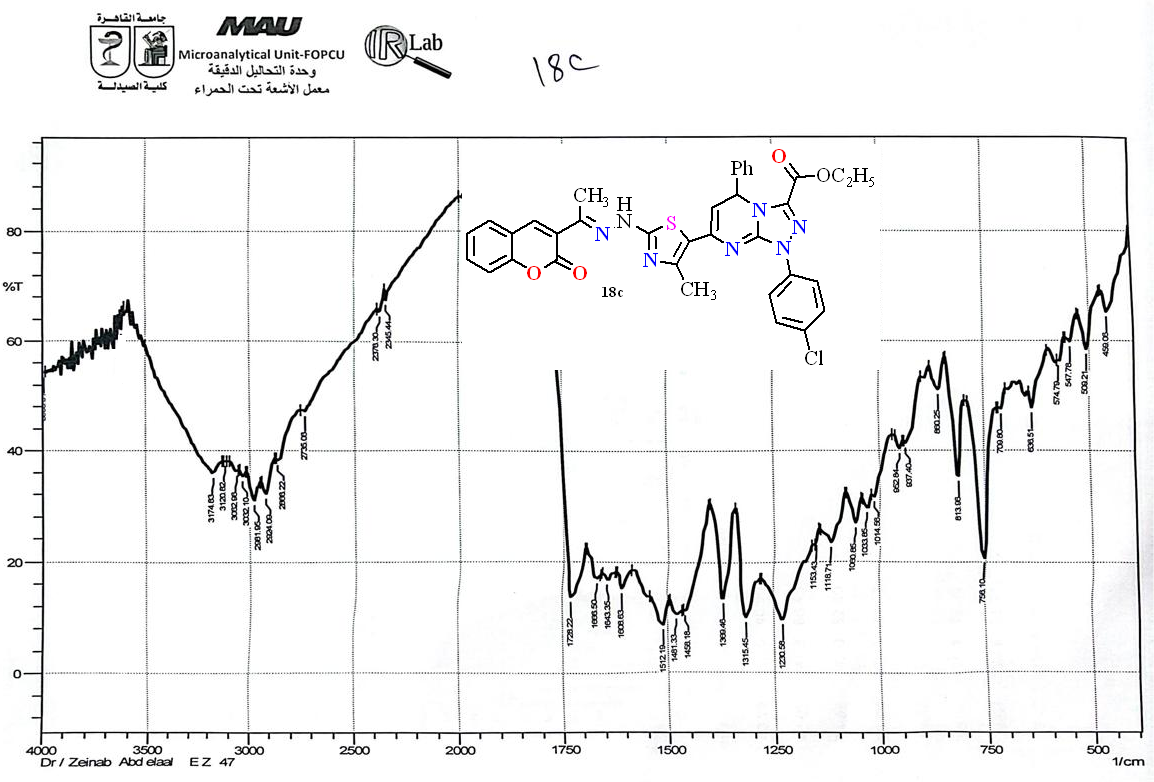


**IR spectrum compound 18c**


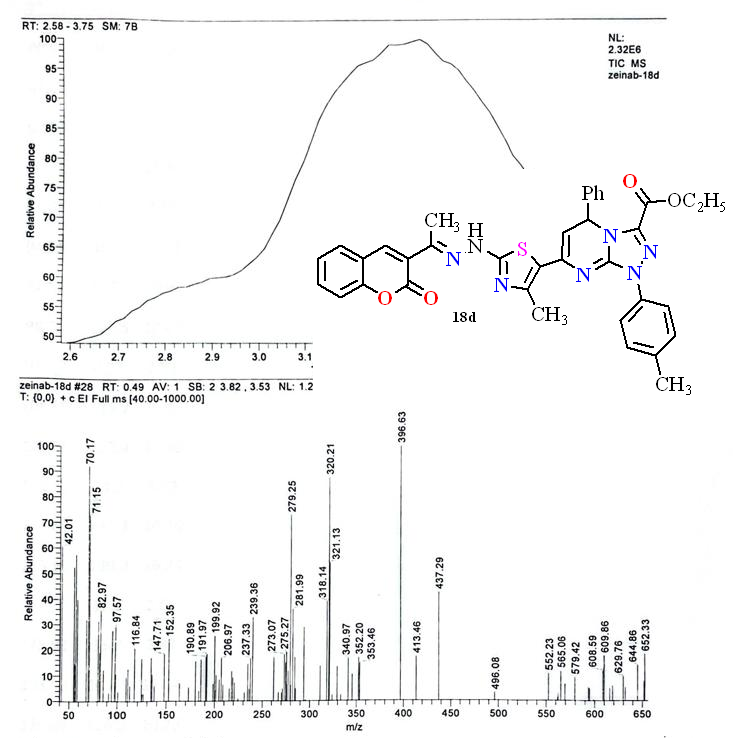


**Mass spectrum compound 18d**


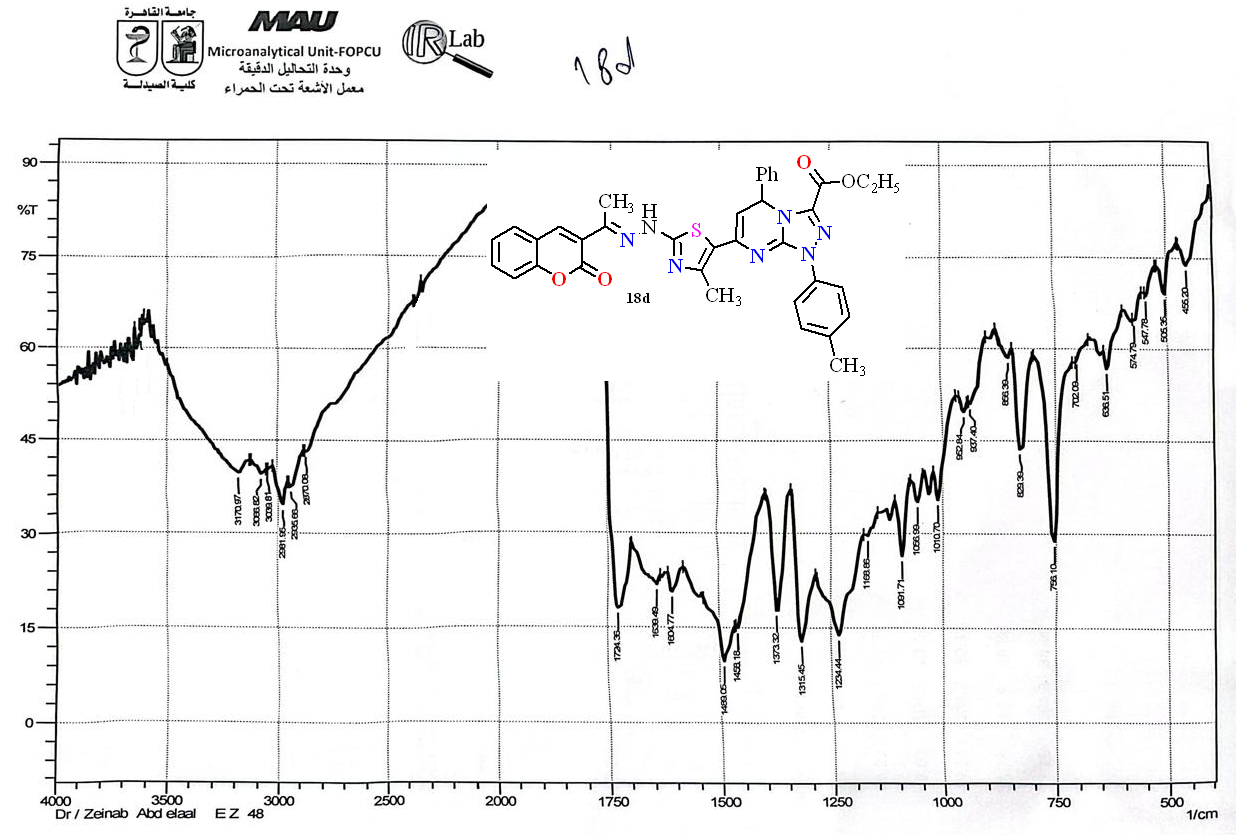


**IR spectrum compound 18d**


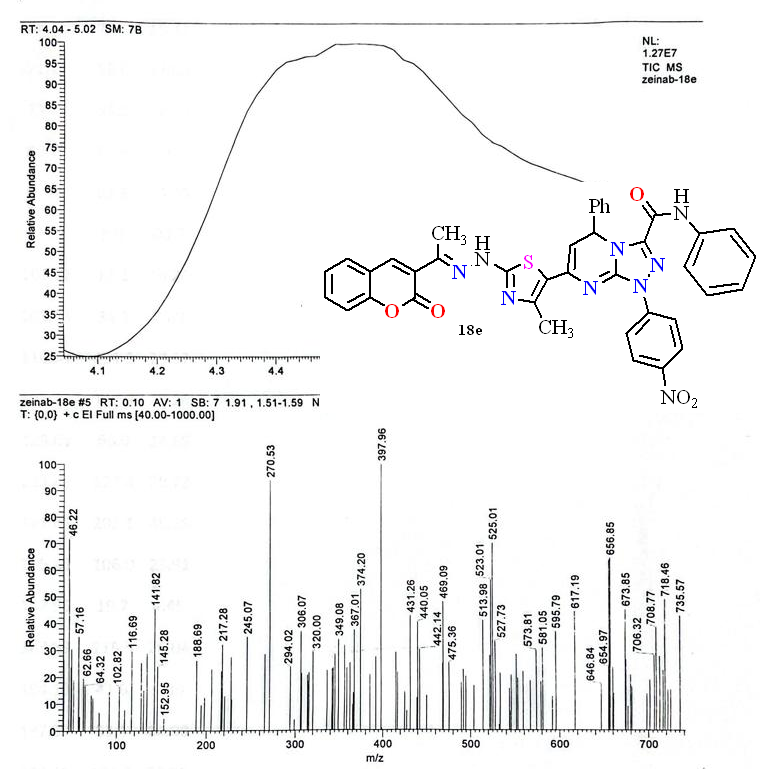


**Mass spectrum compound 18e**


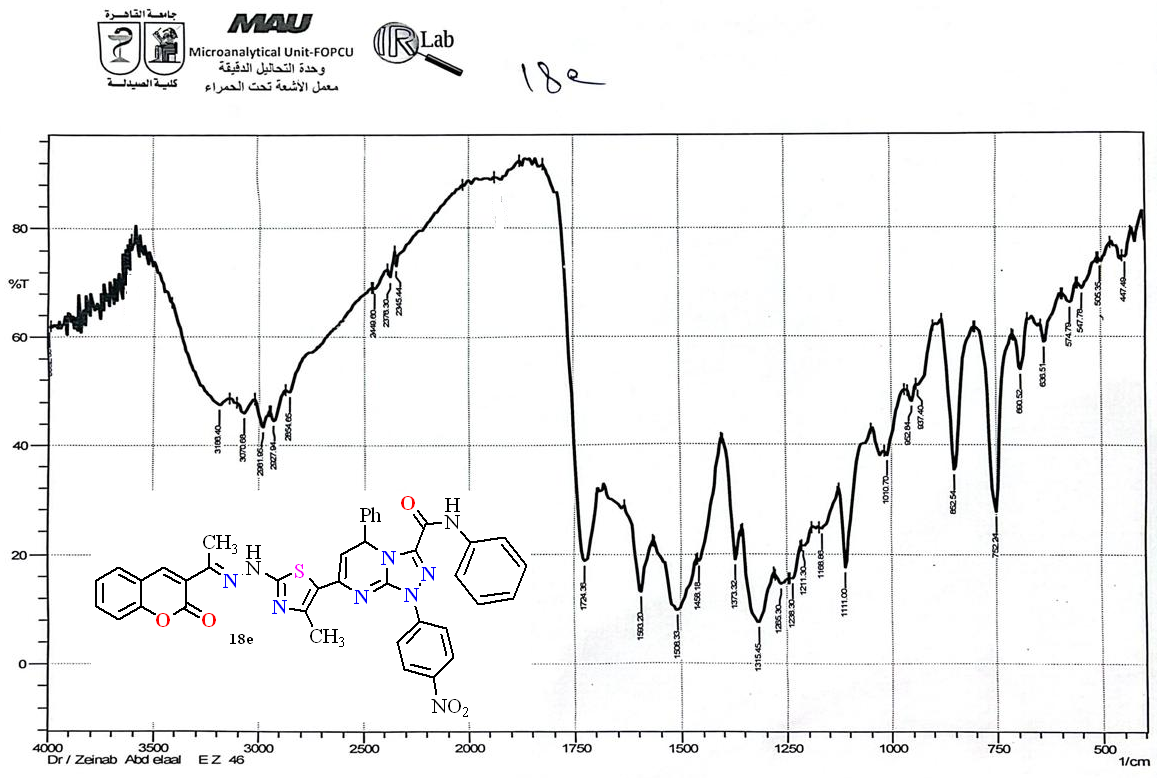


**IR spectrum compound 18e**
