# Supplementary material for: Coumarin‐Augmented Thiazole Hybrids as Dual Anticancer and Antibacterial Agents
Source: Chem Biol Drug Des. 2026 Feb 20;107(2):e70261. doi: 10.1111/cbdd.70261 (PMC12923669; doi:10.1111/cbdd.70261)
Supplement: Supplementary file 4 — Data S4: cbdd70261‐sup‐0004‐DataS4.pdf.pdf. [file CBDD-107-e70261-s003.pdf]

Al-Azhar University
The Regional Center for Mycology & Biotechnology

Evaluation of cytotoxicity against HELA cell line

Requester Data:

Name: Dr. Zinab Abdel-Aal
Sample Code: (Doxorubicin)

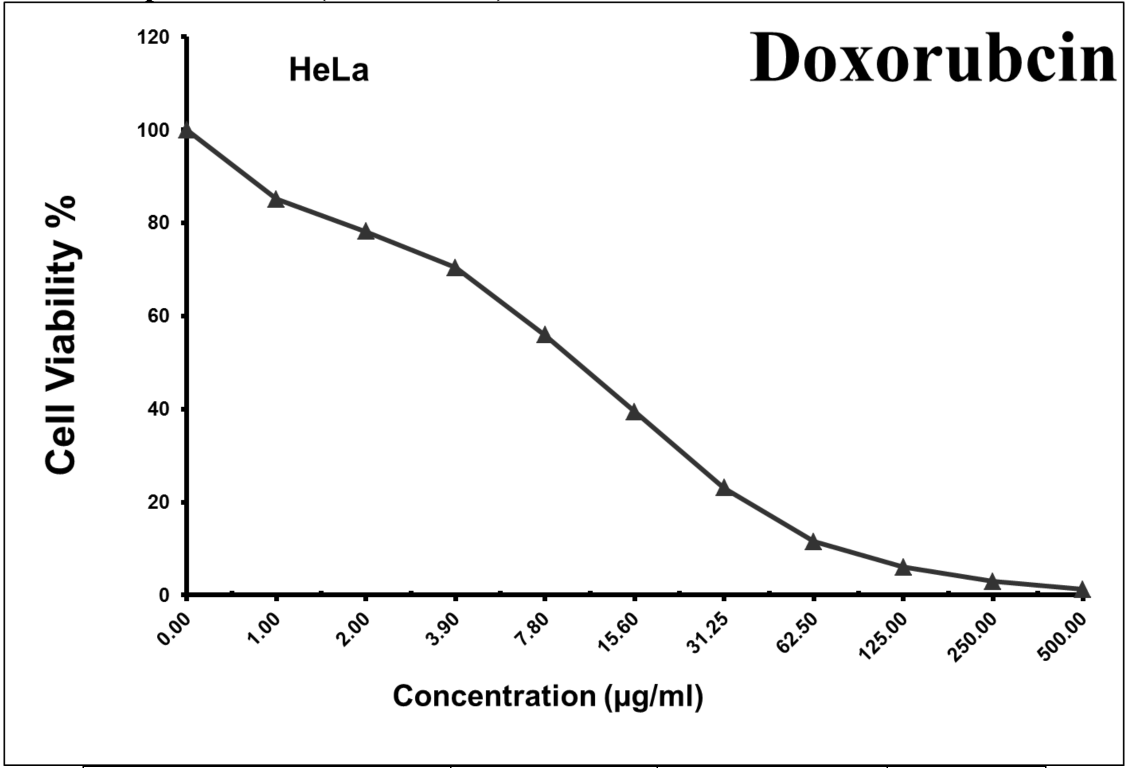

Sample conc. (µg/ml)	Viability %	Inhibitory %	S.D. (±)
500	1.23	98.77	0.15
250	2.97	97.03	0.29
125	5.98	94.02	0.16
62.5	11.48	88.52	0.68
31.25	23.09	76.91	1.27
15.6	39.43	60.57	2.01
7.8	55.92	44.08	2.84
3.9	70.41	29.59	1.03
2	78.20	21.8	0.84
1	85.13	14.87	1.25
0	100	0	

Comment:

Inhibitory activity against Cervical carcinoma cells was detected using MTT assay under these experimental conditions with IC_{50} = 10.59 ± 1.03µg/ml.

Investigator (s) **Director**

Al-Azhar University
The Regional Center for Mycology & Biotechnology

Name: **Dr. Dr. Zinab Abdel-Aal**

Sample Code	IC_{50} values (µg/ml)
	HELA
EZ 21	339.4 ± 14.29
EZ 22	263.8± 12.57
EZ 23	111.7 ± 5.94
EZ 24	97.0 ± 4.21
EZ 25	83.02 ± 3.89
EZ 26	123.1 ± 5.72
EZ 27	91.2 ± 4.78
EZ 28	186.2 ± 7.95
EZ 29	205.4 ± 9.06
EZ 30	197.5 ± 8.14
EZ 31	56.5 ± 2.83
EZ 33	76.0 ± 4.67
EZ 35	106.0 ± 6.21
EZ 36	285.2 ± 14.28
EZ 37	239.1± 10.17
EZ 38	91.7 ± 5.93
EZ 39	88.4 ± 4.87
EZ 40	54.4 ± 3.06
EZ 41	224.8 ± 9.37
EZ 42	239.3 ± 10.23
EZ 43	26.8 ± 0.97
EZ 44	37.02 ± 2.13
EZ 45	70.6 ± 4.08
EZ 46	58.9 ± 2.71
EZ 47	59.1± 2.91
EZ 48	204.3 ± 6.43
EZ 49	68.6 ± 5.08
EZ 50	47.5 ± 2.13
EZ 51	100 ± 4.69
EZ 52	52.7 ± 4.87
EZ 53	35.8 ± 2.68
EZ 54	217.0 ± 8.75
EZ 55	246.4 ± 11.24
EZ 56	37.8 ± 1.98

Results: The IC$_{50}$ values were detected using MTT assay under the experimental conditions.

EZ $_{57}$	23.8 ± 1.72
Doxorubcin Reference Drug	10.59 ± 1.03

Al-Azhar University
The Regional Center for Mycology & Biotechnology

Evaluation of cytotoxicity against HELA cell line

Requester Data:

Name: Dr. Zinab Abdel-Aal
Sample Code: (EZ 35)

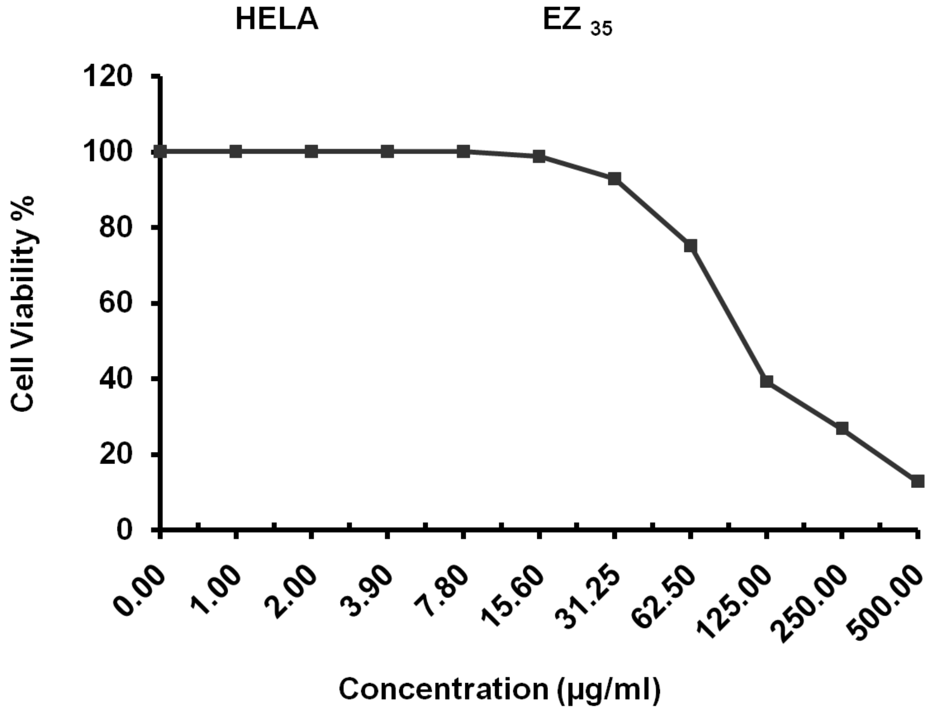

Sample conc. (µg/ml)	Viability %	Inhibitory %	S.D. (±)
500	12.69	87.31	1.37
250	26.74	73.26	2.42
125	39.08	60.92	2.94
62.5	75.03	24.97	3.15
31.25	92.71	7.29	1.03
15.6	98.69	1.31	0.75
7.8	100	0	
3.9	100	0	
2	100	0	
1	100	0	
0	100	0	

Comment:

Inhibitory activity against Cervical carcinoma cells was detected using MTT assay under these experimental conditions with $IC_{50} = 106.0 \pm 6.21$ µg/ml.

Investigator (s) **Director**

Al-Azhar University
The Regional Center for Mycology & Biotechnology

Evaluation of cytotoxicity against HELA cell line

Requester Data:

Name: Dr. Zinab Abdel-Aal
Sample Code: (EZ 36)

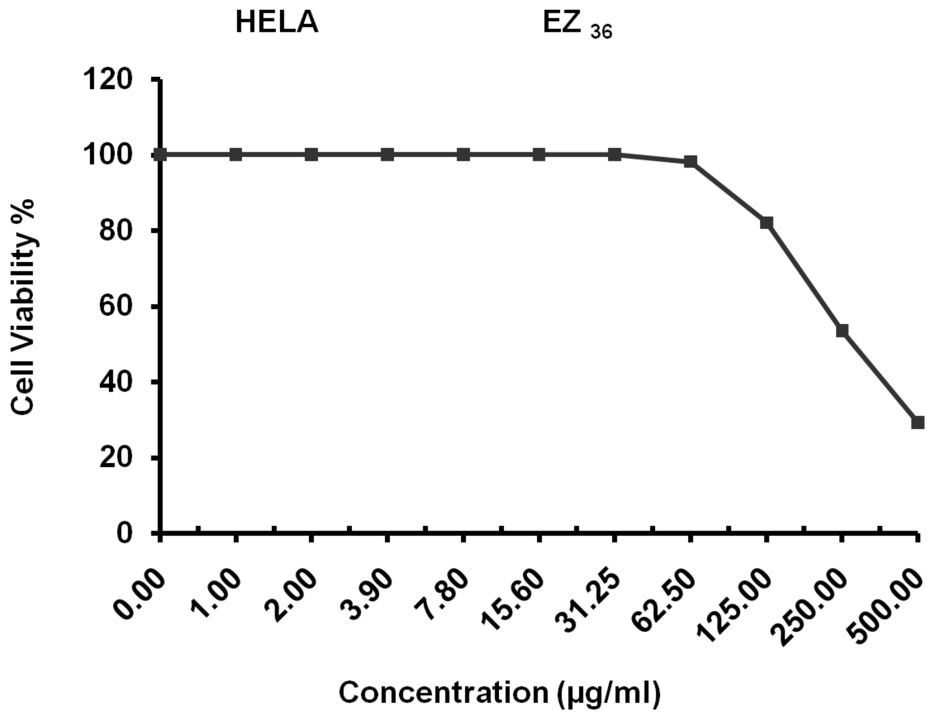

Sample conc. (µg/ml)	Viability %	Inhibitory %	S.D. (±)
500	29.17	70.83	3.29
250	53.42	46.58	2.64
125	81.97	18.03	1.59
62.5	98.06	1.94	1.32
31.25	100	0	
15.6	100	0	
7.8	100	0	
3.9	100	0	
2	100	0	
1	100	0	
0	100	0	

Comment:

Inhibitory activity against Cervical carcinoma cells was detected using MTT assay under these experimental conditions with IC_{50} = 285.2 ± 14.28 µg/ml.

Investigator (s) **Director**

Al-Azhar University
The Regional Center for Mycology & Biotechnology

Evaluation of cytotoxicity against HELA cell line

Requester Data:

Name: **Dr. Zinab Abdel-Aal**
Sample Code: **(EZ 37)**

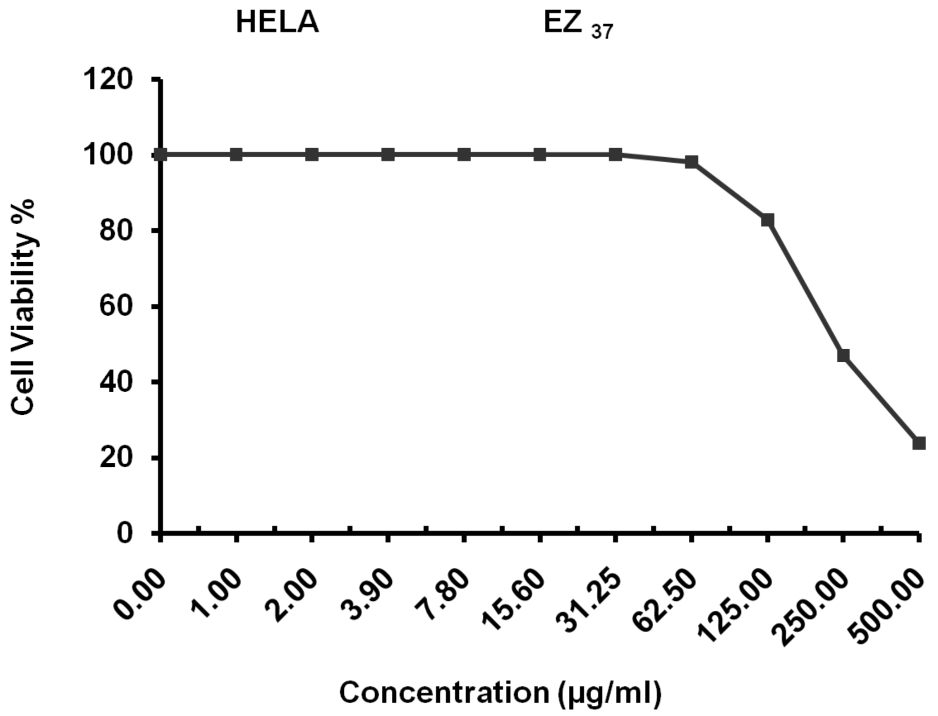

Sample conc. (µg/ml)	Viability %	Inhibitory %	S.D. (±)
500	23.65	76.35	1.37
250	46.91	53.09	2.53
125	82.63	17.37	2.41
62.5	98.04	1.96	0.82
31.25	100	0	
15.6	100	0	
7.8	100	0	
3.9	100	0	
2	100	0	
1	100	0	
0	100	0	

Comment:

Inhibitory activity against Cervical carcinoma cells was detected using MTT assay under these experimental conditions with IC_{50} = 239.1± 10.17 µg/ml.

Investigator (s) **Director**

Al-Azhar University
The Regional Center for Mycology & Biotechnology

Evaluation of cytotoxicity against HELA cell line

Requester Data:

Name: Dr. Zinab Abdel-Aal
Sample Code: (EZ 38)

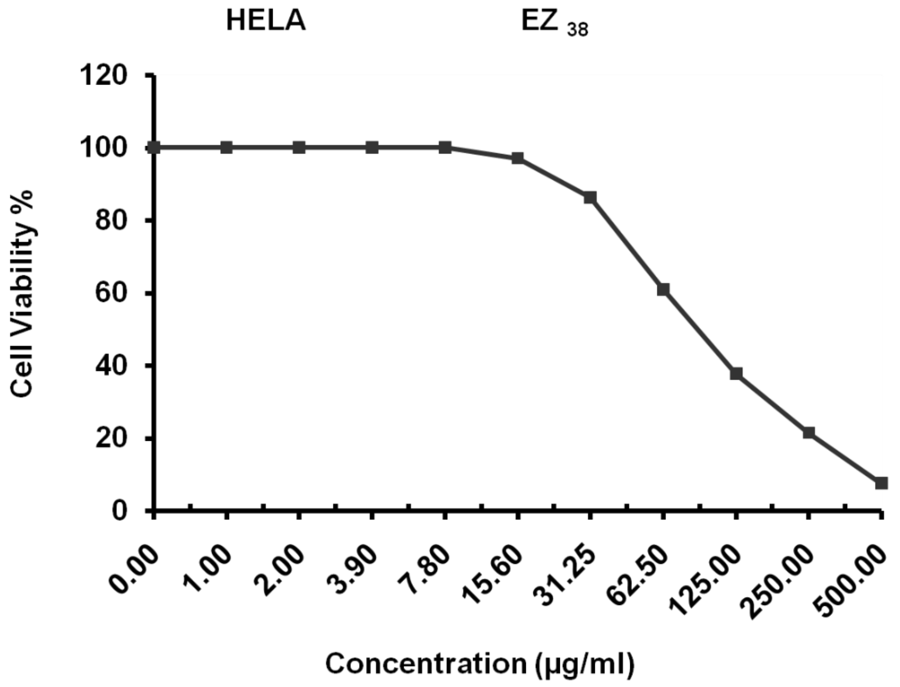

Sample conc. (µg/ml)	Viability %	Inhibitory %	S.D. (±)
500	7.46	92.54	0.54
250	21.35	78.65	1.39
125	37.61	62.39	2.57
62.5	60.89	39.11	3.12
31.25	86.23	13.77	0.95
15.6	97.04	2.96	0.68
7.8	100	0	
3.9	100	0	
2	100	0	
1	100	0	
0	100	0	

Comment:

Inhibitory activity against Cervical carcinoma cells was detected using MTT assay under these experimental conditions with IC_{50} = 91.7 ± 5.93 µg/ml.

Investigator (s) **Director**

Al-Azhar University
The Regional Center for Mycology & Biotechnology

Evaluation of cytotoxicity against HELA cell line

Requester Data:

Name: Dr. Zinab Abdel-Aal
Sample Code: (EZ 39)

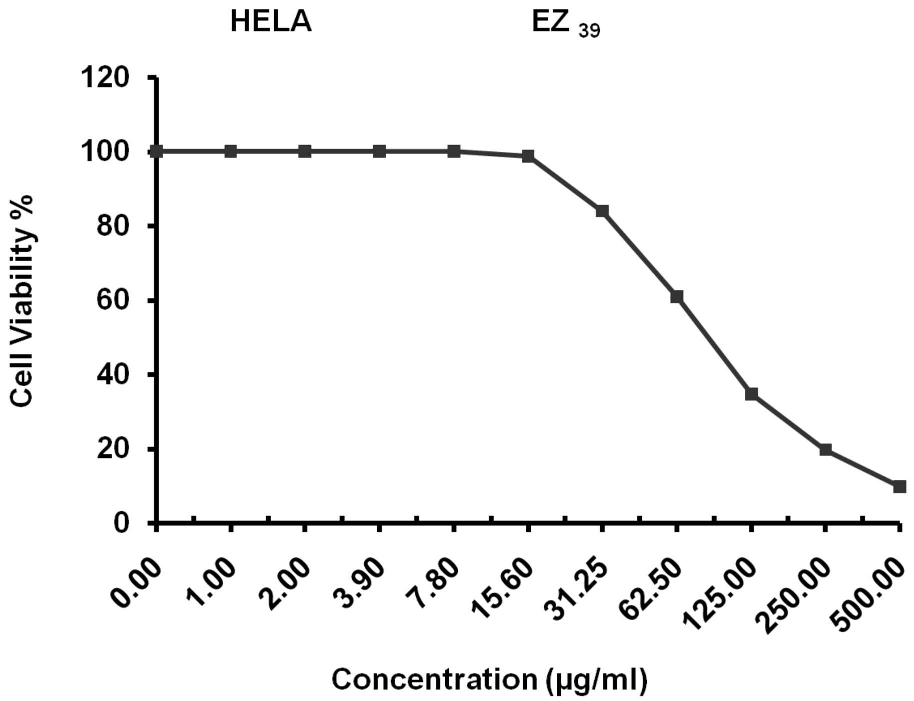

Sample conc. (µg/ml)	Viability %	Inhibitory %	S.D. (±)
500	9.82	90.18	0.64
250	19.75	80.25	1.39
125	34.67	65.33	2.09
62.5	60..85	39.15	1.57
31.25	83.91	16.09	2.35
15.6	98.62	1.38	1.04
7.8	100	0	
3.9	100	0	
2	100	0	
1	100	0	
0	100	0	

Comment:

Inhibitory activity against Cervical carcinoma cells was detected using MTT assay under these experimental conditions with $IC_{50} = 88.4 \pm 4.87$ µg/ml.

Investigator (s) **Director**

Al-Azhar University
The Regional Center for Mycology & Biotechnology

Evaluation of cytotoxicity against HELA cell line

Requester Data:

Name: Dr. Zinab Abdel-Aal
Sample Code: (EZ $_{40}$)

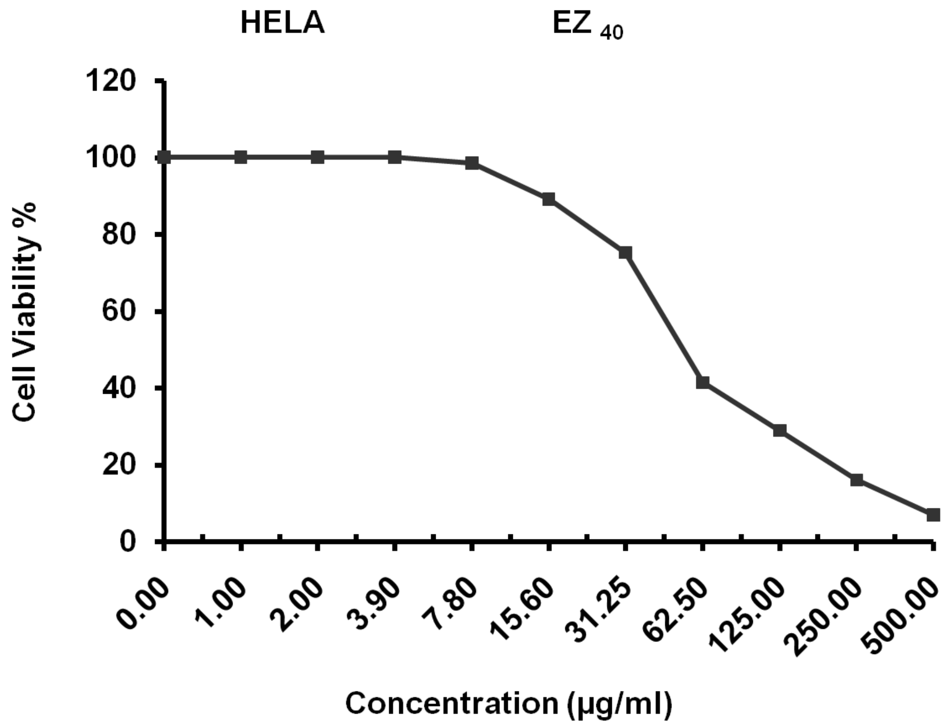

Sample conc. (µg/ml)	Viability %	Inhibitory %	S.D. (±)
500	6.81	93.19	0.37
250	15.92	84.08	1.46
125	28.79	71.21	2.03
62.5	41.28	58.72	1.56
31.25	75.13	24.87	2.39
15.6	89.04	10.96	1.22
7.8	98.41	1.59	0.83
3.9	100	0	
2	100	0	
1	100	0	
0	100	0	

Comment:

Inhibitory activity against Cervical carcinoma cells was detected using MTT assay under these experimental conditions with IC_{50} = 54.4 ± 3.06 µg/ml.

Investigator (s) **Director**

Al-Azhar University
The Regional Center for Mycology & Biotechnology

Evaluation of cytotoxicity against HELA cell line

Requester Data:

Name: Dr. Zinab Abdel-Aal
Sample Code: (EZ 41)

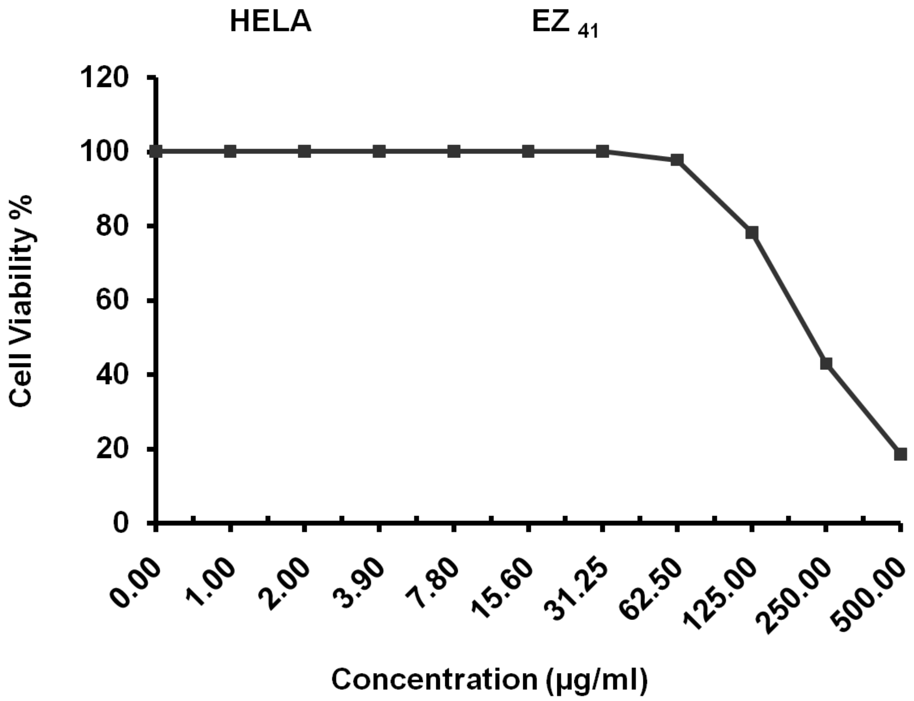

Sample conc. (µg/ml)	Viability %	Inhibitory %	S.D. (±)
500	18.46	81.54	2.32
250	42.89	57.11	2.97
125	78.17	21.83	1.85
62.5	97.65	2.35	0.93
31.25	100	0	
15.6	100	0	
7.8	100	0	
3.9	100	0	
2	100	0	
1	100	0	
0	100	0	

Comment:

Inhibitory activity against Cervical carcinoma cells was detected using MTT assay under these experimental conditions with IC_{50} = 224.8 ± 9.37µg/ml.

Investigator (s) **Director**

Al-Azhar University
The Regional Center for Mycology & Biotechnology

Evaluation of cytotoxicity against HELA cell line

Requester Data:

Name: Dr. Zinab Abdel-Aal
Sample Code: (EZ 42)

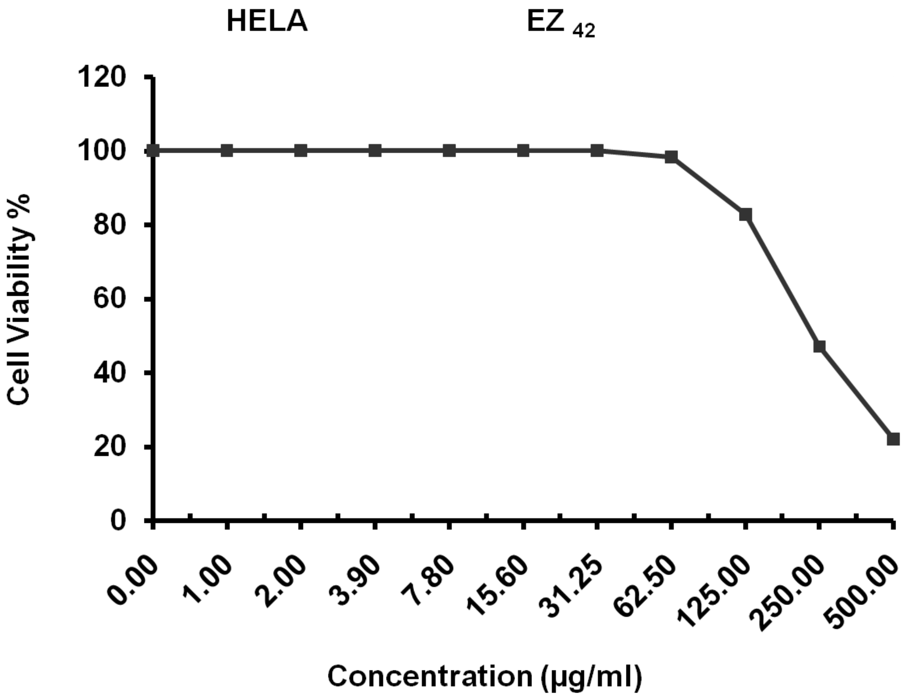

Sample conc. (µg/ml)	Viability %	Inhibitory %	S.D. (±)
500	21.89	78.11	1.37
250	46.95	53.05	2.09
125	82.63	17.37	2.15
62.5	98.17	1.83	1.21
31.25	100	0	
15.6	100	0	
7.8	100	0	
3.9	100	0	
2	100	0	
1	100	0	
0	100	0	

Comment:

Inhibitory activity against Cervical carcinoma cells was detected using MTT assay under these experimental conditions with IC_{50} = 239.3 ± 10.23µg/ml.

Investigator (s) **Director**

Al-Azhar University
The Regional Center for Mycology & Biotechnology

Evaluation of cytotoxicity against HELA cell line

Requester Data:

Name: Dr. Zinab Abdel-Aal
Sample Code: (EZ 43)

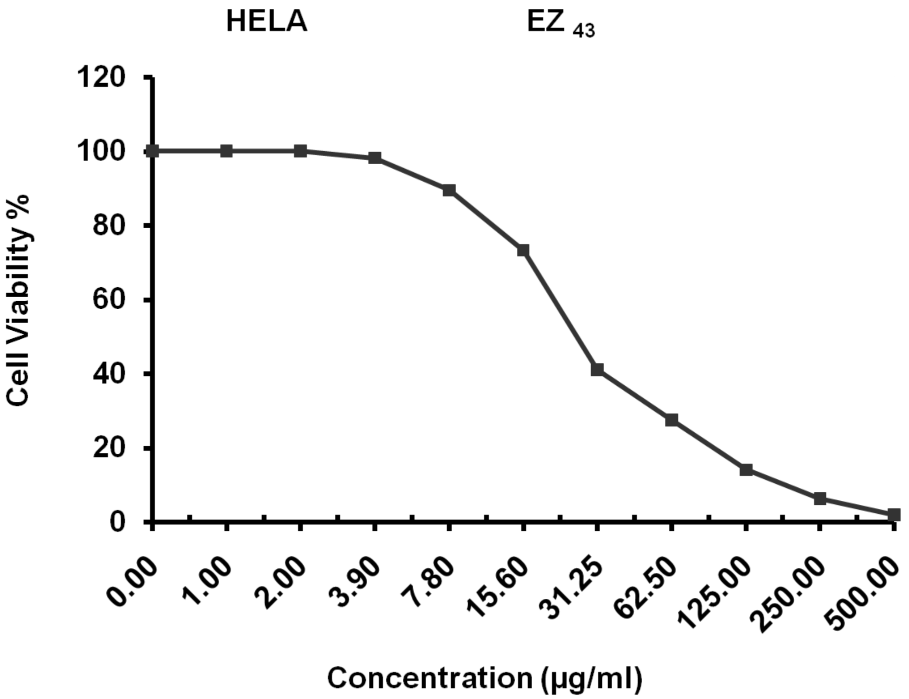

Sample conc. (µg/ml)	Viability %	Inhibitory %	S.D. (±)
500	1.79	98.21	0.63
250	6.24	93.76	0.52
125	13.95	86.05	0.79
62.5	27.39	72.61	1.25
31.25	40.91	59.09	1.37
15.6	73.18	26.82	2.06
7.8	89.40	10.6	1.28
3.9	98.06	1.94	0.64
2	100	0	
1	100	0	
0	100	0	

Comment:

Inhibitory activity against Cervical carcinoma cells was detected using MTT assay under these experimental conditions with $IC_{50} = 26.8 \pm 0.97$ µg/ml.

Investigator (s) **Director**

Al-Azhar University
The Regional Center for Mycology & Biotechnology

Evaluation of cytotoxicity against HELA cell line

Requester Data:

Name: Dr. Zinab Abdel-Aal

Sample Code: (EZ 44)

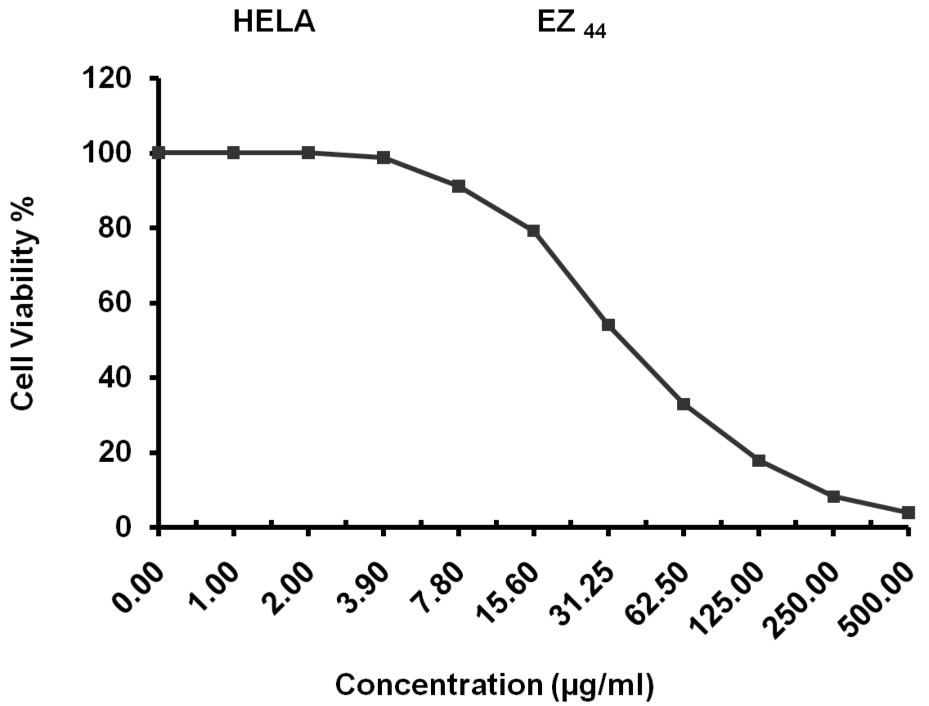

Sample conc. (µg/ml)	Viability %	Inhibitory %	S.D. (±)
500	3.75	96.25	0.31
250	8.04	91.96	0.62
125	17.69	82.31	1.03
62.5	32.76	67.24	2.82
31.25	53.91	46.09	2.97
15.6	79.05	20.95	2.31
7.8	91.05	8.95	1.09
3.9	98.60	1.4	0.32
2	100	0	
1	100	0	
0	100	0	

Comment:

Inhibitory activity against Cervical carcinoma cells was detected using MTT assay under these experimental conditions with $IC_{50} = 37.02 \pm 2.13$ µg/ml.

Investigator (s) **Director**

Al-Azhar University
The Regional Center for Mycology & Biotechnology

Evaluation of cytotoxicity against HELA cell line

Requester Data:

Name: **Dr. Zinab Abdel-Aal**
Sample Code: **(EZ 45)**

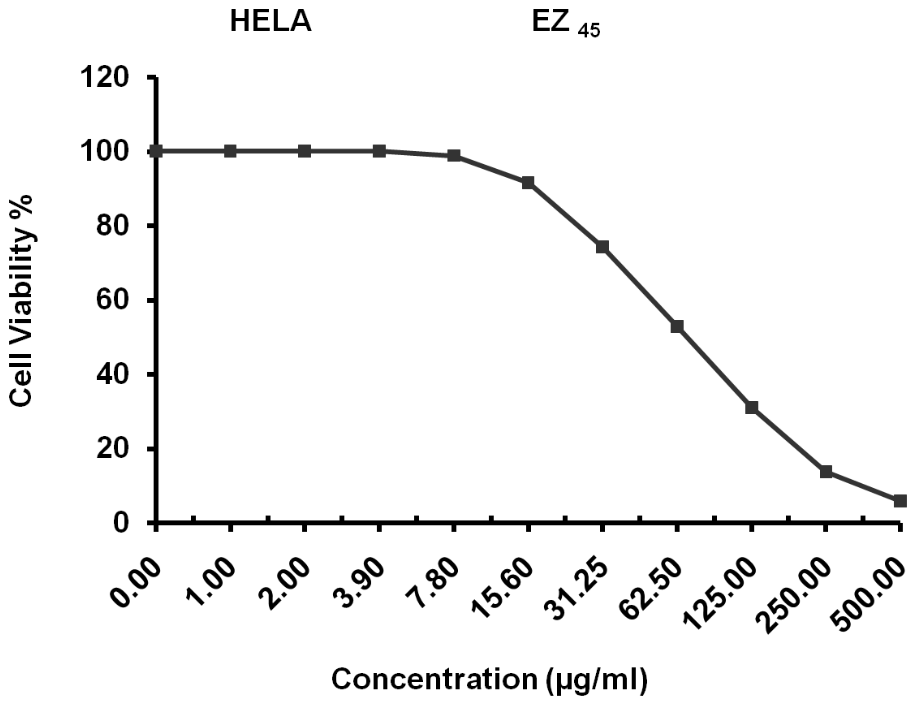

Sample conc. (µg/ml)	Viability %	Inhibitory %	S.D. (±)
500	5.82	94.18	0.24
250	13.69	86.31	1.07
125	30.91	69.09	2.35
62.5	52.85	47.15	2.13
31.25	74.18	25.82	1.26
15.6	91.43	8.57	0.79
7.8	98.75	1.25	0.23
3.9	100	0	
2	100	0	
1	100	0	
0	100	0	

Comment:

Inhibitory activity against Cervical carcinoma cells was detected using MTT assay under these experimental conditions with IC_{50} = 70.6 ± 4.08 µg/ml.

Investigator (s) **Director**

Al-Azhar University
The Regional Center for Mycology & Biotechnology

Evaluation of cytotoxicity against HELA cell line

Requester Data:

Name: Dr. Zinab Abdel-Aal
Sample Code: (EZ 46)

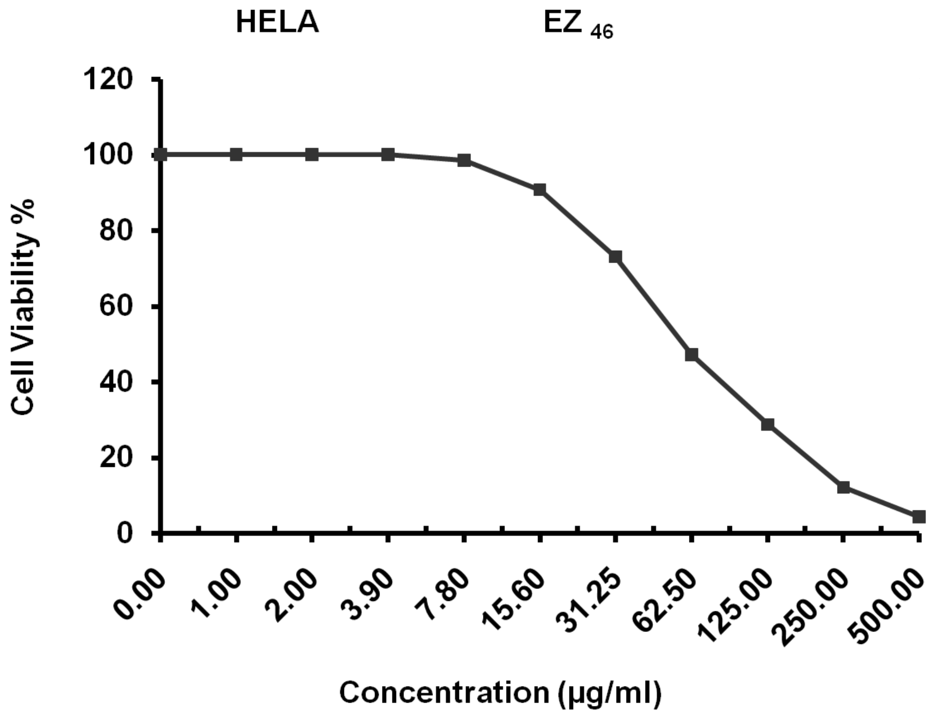

Sample conc. (µg/ml)	Viability %	Inhibitory %	S.D. (±)
500	4.21	95.79	0.35
250	11.98	88.02	0.34
125	28.63	71.37	0.91
62.5	47.06	52.94	2.32
31.25	72.95	27.05	1.79
15.6	90.67	9.33	0.85
7.8	98.43	1.57	0.11
3.9	100	0	
2	100	0	
1	100	0	
0	100	0	

Comment:

Inhibitory activity against Cervical carcinoma cells was detected using MTT assay under these experimental conditions with IC_{50} = 58.9 ± 2.71µg/ml.

Investigator (s) **Director**

Al-Azhar University
The Regional Center for Mycology & Biotechnology

Evaluation of cytotoxicity against HELA cell line

Requester Data:

Name: **Dr. Zinab Abdel-Aal**
Sample Code: **(EZ 47)**

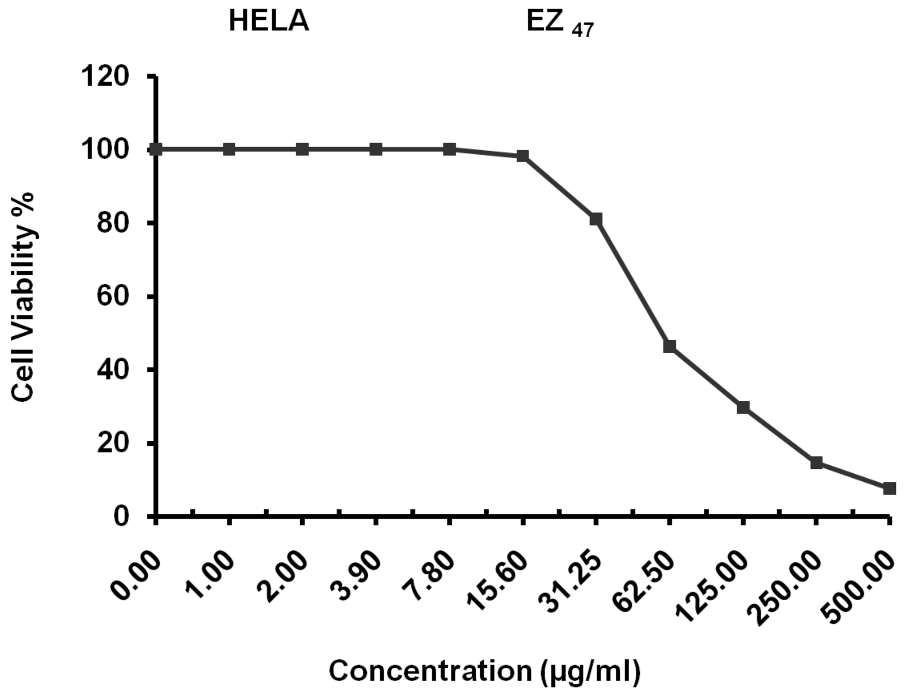

Sample conc. (µg/ml)	Viability %	Inhibitory %	S.D. (±)
500	7.58	92.42	0.44
250	14.63	85.37	0.95
125	29.71	70.29	1.37
62.5	46.28	53.72	2.46
31.25	80.96	19.04	1.82
15.6	98.13	1.87	0.95
7.8	100	0	
3.9	100	0	
2	100	0	
1	100	0	
0	100	0	

Comment:

Inhibitory activity against Cervical carcinoma cells was detected using MTT assay under these experimental conditions with IC_{50} = 59.1± 2.91µg/ml.

Investigator (s) **Director**

Al-Azhar University
The Regional Center for Mycology & Biotechnology

Evaluation of cytotoxicity against HELA cell line

Requester Data:

Name: Dr. Zinab Abdel-Aal
Sample Code: (EZ 48)

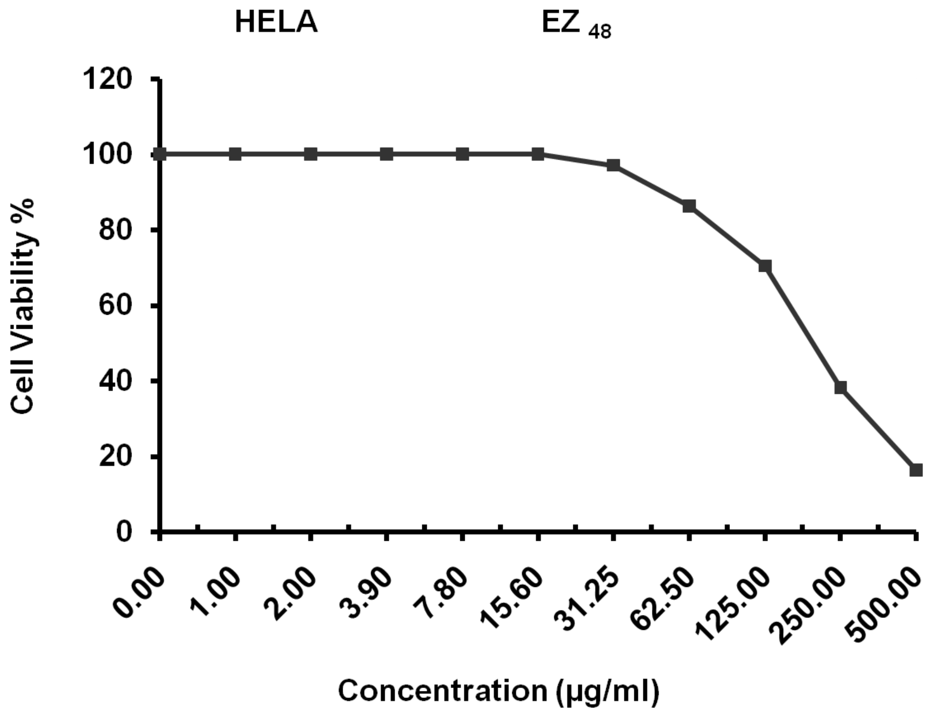

Sample conc. (µg/ml)	Viability %	Inhibitory %	S.D. (±)
500	16.35	83.65	1.49
250	38.26	61.74	2.32
125	70.39	29.61	3.17
62.5	86.24	13.76	1.32
31.25	97.02	2.98	0.84
15.6	100	0	
7.8	100	0	
3.9	100	0	
2	100	0	
1	100	0	
0	100	0	

Comment:

Inhibitory activity against Cervical carcinoma cells was detected using MTT assay under these experimental conditions with IC_{50} = 204.3 ± 6.43 µg/ml.

Investigator (s) **Director**

Al-Azhar University
The Regional Center for Mycology & Biotechnology

Evaluation of cytotoxicity against HELA cell line

Requester Data:

Name: Dr. Zinab Abdel-Aal

Sample Code: (EZ 49)

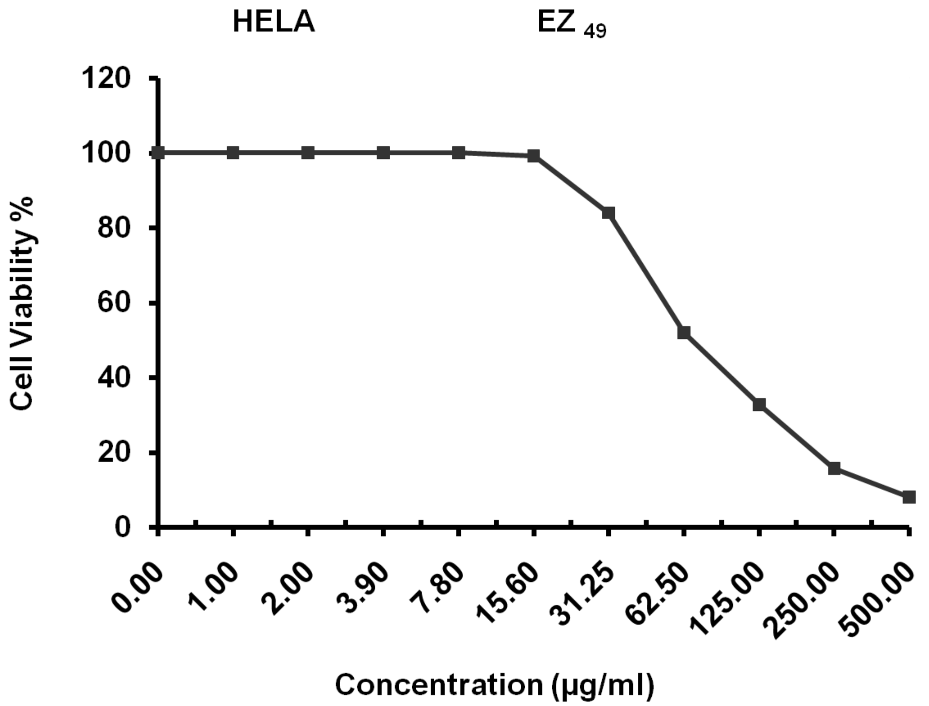

Sample conc. (µg/ml)	Viability %	Inhibitory %	S.D. (±)
500	8.02	91.98	0.64
250	15.75	84.25	0.81
125	32.69	67.31	1.73
62.5	51.88	48.12	2.06
31.25	83.87	16.13	1.59
15.6	99.04	0.96	0.62
7.8	100	0	
3.9	100	0	
2	100	0	
1	100	0	
0	100	0	

Comment:

Inhibitory activity against Cervical carcinoma cells was detected using MTT assay under these experimental conditions with IC_{50} = 68.6 ± 5.08 µg/ml.

Investigator (s) **Director**

Al-Azhar University
The Regional Center for Mycology & Biotechnology

Evaluation of cytotoxicity against HELA cell line

Requester Data:

Name: Dr. Zinab Abdel-Aal
Sample Code: (EZ 50)

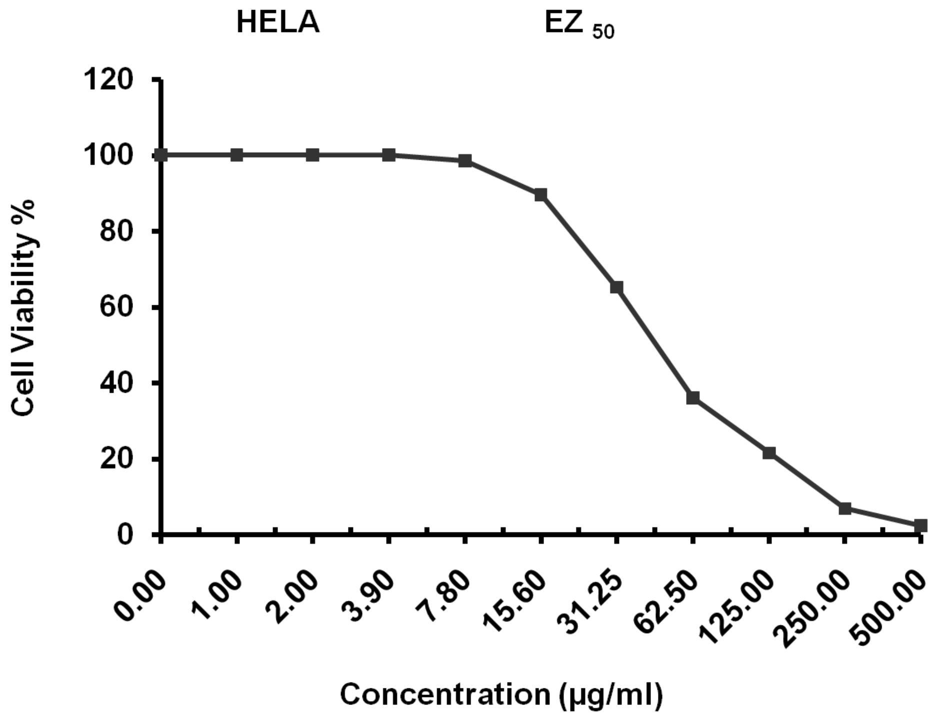

Sample conc. (µg/ml)	Viability %	Inhibitory %	S.D. (±)
500	2.37	97.63	0.21
250	6.85	93.15	0.23
125	21.49	78.51	0.75
62.5	36.04	63.96	1.82
31.25	65.12	34.88	2.34
15.6	89.43	10.57	1.79
7.8	98.41	1.59	0.63
3.9	100	0	
2	100	0	
1	100	0	
0	100	0	

Comment:

Inhibitory activity against Cervical carcinoma cells was detected using MTT assay under these experimental conditions with IC_{50} = 47.5 ± 2.13µg/ml.

Investigator (s) **Director**

Al-Azhar University
The Regional Center for Mycology & Biotechnology

Evaluation of cytotoxicity against HELA cell line

Requester Data:

Name: Dr. Zinab Abdel-Aal
Sample Code: (EZ 51)

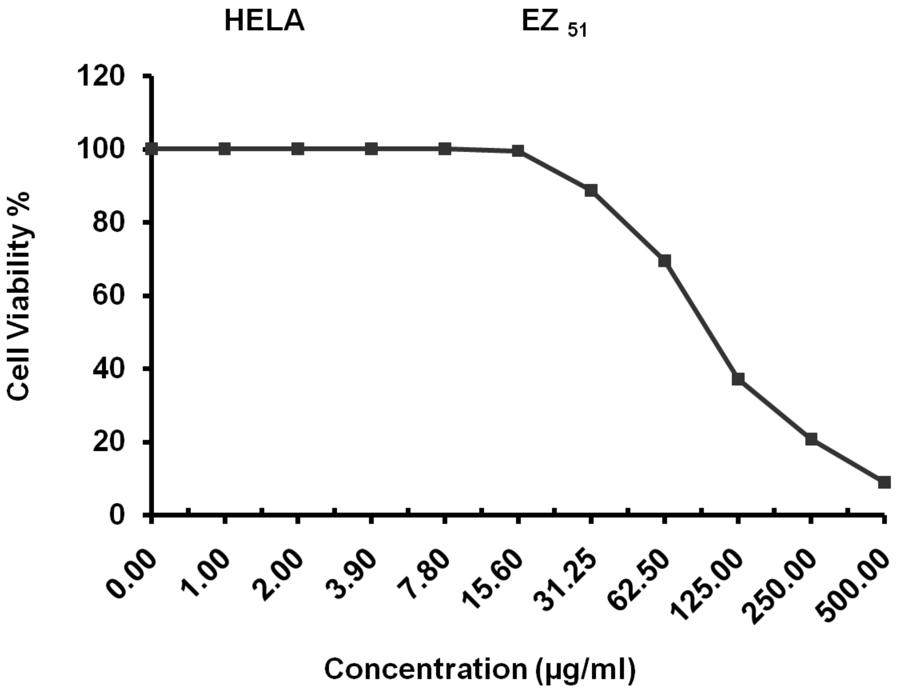

Sample conc. (µg/ml)	Viability %	Inhibitory %	S.D. (±)
500	8.78	91.22	0.64
250	20.69	79.31	1.33
125	37.06	62.94	2.12
62.5	69.41	30.59	1.79
31.25	88.65	11.35	0.93
15.6	99.43	0.57	0.15
7.8	100	0	
3.9	100	0	
2	100	0	
1	100	0	
0	100	0	

Comment:

Inhibitory activity against Cervical carcinoma cells was detected using MTT assay under these experimental conditions with IC_{50} = 100 ± 4.69 µg/ml.

Investigator (s) **Director**

Evaluation of Cytotoxic Effects of certain Chemical compound

Mammalian cell lines: HeLa cells (Cervical carcinoma cells). were obtained from the American Type Culture Collection (ATCC, Rockville, MD).

Chemicals Used: Dimethyl sulfoxide (DMSO), MTT and trypan blue dye were purchased from Sigma (St. Louis, Mo., USA).

Fetal Bovine serum, DMEM, RPMI-1640, HEPES buffer solution, L-glutamine, gentamycin and 0.25% Trypsin-EDTA were purchased from Lonza (Belgium).

Cell line Propagation:

The cells were grown on RPMI-1640 medium supplemented with 10% inactivated fetal calf serum and 50µg/ml gentamycin. The cells were maintained at 37ºC in a humidified atmosphere with 5% CO2 and were subcultured two to three times a week.

Cytotoxicity evaluation using viability assay: For antitumor assays, the tumor cell lines were suspended in medium at concentration $5x10^4$ cell/well in Corning® 96-well tissue culture plates, then incubated for 24 hr. The tested compounds were then added into 96-well plates (three replicates) to achieve twelve concentrations for each compound. Six vehicle controls with media or 0.5 % DMSO were run for each 96 well plate as a control. After incubating for 24 h, the numbers of viable cells were determined by the MTT test. Briefly, the media was removed from the 96 well plate and replaced with 100 µl of fresh culture RPMI 1640 medium without phenol red then 10 µl of the 12 mM MTT stock solution (5 mg of MTT in 1 mL of PBS) to each well including the untreated controls. The 96 well plates were then incubated at 37°C and 5% CO_2 for 4 hours. An 85 µl aliquot of the media was removed from the wells, and 50 µl of DMSO was added to each well and mixed thoroughly with the pipette and incubated at 37°C for 10 min. Then, the optical density was measured at 590 nm with the microplate reader (SunRise, TECAN, Inc, USA) to determine the number of viable cells and the percentage of viability was calculated as [(ODt/ODc)]x100% where ODt is the mean optical density of wells treated with the tested sample and ODc is the mean optical density of untreated cells. The relation between surviving cells and drug concentration is plotted to get the survival curve of each tumor cell line after treatment with the specified compound. The 50% inhibitory concentration (IC_{50}), the concentration required to cause toxic effects in 50% of intact cells, was estimated from graphic plots of the dose response curve for each conc. using Graphpad Prism software (San Diego, CA. USA) (**Mosmann, 1983).**

References:

Mosmann, T. (1983): Rapid colorimetric assay for cellular growth and survival: application to proliferation and cytotoxicity assays. J. Immunol. Methods; 65: 55-63.

Gomha, S.M.; Riyadh, S.M.; Mahmmoud, E.A. and Elaasser, M.M. (2015): Synthesis and Anticancer Activities of Thiazoles, 1,3-Thiazines, and Thiazolidine Using Chitosan-Grafted-Poly(vinylpyridine) as Basic Catalyst. Heterocycles; 91(6):1227-1243.
